# Estrogen Alleviates Sevoflurane‐Induced Neurotoxicity by Inhibiting ERα‐Tau Binding

**DOI:** 10.1002/advs.202508568

**Published:** 2025-09-06

**Authors:** Feixiang Li, Bingqing Gong, Zichen Song, Nuo Yang, Yuming Liu, Jinqin Zhang, Dujuan Li, Yongyan Yang, Yonghao Yu

**Affiliations:** ^1^ Department of Anesthesiology Tianjin Medical University General Hospital Tianjin Institute of Anesthesiology Tianjin 300052 China; ^2^ Department of Rehabilitation Medicine The Second Affiliated Hospital of Wannan Medical College Wuhu 241000 China; ^3^ Department of Anesthesiology Beijing Chao‐Yang Hospital Capital Medical University Beijing 100020 China

**Keywords:** aged mice, ERα, estrogen, neuroprotective, sevoflurane

## Abstract

Sevoflurane‐induced neurotoxicity is age‐dependent, but the role of sex differences is unclear. While testosterone has protective effects, the impact of estrogen remains unknown. This study investigates the effects of sevoflurane on neurotoxicity in adult, middle‐aged, and aged male and female mice. Neurotoxic effects are assessed through Tau phosphorylation, cognitive function, transient Ca^2^⁺ signal amplitude, firing frequency, dendritic spine density, and dendritic axon count in the dorsal hippocampal CA1 (dCA1) region. Estrogen receptor antagonists, inhibitors, and overexpression viral vectors are used to explore estrogen receptor‐mediated actions. Protein–protein interactions are analyzed using GRAMM docking and ITC, and competitive ELISA is employed to investigate estrogen's mechanisms. Results show that sevoflurane induces neurotoxicity in middle‐aged female mice but not in males. Estrogen alleviates Tau phosphorylation, cognitive impairments, and reduces Ca^2^⁺ signal amplitude, firing frequency, dendritic spine density, and dendritic shaft numbers in middle‐aged females. This protective effect is abolished with ERα knockout. In aged females, estrogen alone does not reverse neurotoxicity unless ERα expression is upregulated. Moreover, estrogen and Tau competitively bind to ERα, and sevoflurane exposure enhances this interaction. These findings suggest that estrogen mitigates sevoflurane‐induced neurotoxicity through modulation of the ERα‐Tau interaction.

## Graphical abstract

Sevoflurane‐induced neurotoxicity exhibits sex differences, and estrogen can mitigate these effects; however, its protective action appears to be time‐window dependent, which may be related to the competitive binding of estrogen and Tau to ERα.

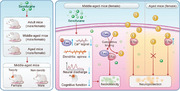

## 1 Introduction

Sevoflurane is a widely used inhalational anesthetic in clinical practice, recognized for its rapid induction and recovery. However, accumulating preclinical evidence suggests its potential neurotoxic effects on the central nervous system (CNS), with a marked age‐dependent pattern.^[^ ^1^ ^]^ Experimental studies have demonstrated that exposure to sevoflurane during the critical developmental period and advanced aging can lead to significant neuropathological changes, including impaired learning and memory consolidation, reduced synaptic plasticity, increased neuronal apoptosis, and abnormally elevated Tau phosphorylation.^[^ ^2^, ^3^ ^]^ Notably, such adverse effects are not typically observed in mature adult subjects under similar exposure conditions.^[^ ^4^ ^]^ Some studies have shown that sevoflurane‐induced neurotoxicity exhibits sex differences during critical periods of brain development.^[^ ^5^, ^6^ ^]^ However, whether such sex‐specific effects persist in the mature brain remains unclear.

Differences in sex hormones are considered the primary cause of sex‐related differences. Sex hormones play a pivotal role not only in the development and maintenance of the reproductive system but also in regulating CNS function.^[^ ^7^ ^]^ Accumulating evidence indicates that sex hormones, particularly estrogen and testosterone, can modulate neuronal plasticity, promote neuronal survival, and influence cognitive function and emotional regulation.^[^ ^8^, ^9^ ^]^ Notably, estrogen is not only secreted by the ovaries but can also be locally synthesized within the hippocampus, where it participates in the regulation of neuronal activity and synaptic transmission, particularly through actions involving the endoplasmic reticulum.^[^ ^10^, ^11^ ^]^ Our previous research demonstrated that reduced testosterone levels increase susceptibility to sevoflurane‐induced neurotoxicity, whereas testosterone supplementation mitigates such neuronal damage.^[^ ^12^, ^13^ ^]^ Therefore, it is reasonable to speculate that hippocampal estrogen levels in mice may influence the susceptibility to sevoflurane‐induced neurotoxicity and contribute to its potential neuroprotective effects.

The neuroprotective effects of estrogen are well recognized; however, its therapeutic efficacy is not universally consistent. Clinical studies have shown that the effectiveness of hormone replacement therapy (HRT) in improving cognitive function largely depends on the timing of intervention.^[^ ^14^, ^15^ ^]^ Initiating HRT during the early postmenopausal period can significantly reduce the risk of Alzheimer's disease (AD) and enhance cognitive performance. In contrast, starting HRT more than 10 years after menopause is associated with diminished neuroprotective effects and may even increase the risk of cardiovascular disease and dementia.^[^ ^16^ ^]^ This phenomenon is known as the “Critical Window Hypothesis.”^[^ ^17^, ^18^ ^]^ Whether the neuroprotective effects of estrogen against sevoflurane‐induced neurotoxicity also follow a time‐dependent pattern remains unclear.

Estrogen‐mediated neuroprotection has been shown to primarily operate through two canonical receptor subtypes: estrogen receptor alpha (ERα) and estrogen receptor beta (ERβ).^[^ ^19^ ^]^ However, due to differences in their tissue distribution and signaling mechanisms, it remains unclear which receptor predominantly mediates estrogen's protective effects against sevoflurane‐induced neurotoxicity. Therefore, this study aims to investigate whether sevoflurane‐induced neurotoxicity exhibits sex‐specific differences in developmentally mature individuals, and whether estrogen can effectively attenuate the neuronal damage caused by sevoflurane, and through which receptor this neuroprotection is mediated. Given our previous findings that sevoflurane markedly increases Tau phosphorylation in the hippocampus of mice, we further hypothesize that abnormal Tau may interfere with the binding of estrogen to its receptors, thereby attenuating its neuroprotective effects.

## 2 Results

### 2.1 Sevoflurane Selectively Increased Hippocampal Tau Phosphorylation and Cognitive Deficits in Middle‐Aged Female Mice, but not in Age‐Matched Males

**Figure 1:**
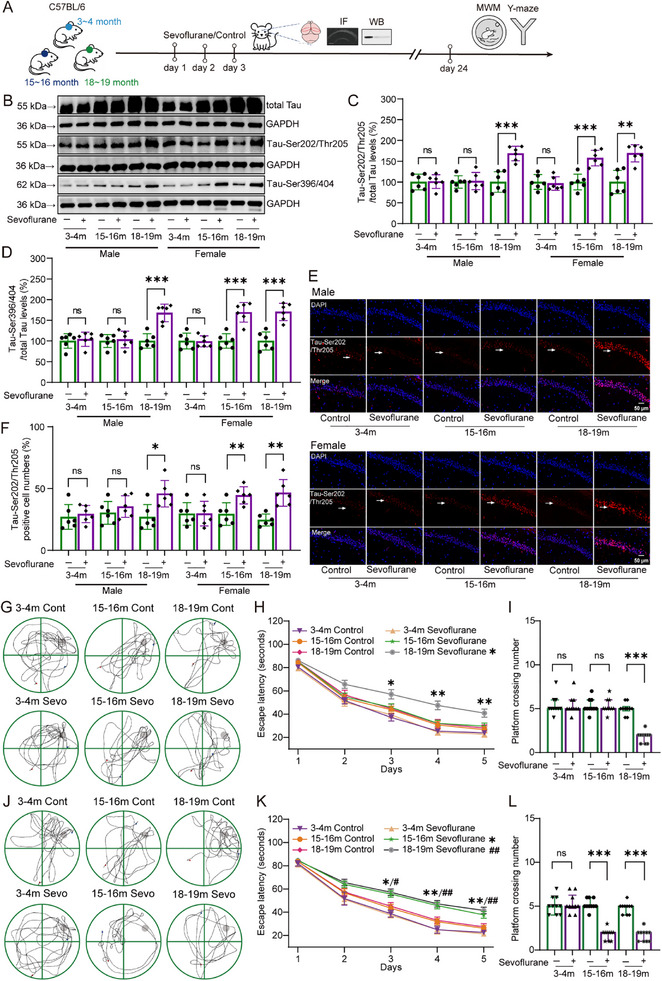
Effects of sevoflurane on Tau phosphorylation and cognitive function in the hippocampus of male and female mice across different age groups. A) Timeline of the experimental design for this part of the study. B–D) Representative Western blot images and quantification of Tau‐Ser202/Thr205 and Tau‐Ser396/404 in the hippocampus of male and female mice across different age groups (*n* = 6 per group). E,F) Representative immunofluorescence images and quantification of Tau‐Ser202/Thr205 in the CA1 region of the hippocampus of male and female mice across different age groups (indicated by white arrows, n = 6 per group). G–I) Representative tracking trajectories, latency to locate the hidden platform, and number of platform crossings in the Morris water maze task for male mice at different ages (*n* = 10 per group). J–L) Representative tracking trajectories, latency to locate the hidden platform, and number of platform crossings in the Morris water maze task for female mice at different ages (*n* = 10 per group). Data in (C), (D), and (F) are presented as mean ± SD; data in (H) and (K) are presented as mean ± SEM; data in (I) and (L) are presented as median and IQR. Statistical analysis: Unpaired *t*‐test was used for (C), (D), and (F); Two‐way repeated measures ANOVA was applied to (H) and (K); Mann‐Whitney U test was used for (I) and (L). ns, no significance; *p < 0.05; **p < 0.01; ***p < 0.001; ^#^ *p *< 0.05; ^##^ *p *< 0.01.

To investigate the sex‐ and age‐dependent neurotoxic effects of sevoflurane, we first assessed Tau phosphorylation and cognitive function in the hippocampus of mice, as outlined in the experimental design shown in **Figure** **1**A. Western blot results showed that, in adult mice, sevoflurane anesthesia had no significant effect on the expression levels of Tau‐Ser202/Thr205 (Figure 1B and C; Males: 100.00 ± 18.73 vs 101.12 ± 16.55, Unpaired *t*‐test, t(10) = −0.11, *p =* 0.915; Females: 100.00 ± 17.91 vs 96.36 ± 16.23, Unpaired *t*‐test, t(10) = 0.37, *p =* 0.720) or Tau‐Ser396/404 (Figure 1B,D; Males: 100.00 ± 17.51 vs 104.15 ± 17.08, Unpaired *t*‐test, t(10) = −0.42, *p =* 0.687; Females: 100.00 ± 19.08 vs 98.89 ± 13.51, Unpaired *t*‐test, t(10) = 0.17, *p =* 0.910), regardless of sex. In aged mice, sevoflurane significantly increased the expression of both Tau‐Ser202/Thr205 (Males: 100.00 ± 24.72 vs 168.52 ± 17.47, Unpaired *t*‐test, t(10) = −5.55, *p* < 0.001; Females: 100.00 ± 28.02 vs 169.10 ± 20.90, Unpaired *t*‐test, t(10) = −4.84, *p =* 0.001) and Tau‐Ser396/404 (Males: 100.00 ± 17.58 vs 167.75 ± 21.31, Unpaired *t*‐test, t(10) = −6.01, *p* < 0.001; Females: 100.00 ± 21.77 vs 170.21 ± 20.92, Unpaired *t*‐test, t(10) = −5.70, *p* < 0.001), independent of sex. Notably, in middle‐aged mice, sevoflurane significantly upregulated Tau‐Ser202/Thr205 (100.00 ± 18.80 vs 157.62 ± 18.91, Unpaired *t*‐test, t(10) = −5.29, *p* < 0.001) and Tau‐Ser396/404 (100.00 ± 17.46 vs 169.31 ± 23.62, Unpaired *t*‐test, t(10) = −5.78, *p* < 0.001) expression in females but not in males.

To further confirm these findings, we performed immunofluorescence staining of hippocampal sections. Consistent with the Western blot data, sevoflurane did not alter Tau‐Ser202/Thr205 levels in the dCA1 region of adult mice of either sex (Figure 1E,F; Males: 27.00 ± 10.04 vs 29.33 ± 6.95, Unpaired *t*‐test, t(10) = −0.47, *p =* 0.65; Females: 29.50 ± 9.09 vs 29.67 ± 9.97, Unpaired *t*‐test, t(10) = −0.03, *p =* 0.976). In contrast, Tau‐Ser202/Thr205 expression was significantly elevated in the dCA1 region of aged mice following sevoflurane anesthesia, regardless of sex (Males: 27.00 ± 10.08 vs 45.83 ± 10.72, Unpaired *t*‐test, t(10) = −3.14, *p =* 0.011; Females: 24.50 ± 4.85 vs 46.50 ± 10.78, Unpaired *t*‐test, t(10) = −4.56, *p =* 0.001). In middle‐aged mice, increased Tau‐Ser202/Thr205 expression was observed only in females (29.33 ± 9.16 vs 44.50 ± 6.98, Unpaired *t*‐test, t(10) = −3.23, *p =* 0.009).

Results from the Morris water maze assessment of cognitive performance showed that, in adult mice, sevoflurane had no significant impact on escape latency (Figure 1H,K; Males: 43.72 ± 1.34 vs 43.19 ± 1.34, Repeated measures ANOVA, F(1,18) = 0.08, *p =* 0.783; Females: 44.02 ± 1.84 vs 43.12 ± 1.84, Repeated measures ANOVA, F(1,18) = 0.12, *p =* 0.732) or platform crossings (Figure 1I,L; Males: Mdn = 5.00, IQR = [5.00–6.00] vs Mdn = 5.00, IQR = [5.0–6.25], Mann‐Whitney U test, U = 45.00, *p =* 0.677; Females: Mdn = 5.00, IQR = [4.00–6.00] vs Mdn = 5.00, IQR = [4.75–6.25], Mann‐Whitney U test, U = 45.00, *p =* 0.688). However, in aged mice, sevoflurane significantly prolonged escape latency (Males: 49.25 ± 2.93 vs 59.46 ± 2.93, Repeated measures ANOVA, F(1,18) = 6.08, *p =* 0.024; Females: 49.34 ± 2.34 vss 59.18 ± 2.34, Repeated measures ANOVA, F(1,18) = 8.83, *p =* 0.008) and reduced the number of platform crossings (Males: Mdn = 5.00, IQR = [4.75–5.25] vs Mdn = 2.00, IQR = [1.00–2.00], Mann‐Whitney U test, U = 0.00, *p* < 0.001; Females: Mdn = 5.00, IQR = [4.00–5.00] vs Mdn = 2.00, IQR = [1.00–2.00], Mann‐Whitney U test, U = 0.00, *p* < 0.001). In middle‐aged mice, these impairments were observed exclusively in females (Latency: 48.37 ± 2.37 vs 57.61 ± 2.37, Repeated measures ANOVA, F(1,18) = 7.63, *p =* 0.013; Crossings: Mdn = 5.00, IQR = [5.00–6.00] vs Mdn = 2.00, IQR = [1.00–2.00], Mann‐Whitney U test, U = 0.00, *p* < 0.001). Consistent results were obtained in the Y‐maze test. In adult mice, sevoflurane did not alter exploration time (Figure S1A–C, Supporting Information; Males: 51.73 ± 11.41 vs 51.20 ± 9.05, Unpaired *t*‐test, t(18) = 0.11, *p =* 0.911; Females: 51.18 ± 8.83 vs 51.94 ± 7.17, Unpaired *t*‐test, t(18) = −0.21, *p =* 0.835) or novel arm entries (Figure S1D, Supporting Information; Males: 5.40 ± 0.97 vs 5.30 ± 0.95, Unpaired *t*‐test, t(18) = 0.23, *p =* 0.818; Females: 5.20 ± 0.92 vs 5.40 ± 0.84, Unpaired *t*‐test, t(18) = −0.51, *p =* 0.618). In aged mice, sevoflurane exposure significantly decreased both exploration time (Males: 51.28 ± 8.36 vs 37.61 ± 9.39, Unpaired *t*‐test, t(18) = 3.44, *p =* 0.003; Females: 49.96 ± 8.36 vs 38.47 ± 8.88, Unpaired *t*‐test, t(18) = 2.98, *p =* 0.008) and novel arm entries (Males: 5.10 ± 0.99 vs 3.80 ± 1.03, t(18) = 2.87, *p =* 0.01; Females: 5.10 ± 0.99 vs 3.70 ± 0.95, Unpaired *t*‐test, t(18) = 3.22, *p =* 0.005), with no sex‐specific differences. In middle‐aged mice, these deficits were again observed only in females (Exploration time: 52.06 ± 9.23 vs 36.82 ± 9.11, Unpaired *t*‐test, t(18) = 3.72, *p =* 0.002; Number of entries: 5.10 ± 0.99 vs 3.80 ± 1.03, Unpaired *t*‐test, t(18) = 2.87, *p =* 0.01).

### 2.2 Estrogen Selectively Alleviated Hippocampal Tau Phosphorylation and Cognitive Deficits Only in Middle‐Aged Mice, but not in Aged Mice

**Figure 2:**
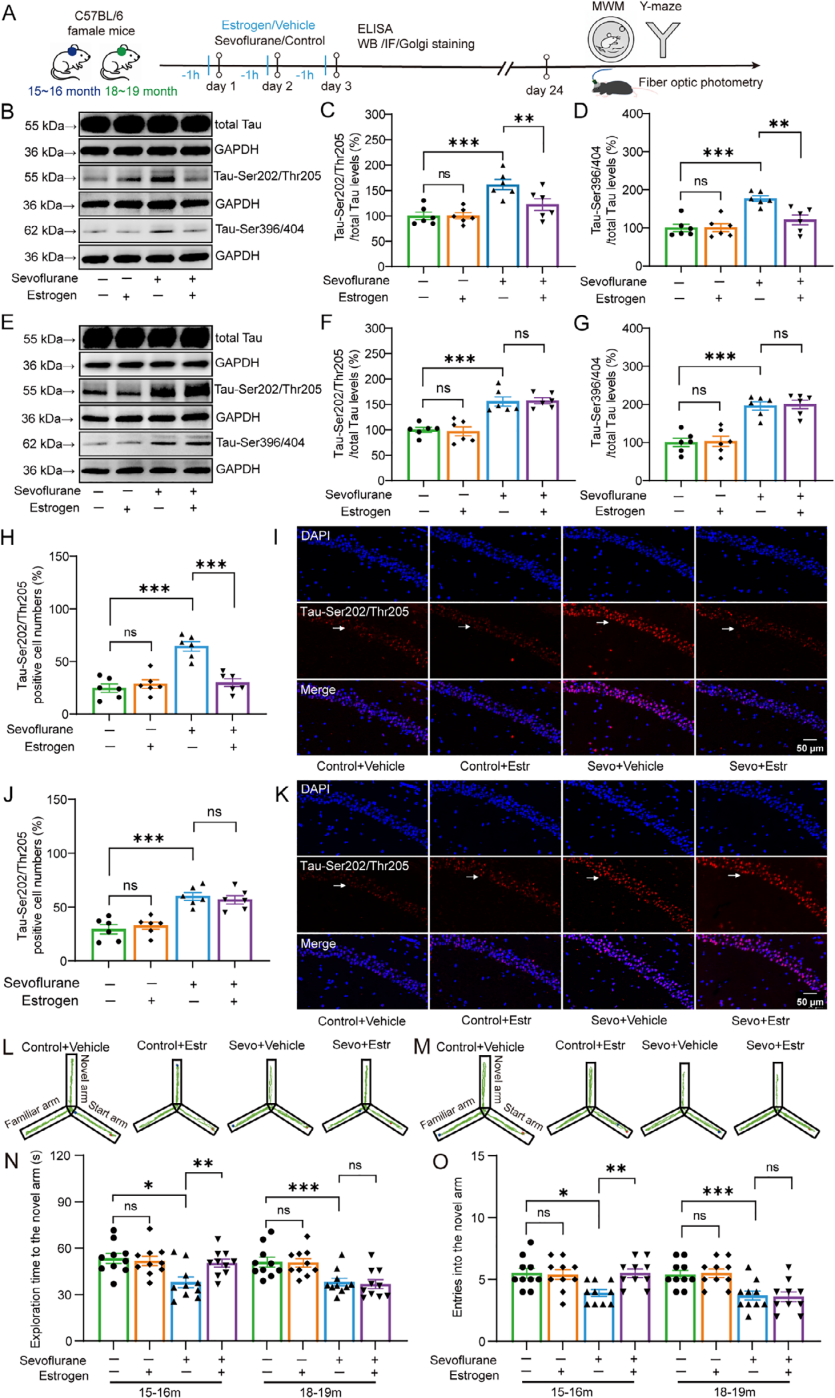
Effects of estrogen and sevoflurane on Tau phosphorylation and cognitive function in the hippocampus of middle‐aged and aged female mice. A) Timeline of the experimental design for this part of the study. B–D) Representative Western blot images and quantification of Tau‐Ser202/Thr205 and Tau‐Ser396/404 in the hippocampus of middle‐aged female mice (*n* = 6 per group). E–G) Representative Western blot images and quantification of Tau‐Ser202/Thr205 and Tau‐Ser396/404 in the hippocampus of aged female mice (*n* = 6 per group). H,I) Representative immunofluorescence images and quantification of Tau‐Ser202/Thr205 in the CA1 region of the hippocampus in middle‐aged female mice (indicated by white arrows, n = 6 per group). J,K) Representative immunofluorescence images and quantification of Tau‐Ser202/Thr205 in the CA1 region of the hippocampus in aged female mice (indicated by white arrows, *n* = 6 per group). L,M) Representative tracking trajectories in the Y‐maze task for middle‐aged and aged female mice. N,O) Time spent exploring the novel arm and number of entries into the novel arm in the Y‐maze task for middle‐aged and aged female mice (*n* = 10 per group). Data in (C), (D), (F), (G), (H), (J), (N), and (O) are presented as mean ± SEM. Statistical analysis: Two‐way ANOVA with Bonferroni post hoc test was used for (C), (D), (F), (G), (H), (J), (N), and (O). Abbreviations: Estrogen (Estr); Sevoflurane (Sevo). ns, no significance; *p < 0.05; **p < 0.01; ***p < 0.001.

To evaluate the impact of estrogen levels on sevoflurane‐induced neurotoxicity, we first measured hippocampal estrogen concentrations in female mice of different ages using a highly sensitive ELISA kit. The overall experimental workflow is illustrated in **Figure** **2**A. We found that hippocampal estrogen levels in mice gradually declined with age (Figure S2A, 100.00 ± 6.00 vs 54.53 ± 4.59 vs 20.54 ± 4.02; One‐way ANOVA, F(2,15) = 65.14, *p* < 0.001, Supporting Information). Estrogen supplementation significantly increased hippocampal estrogen levels in both middle‐aged and aged female mice (Figure S2B, 61.36 ± 10.54 vs 212.77 ± 10.54; Two‐way ANOVA, F(1,20) = 103.27, *p* < 0.001, Supporting Information).

To investigate the effect of estrogen supplementation on sevoflurane‐induced neurotoxicity, we first evaluated Tau phosphorylation in the hippocampus of mice.Western blot analysis revealed that sevoflurane increased the expression of Tau‐Ser202/Thr205 (Middle‐aged: Figure 2B,C; 100.16 ± 6.39 vs 141.87 ± 6.39, Two‐way ANOVA, F(1,20) = 21.33, *p* < 0.001; Aged: Figure 2E,F; 98.43 ± 5.30 vs 156.27 ± 5.30, Two‐way ANOVA, F(1,20) = 59.75, *p* < 0.001) and Tau‐Ser396/404 (Middle‐aged: Figure 2B,D; 100.22 ± 7.23 vs 148.94 ± 7.23, Two‐way ANOVA, F(1,20) = 22.70, *p* < 0.001; Aged: Figure 2E,G; 101.43 ± 8.31 vs 197.88 ± 8.31, Two‐way ANOVA, F(1,20) = 67.29, *pp* < 0.001) in the mouse hippocampus. Estrogen had no effect on hippocampal Tau‐Ser202/Thr205 expression (Middle‐aged: 100.00 ± 9.03 vs 100.32 ± 9.03, Two‐way ANOVA with Bonferroni post hoc test, t(20) = 0.001, *p* = 0.98; Aged: 100.00 ± 7.48 vs 96.861 ± 7.48, Two‐way ANOVA with Bonferroni post hoc test, t(20) = 0.09, *p* = 0.77) or on Tau‐Ser396/404 expression (Middle‐aged: 100.00 ± 10.23 vs 100.44 ± 10.23, Two‐way ANOVA with Bonferroni post hoc test, t(20) = 0.001, *p* = 0.976; Aged: 100.00 ± 11.76 vs 102.86 ± 11.76, Two‐way ANOVA with Bonferroni post hoc test, t(20) = 0.03, *p* = 0.865) in control mice. However, estrogen significantly reduced the expression of Tau‐Ser202/Thr205 (161.53 ± 9.03 vs 122.22 ± 9.03, Two‐way ANOVA with Bonferroni post hoc test, t(20) = 9.47, *p* = 0.006) and Tau‐Ser396/404 (176.76 ± 10.23 vs 121.11 ± 10.23, Two‐way ANOVA with Bonferroni post hoc test, t(20) = 14.81, *p* = 0.001) in the hippocampus of middle‐aged mice exposed to sevoflurane, but had no effect in aged mice exposed to sevoflurane (Tau‐Ser202/Thr205: 155.57 ± 7.48 vs 156.97 ± 7.48, Two‐way ANOVA with Bonferroni post hoc test, t(20) = 0.02, *p* = 0.897; Tau‐Ser396/404: 195.89 ± 11.76 vs 199.87 ± 11.76, Two‐way ANOVA with Bonferroni post hoc test, t(20) = 0.06, *p* = 0.813).

Immunofluorescence staining similarly showed that sevoflurane increased Tau‐Ser202/Thr205 expression in the dCA1 region of the mouse hippocampus (Middle‐aged: Figure 2H–K, 25.58 ± 2.86 vs 47.08 ± 2.86, Two‐way ANOVA, F(1,20) = 25.61, *p* < 0.001; Aged: Figure 2J,K, 31.00 ± 2.68 vs 58.25 ± 2.68, Two‐way ANOVA, F(1,20) = 51.54, *p* < 0.001). Estrogen had no effect on Tau‐Ser202/Thr205 expression in control group (Middle‐aged: 24.67 ± 4.05 vs 28.50 ± 4.05, Two‐way ANOVA with Bonferroni post hoc test, t(20) = 0.45, *p* = 0.511; Aged: 29.33 ± 3.80 vs 32.67 ± 3.80, Two‐way ANOVA with Bonferroni post hoc test, t(20) = 0.39, *p* = 0.542). However, estrogen significantly reduced Tau‐Ser202/Thr205 expression in the dCA1 region of middle‐aged mice exposed to sevoflurane (64.33 ± 4.05 vs 29.83 ± 4.05, Two‐way ANOVA with Bonferroni post hoc test, t(20) = 36.27, *p* < 0.001), but had no effect in aged mice exposed to sevoflurane (59.83 ± 3.80 vs 56.67 ± 3.80, Two‐way ANOVA with Bonferroni post hoc test, t(20) = 0.35, *p* = 0.562).

Behavioral results from the Morris water maze showed that sevoflurane increased the latency to find the hidden platform (Middle‐aged: Figure S3A,B, 48.20 ± 1.56 vs 59.29 ± 1.56, Repeated measures ANOVA, F(1,18) = 24.10, *p* < 0.001; Aged: Figure S3D,E, 49.89 ± 2.27 vs 60.92 ± 2.27, Repeated measures ANOVA, F(1,18) = 11.84, *p =* 0.003, Supporting Information) and reduced the number of platform crossings (Middle‐aged: Figure S3A,C, Mdn = 5.00, IQR = [4.00–6.25] vs Mdn = 2.00, IQR = [1.75–2.25], Kruskal‐Wallis test with Dunn's post hoc, Z = 3.86, *p* < 0.001; Aged: Figure S3D,F, Mdn = 5.00, IQR = [4.00–5.25] vs Mdn = 2.00, IQR = [1.75–2.25], Kruskal‐Wallis test with Dunn's post hoc, Z = 3.75, *p* = 0.001, Supporting Information) in mice. Estrogen had no affect on the latency to find the platform (Middle‐aged: 48.20 ± 1.80 vs 48.27 ± 1.80, Repeated measures ANOVA, F(1,18) = 0.001, *p =* 0.979; Aged: 49.89 ± 2.02 vs 49.27 ± 2.02, Repeated measures ANOVA, F(1,18) = 0.05, *p =* 0.83) or the number of platform crossings (Middle‐aged: Mdn = 5.00, IQR = [4.00–6.25] vs Mdn = 5.00, IQR = [4.75–5.50], Kruskal‐Wallis test with Dunn's post hoc, Z = 0.05, *p* = 1; Aged: Mdn = 5.00, IQR = [4.00–5.25] vs Mdn = 5.00, IQR = [4.00–5.50], Kruskal‐Wallis test with Dunn's post hoc, Z = 0.16, *p* = 1) in control mice. However, in middle‐aged mice exposed to sevoflurane, estrogen significantly reduced the latency to find the platform (59.29 ± 1.80 vs 47.25 ± 1.80, Repeated measures ANOVA, F(1,18) = 22.37, *p* < 0.001) and increased the number of platform crossings (Mdn = 2.00, IQR = [1.75–2.25] vs Mdn = 5.00, IQR = [4.75–6.25], Kruskal‐Wallis test with Dunn's post hoc, Z = 4.08, *p* < 0.001), but had no effect in aged mice exposed to sevoflurane (latency: 60.92 ± 2.18 vs 58.95 ± 2.18, Repeated measures ANOVA, F(1,18) = 0.41, *p =* 0.529; platform crossings: (Mdn = 2.00, IQR = [1.75–2.25] vs Mdn = 2.00, IQR = [1.00–2.25], Kruskal‐Wallis test with Dunn's post hoc, Z = 0.16, *p* = 1). Similarly, in the Y‐maze test, sevoflurane significantly reduced both the exploration time in the novel arm (Middle‐aged: Figure 2L,N, 52.56 ± 2.18 vs 44.13 ± 2.18, Two‐way ANOVA, F(1,36) = 7.48, *p* = 0.01; Aged: Figure 2M,N, 50.76 ± 2.02 vs 37.39 ± 2.02, Two‐way ANOVA, F(1,36) = 22.01, *p* < 0.001) and the number of entries into the novel arm (Figure 2O, 5.45 ± 0.25 vs 4.70 ± 0.25, Two‐way ANOVA, F(1,36) = 4.37, *p* = 0.044; Aged: 5.45 ± 0.26 vs 3.65 ± 0.26, Two‐way ANOVA, F(1,36) = 24.61, *p* < 0.001). Estrogen had no affect on the time spent exploring (Middle‐aged: 53.40 ± 3.08 vs 51.72 ± 3.08, Two‐way ANOVA with Bonferroni post hoc test, t(36) = 0.148, *p* = 0.70; Aged: 51.02 ± 2.85 vs 50.51 ± 2.85, Two‐way ANOVA with Bonferroni post hoc test, t(36) = 0.02, *p* = 0.901) or the number of entries into the novel arm (Middle‐aged: 5.50 ± 0.36 vs 5.40 ± 0.36, Two‐way ANOVA with Bonferroni post hoc test, t(36) = 0.04, *p* = 0.845; Aged: 5.40 ± 0.36 vs 5.50 ± 0.36, Two‐way ANOVA with Bonferroni post hoc test, t(36) = 0.04, *p* = 0.847) in control mice. However, it significantly increased both the time spent in the novel arm (37.93 ± 3.08 vs 50.33 ± 3.08, Two‐way ANOVA with Bonferroni post hoc test, t(36) = 8.09, *p* = 0.007) and the number of entries (3.90 ± 0.360 vs 5.50 ± 0.360, Two‐way ANOVA with Bonferroni post hoc test, t(36) = 9.95, *p* = 0.003) in middle‐aged mice exposed to sevoflurane, but had no effect in aged mice exposed to sevoflurane (Exploration time: 38.05 ± 2.85 vs 36.74 ± 2.85, Two‐way ANOVA with Bonferroni post hoc test, t(36) = 0.11, *p* = 0.746; Number of entries: 3.70 ± 0.36 vs 3.60 ± 0.36, Two‐way ANOVA with Bonferroni post hoc test, t(36) = 0.04, *p* = 0.847).

In addition, we examined the expression of the synaptic marker PSD95. We found that sevoflurane exposure significantly reduced PSD95 levels in the hippocampus of mice (Middle‐aged: Figure S3G,H, 86.82 ± 3.13 vs 98.79 ± 3.13, Two‐way ANOVA, F(1,20) = 7.34, *p* = 0.013; Aged: Figure S3I,J, 81.76 ± 2.71 vs 82.78 ± 2.71, Two‐way ANOVA, F(1,20) = 0.07, *p* = 0.793, Supporting Information). Estrogen treatment had no effect on PSD95 expression in control mice (Middle‐aged: 100.00 ± 4.42 vs 100.09 ± 4.42, Two‐way ANOVA with Bonferroni post hoc test, t(20) = 0.00, *p* = 0.988; Aged: 100.00 ± 3.83 vs 100.60 ± 3.83, Two‐way ANOVA with Bonferroni post hoc test, t(20) = 0.012, *p* = 0.912); however, it significantly increased PSD95 expression in the hippocampus of middle‐aged mice exposed to sevoflurane (73.63 ± 4.42 vs 97.49 ± 4.42, Two‐way ANOVA with Bonferroni post hoc test, t(20) = 14.57, *p* = 0.001). Notably, this effect was not observed in aged mice exposed to sevoflurane (63.52 ± 3.83 vs 64.95 ± 3.83, Two‐way ANOVA with Bonferroni post hoc test, t(20) = 0.07, *p* = 0.794).

### 2.3 Estrogen Selectively Enhanced Neuronal Ca^2^⁺ Signaling Fluctuations and Firing Activity, and Improved Dendritic Morphology in the dCA1 Region of Middle‐Aged Mice, but not in Aged Mice

**Figure 3:**
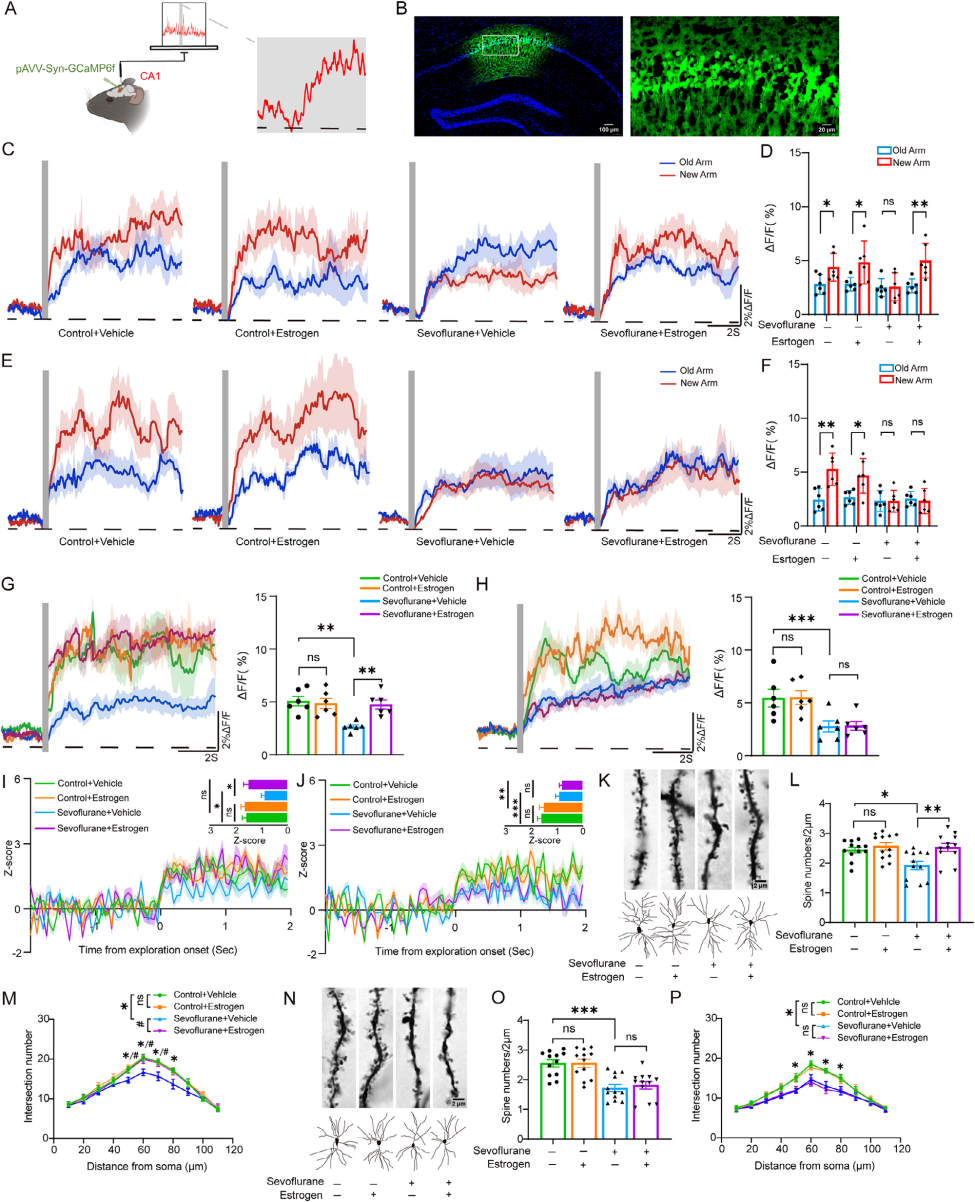
Effects of estrogen and sevoflurane on spontaneous Ca^2^⁺ activity, neuronal firing, and dendritic spine morphology in dCA1 neurons of middle‐aged and aged female mice. A,B) Schematic illustration of fiber photometry recording of spontaneous calcium signals (A) and confirmation of pAAV‐Syn‐GCaMP6f viral injection site (B). C,D) Representative traces and quantification of spontaneous Ca^2^⁺ signal fluctuations in middle‐aged female mice during exploration of the novel versus familiar arms in the Y‐maze (*n* = 6 per group). E,F) Representative traces and quantification of spontaneous Ca^2^⁺ signal fluctuations in aged female mice during exploration of the novel versus familiar arms in the Y‐maze (*n* = 6 per group). G,H) Transient Ca^2^⁺ signal fluctuations upon entry into the novel arm in middle‐aged (G) and aged (H) female mice (left: representative trace; right: quantification; *n* = 6 per group). I) Visualization and quantification of neuronal firing frequency in middle‐aged female mice during exploration of a novel object (14 cells from *n* = 6 mice per group). J) Visualization and quantification of neuronal firing frequency in aged female mice during exploration of a novel object (14 cells from *n* = 6 mice per group). K) Representative images of dendritic spines (top) and dendritic shafts (bottom) in dCA1 neurons of middle‐aged female mice. L,M) Quantification of dendritic spine density (L) and dendritic shaft number (M) in dCA1 neurons of middle‐aged female mice (*n* = 6 per group). N) Representative images of dendritic spines (top) and dendritic shafts (bottom) in dCA1 neurons of aged female mice. O,P) Quantification of dendritic spine density (O) and dendritic shaft number (P) in dCA1 neurons of aged female mice (*n* = 6 per group). Data in (D) and (F) are presented as mean ± SD; data in (G), (H), (I), (J), (L), (M), (O), and (P) are presented as mean ± SEM. Statistical analysis: Unpaired *t*‐test was used for (D) and (F); Two‐way ANOVA with Bonferroni post hoc test was applied to (G), (H), (I), (J), (L), and (O); Repeated measures ANOVA was applied to (M) and (P). ns, no significance; *P<0.05; **p < 0.01; ***p < 0.001; ^#^ *p *< 0.05.

To examine the effects of estrogen on hippocampal neuronal activity in mice, we used fiber photometry to measure the amplitude of Ca^2^⁺ signals in the hippocampus. We first confirmed that the viral injection sites were accurately localized to the dCA1 region (**Figure** **3**A,B). The results showed that in the control group, both middle‐aged (Figure 3C,D, 2.80 ± 0.91 vs 4.38 ± 1.30, Unpaired *t*‐test, t(10) = 2.45, *p =* 0.034) and aged mice (Figure 3E,F, 2.44 ± 1.03 vs 5.27 ± 1.51, Unpaired *t*‐test, t(10) = −3.80, *p =* 0.003) exhibited differences in Ca^2^⁺ signal amplitude between explorations of the novel and familiar arms. Sevoflurane abolished this difference (Middle‐aged: 2.49 ± 0.83 vs 2.57 ± 1.30, Unpaired *t*‐test, t(10) = −0.12, *p =* 0.907; Aged: 2.31 ± 0.98 vs 2.30 ± 1.01, Unpaired *t*‐test, t(10) = 0.02, *p =* 0.985). Estrogen treatment restored the difference in Ca^2^⁺ signal fluctuations between the novel and familiar arms in middle‐aged mice exposed to sevoflurane (2.65 ± 0.65 vs 4.98 ± 1.60, Unpaired *t*‐test, t(10) = 3.32, *p =* 0.008), but had no effect in aged mice exposed to sevoflurane (2.53 ± 0.68 vs 2.32 ± 1.17, Unpaired *t*‐test, t(10) = 0.37, *p =* 0.719). Additionally, sevoflurane significantly reduced the amplitude of transient Ca^2^⁺ signals upon entry into the novel arm (Middle‐aged: Figure 3G, 4.96 ± 0.29 vs 3.70 ± 0.29, Two‐way ANOVA, F(1,20) = 9.61, *p* = 0.006; Aged: Figure 3H, 5.48 ± 0.44 vs 2.80 ± 0.44, Two‐way ANOVA, F(1,20) = 18.74, *p* < 0.001). Estrogen had no effect in control mice (Middle‐aged: 5.07 ± 0.41 vs 4.85 ± 0.41, Two‐way ANOVA with Bonferroni post hoc test, t(20) = 0.14, *p* = 0.708; Aged: 5.46 ± 0.62 vs 5.50 ± 0.62, Two‐way ANOVA with Bonferroni post hoc test, t(20) = 0.002, *p* = 0.965), and rescued the amplitude of transient Ca^2^⁺ signals in the sevoflurane‐treated middle‐aged mice upon entry into the novel arm (2.65 ± 0.41 vs 4.75 ± 0.41, Two‐way ANOVA with Bonferroni post hoc test, t(20) = 13.42, *p* = 0.002), but had no effect in aged mice exposed to sevoflurane (2.77 ± 0.62 vs 2.83 ± 0.62, Two‐way ANOVA with Bonferroni post hoc test, t(20) = 0.01, *p* = 0.94).

To further investigate the effect of estrogen on neuronal firing frequency in the hippocampus, we performed in vivo electrophysiological recordings. The results showed that, compared with the control group, sevoflurane exposure significantly reduced the neuronal firing rate in the dCA1 region of the hippocampus during novel object exploration (time 0 indicates the onset of exploratory behavior) in both middle‐aged and aged mice (Middle‐aged: Figure 3I; Figure S4A, 1.61 ± 0.12 vs 1.19 ± 0.12, Two‐way ANOVA, F(1,52) = 6.70, *p* = 0.012, Supporting Information; Aged: Figure 3J; Figure S4B, 1.54 ± 0.11 vs 0.84 ± 0.11, Two‐way ANOVA, F(1,52) = 21.61, *p* < 0.001, Supporting Information). Estrogen supplementation did not affect neuronal firing frequency during exploration in control mice (Middle‐aged: 1.58 ± 0.17 vs 1.64 ± 0.17, Two‐way ANOVA with Bonferroni post hoc test, t(52) = 0.06, *p* = 0.805; Aged: 1.59 ± 0.15 vs 1.50 ± 0.15, Two‐way ANOVA with Bonferroni post hoc test, t(52) = 0.20, *p* = 0.661), but it significantly restored the reduced firing frequency in sevoflurane‐exposed middle‐aged mice (0.88 ± 0.17 vs 1.50 ± 0.17, Two‐way ANOVA with Bonferroni post hoc test, t(52) = 7.11, *p* = 0.01), however, this effect was not observed in aged mice (0.90 ± 0.15 vs 0.79 ± 0.15, Two‐way ANOVA with Bonferroni post hoc test, t(52) = 0.24, *p* = 0.626).

Golgi staining further showed that sevoflurane markedly decreased dendritic spine density (Middle‐aged: Figure 3K–M, 2.50 ± 0.09 vs 2.23 ± 0.09, Two‐way ANOVA, F(1,44) = 5.03, *p* = 0.03; Aged: Figure 3N–P, 2.55 ± 0.09 vs 1.77 ± 0.09, Two‐way ANOVA, F(1,44) = 37.17, *p* < 0.001) and dendritic branching in the dCA1 region of both middle‐aged and aged mice (Middle‐aged: 13.78 ± 0.43 vs 12.08 ± 0.43, Repeated measures ANOVA, F(1,10) = 7.97, *p =* 0.018; Aged: 12.15 ± 0.61 vs 10.20 ± 0.61, Repeated measures ANOVA, F(1,10) = 5.16, *p =* 0.046). Estrogen did not alter dendritic spine density (Middle‐aged: 2.44 ± 0.12 vs 2.57 ± 0.12, Two‐way ANOVA with Bonferroni post hoc test, t(44) = 0.50, *p* = 0.485; Aged: 2.55 ± 0.13 vs 1.81 ± 0.13, Two‐way ANOVA with Bonferroni post hoc test, t(44) < 0.001, *p* = 0.987) or the number of dendritic branches (Middle‐aged: 13.78 ± 0.34 vs 14.02 ± 0.34, Repeated measures ANOVA, F(1,10) = 0.25, *p =* 0.625; Aged: 12.15 ± 0.50 vs 12.05 ± 0.50, Repeated measures ANOVA, F(1,10) = 0.02, *p =* 0.884) in control mice. However, in middle‐aged mice exposed to sevoflurane, it effectively restored both dendritic spine density (1.92 ± 0.12 vs 2.53 ± 0.12, Two‐way ANOVA with Bonferroni post hoc test, t(44) = 12.08, *p* = 0.001) and dendritic branching (12.08 ± 0.45 vs 13.65 ± 0.45, Repeated measures ANOVA, F(1,10) = 6.02, *p =* 0.034). In contrast, no such effects were observed in aged mice treated with sevoflurane (Spine density: 1.72 ± 0.13 vs 1.81 ± 0.13, Two‐way ANOVA with Bonferroni post hoc test, t(44) = 0.27, *p* = 0.605; Branching: 10.20 ± 0.65 vs 10.17 ± 0.65, Repeated measures ANOVA, F(1,10) = 0.001, *p =* 0.974). We also quantified the number of mushroom‐type dendritic spines and found that sevoflurane significantly reduced their density (Middle‐aged: Figure S4C, 0.41 ± 0.02 vs 0.35 ± 0.02, Two‐way ANOVA, F(1,44) = 4.43, *p* = 0.04; Aged: Figure S4D, 0.40 ± 0.02 vs 0.28 ± 0.02, Two‐way ANOVA, F(1,44) = 17.835, *p* < 0.001, Supporting Information). Estrogen treatment did not alter mushroom spine density in control mice (Middle‐aged: 0.40 ± 0.02 vs 0.42 ± 0.02, Two‐way ANOVA with Bonferroni post hoc test, t(44) = 0.35, *p* = 0.56; Aged: 0.40 ± 0.03 vs 0.40 ± 0.03, t(44) = 0.006, *p* = 0.937). However, in middle‐aged mice exposed to sevoflurane, estrogen effectively restored mushroom spine density (0.30 ± 0.02 vs 0.41 ± 0.02, t(44) = 10.10, *p* = 0.003). In contrast, no such effect was observed in aged mice exposed to sevoflurane (0.27 ± 0.03 vs 0.28 ± 0.03, t(44) = 0.07, *p* = 0.800).

### 2.4 ERα Antagonist Blocked the Protective Effects of Estrogen on Tau Phosphorylation, Cognitive Function, and Dendritic Morphology in the dCA1 Region in Sevoflurane‐Exposed Mice

**Figure 4:**
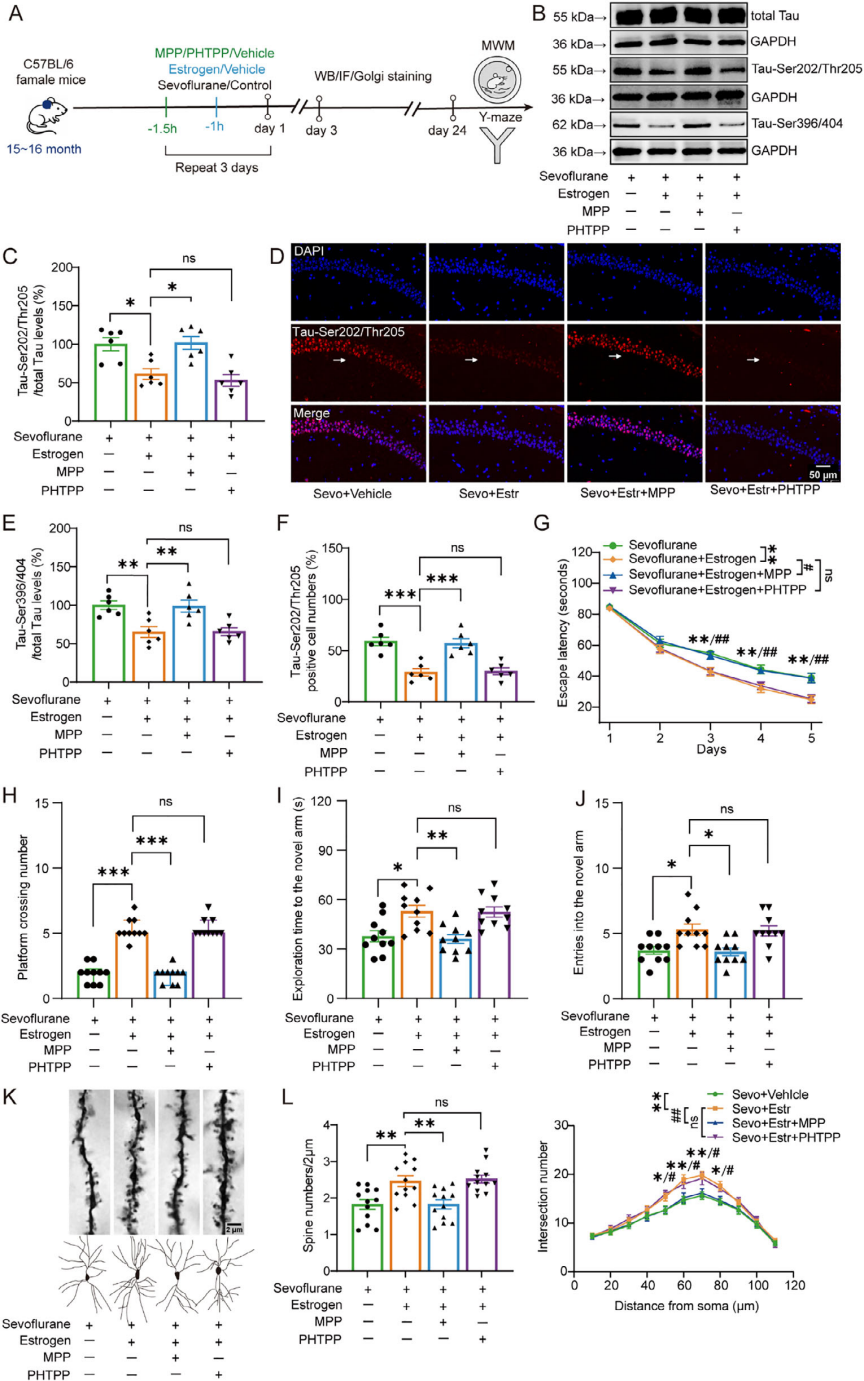
Effects of estrogen combined with receptor antagonists and sevoflurane on Tau phosphorylation, cognitive function, and dendritic spine morphology in dCA1 neurons of middle‐aged female mice. A) Timeline of the experimental design for this part of the study. B,C,E) Representative Western blot images and quantification of Tau‐Ser202/Thr205 and Tau‐Ser396/404 in the hippocampus of middle‐aged female mice (*n* = 6 per group). D,F) Representative immunofluorescence images and quantification of Tau‐Ser202/Thr205 in the CA1 region of the hippocampus in middle‐aged female mice (indicated by white arrows, *n* = 6 per group). G,H) Latency to locate the hidden platform and number of platform crossings in the Morris water maze task for middle‐aged female mice (*n* = 10 per group). I J) Time spent exploring the novel arm and number of entries into the novel arm in the Y‐maze task for middle‐aged female mice (*n* = 10 per group). K) Representative images of dendritic spines (top) and dendritic shafts (bottom) in dCA1 neurons of middle‐aged female mice. L) Quantification of dendritic spine density (left) and dendritic shaft number (right) in dCA1 neurons of middle‐aged female mice (*n* = 6 per group). Data in (C), (E), (F), (G), (I), (J), and (L) are presented as mean ± SEM; data in (H) is presented as median and IQR. Statistical analysis: One‐way ANOVA with Bonferroni post hoc test was used for (C), (E), (F), (I), (J), and (L, left); Repeated measures ANOVA was applied to (G) and (L, right); Kruskal‐Wallis test with Dunn's post hoc was used for (H). Abbreviations: Estrogen (Estr); Sevoflurane (Sevo). ns, no significance; *p < 0.05; **p < 0.01; ***p < 0.001; ^#^ *p *< 0.05; ^# #^ *p *< 0.01.

Since estrogen primarily exerts its effects through ERα and ERβ, we used MPP and PHTPP to selectively block ERα and ERβ, respectively, to determine which receptor mediates its neuroprotective effects, as outlined in the experimental procedure shown in **Figure** **4**A. We first used Western blot to assess Tau phosphorylation levels in the hippocampus. Compared to the sevoflurane group, estrogen significantly reduced hippocampal Tau‐Ser202/Thr205 (Figure 4B,C, 100.00 ± 8.54 vs 61.22 ± 7.02, One‐way ANOVA with Bonferroni post hoc test, t(20) = 3.46, *p* = 0.015) and Tau‐Ser396/404 (Figure 4B,E, 100.00 ± 5.57 vs 65.09 ± 7.09, One‐way ANOVA with Bonferroni post hoc test, t(20) = 3.84, *p* = 0.006) expression. However, co‐administration of MPP blocked this estrogen‐mediated reduction (Tau‐Ser202/Thr205: 61.22 ± 7.02 vs 101.63 ± 8.42, One‐way ANOVA with Bonferroni post hoc test, t(20) = −3.61, *p* = 0.011; Tau‐Ser396/404: 65.09 ± 7.09 vs 98.75 ± 7.64, One‐way ANOVA with Bonferroni post hoc test, t(20) = −3.70, *p* = 0.009), whereas PHTPP had no effect on estrogen's protective action (Tau‐Ser202/Thr205: 61.22 ± 7.02 vs 52.90 ± 7.61, One‐way ANOVA with Bonferroni post hoc test, t(20) = 0.74, *p* = 1; Tau‐Ser396/404: 65.09 ± 7.09 vs 65.65 ± 5.09, One‐way ANOVA with Bonferroni post hoc test, t(20) = −0.06, *p* = 1). We performed immunofluorescence staining to assess Tau‐Ser202/Thr205 expression in the dCA1 region. Compared to the sevoflurane group, estrogen significantly reduced Tau‐Ser202/Thr205 expression (Figure 4D,F, 59.00 ± 4.12 vs 28.83 ± 3.56, One‐way ANOVA with Bonferroni post hoc test, t(20) = 5.42, *p* < 0.001). This reduction was blocked by MPP (28.83 ± 3.56 vs 57.17 ± 4.58, One‐way ANOVA with Bonferroni post hoc test, t(20) = −5.09, *p* < 0.001), while PHTPP had no effect on the estrogen‐induced improvement (28.83 ± 3.56 vs 29.83 ± 3.36, One‐way ANOVA with Bonferroni post hoc test, t(20) = −0.18, *p* = 1).

To assess the effects of estrogen receptor antagonists on cognitive function, we conducted behavioral tests in mice. In the Morris water maze, estrogen significantly reduced the latency to find the platform compared to the sevoflurane group (Figure 4G,H; Figure S5A, 55.76 ± 2.03 vs 48.15 ± 2.03, Repeated measures ANOVA, F(1,18) = 9.03, *p =* 0.008, Supporting Information) and increased the number of platform crossings (Figure 4H, Mdn = 2.00, IQR = [1.00–2.25] vs Mdn = 5.00, IQR = [5.00–6.00], Kruskal‐Wallis test with Dunn's post hoc, Z = 3.81, *p* < 0.001). These improvements were blocked by MPP (Latency: 48.15 ± 2.12 vs 56.77 ± 2.12, Repeated measures ANOVA, F(1,18) = 8.30, *p =* 0.01; Crossings: Mdn = 5.00, IQR = [5.00–6.00] vs Mdn = 2.00, IQR = [1.00–2.00], Kruskal‐Wallis test with Dunn's post hoc, Z = 3.95, *p* < 0.001), whereas PHTPP had no effect (Latency: 48.15 ± 2.11 vs 48.87 ± 2.11, Repeated measures ANOVA, F(1,18) = 0.05, *p =* 0.812; Crossings: Mdn = 5.00, IQR = [5.00–6.00] vs Mdn = 5.00, IQR = [5.00–6.00], Kruskal‐Wallis test with Dunn's post hoc, Z = 0.14, *p* = 1). Similarly, in the Y‐maze test, estrogen increased the exploration time in the novel arm (Figure 4I; Figure S5B, 37.75 ± 3.42 vs 52.91 ± 3.61, One‐way ANOVA with Bonferroni post hoc test, t(20) = −3.32, *p* = 0.012, Supporting Information) and the number of entries (Figure 4J, 3.70 ± 0.30 vs 5.30 ± 0.42, One‐way ANOVA with Bonferroni post hoc test, t(20) = −3.16, *p* = 0.019) compared to the sevoflurane group. MPP reversed these effects (Exploration time: 52.91 ± 3.61 vs 36.08 ± 2.71, One‐way ANOVA with Bonferroni post hoc test, t(20) = 3.69, *p* = 0.004; Number of entries: 5.30 ± 0.42 vs 3.60 ± 0.31, One‐way ANOVA with Bonferroni post hoc test, t(20) = 3.36, *p* = 0.011), while PHTPP did not alter estrogen's beneficial impact (Exploration time: 52.91 ± 3.61 vs 52.47 ± 3.08, One‐way ANOVA with Bonferroni post hoc test, t(20) = 0.10, *p* = 1; Number of entries: 5.30 ± 0.42 vs 5.20 ± 0.39, One‐way ANOVA with Bonferroni post hoc test, t(20) = 0.20, *p* = 1).

We further investigated the effects of estrogen receptor antagonists on the synaptic marker PSD95. Compared with the sevoflurane group, estrogen treatment increased PSD95 expression (Figure S5C,D, 100.00 ± 5.53 vs 142.92 ± 6.20, One‐way ANOVA with Bonferroni post hoc test, t(20) = −4.76, *p* = 0.01, Supporting Information). This effect was blocked by MPP (142.92 ± 6.20 vs 102.40 ± 7.21, One‐way ANOVA with Bonferroni post hoc test, t(20) = 4.49, *p* = 0.01) but was not affected by PHTPP (142.92 ± 6.20 vs 139.07 ± 6.47, One‐way ANOVA with Bonferroni post hoc test, t(20) = 0.43, *p* = 1).

We also performed Golgi staining to evaluate dendritic morphology. Compared to the sevoflurane group, estrogen significantly increased dendritic spine density in the dCA1 region (Figure 4K,L, 1.82 ± 0.14 vs 2.46 ± 0.15, One‐way ANOVA with Bonferroni post hoc test, t(44) = −3.49, *p* = 0.007) and the number of dendritic branches (11.09 ± 0.37 vs 12.88 ± 0.37, Repeated measures ANOVA, F(1,10) = 11.92, *p =* 0.006). These enhancements were attenuated by MPP (Spine density: 2.46 ± 0.15 vs 1.82 ± 0.11, One‐way ANOVA with Bonferroni post hoc test, t(44) = 3.48, *p* = 0.007; Branching: 12.88 ± 0.33 vs 11.24 ± 0.33, Repeated measures ANOVA, F(1,10) = 12.42, *p =* 0.006), whereas PHTPP had no effect on the estrogen‐induced improvements (Spine density: 2.46 ± 0.15 vs 2.52 ± 0.11, One‐way ANOVA with Bonferroni post hoc test, t(44) = −0.31, *p* = 1; Branching: 12.88 ± 0.45 vs 12.77 ± 0.45, Repeated measures ANOVA, F(1,10) = 0.03, *p =* 0.871). We further found that, compared with the sevoflurane group, estrogen significantly increased the number of mushroom‐shaped dendritic spines in the dCA1 region (Figure S5E, 0.27 ± 0.02 vs 0.39 ± 0.03, one‐way ANOVA with Bonferroni post hoc test, t(44) = −3.11, *p* = 0.02, Supporting Information). This enhancement was attenuated by MPP (0.39 ± 0.03 vs 0.27 ± 0.03, One‐way ANOVA with Bonferroni post hoc test, t(44) = 3.29, *p* = 0.012), whereas PHTPP had no effect on estrogen‐induced improvement (0.39 ± 0.03 vs 0.39 ± 0.03, One‐way ANOVA with Bonferroni post hoc test, t(44) = −0.13, *p* = 1).

### 2.5 Estrogen Combined with AAV‐ERα‐O Ameliorated Tau Hyperphosphorylation and Cognitive Deficits in Aged Mice Exposed to Sevoflurane

**Figure 5:**
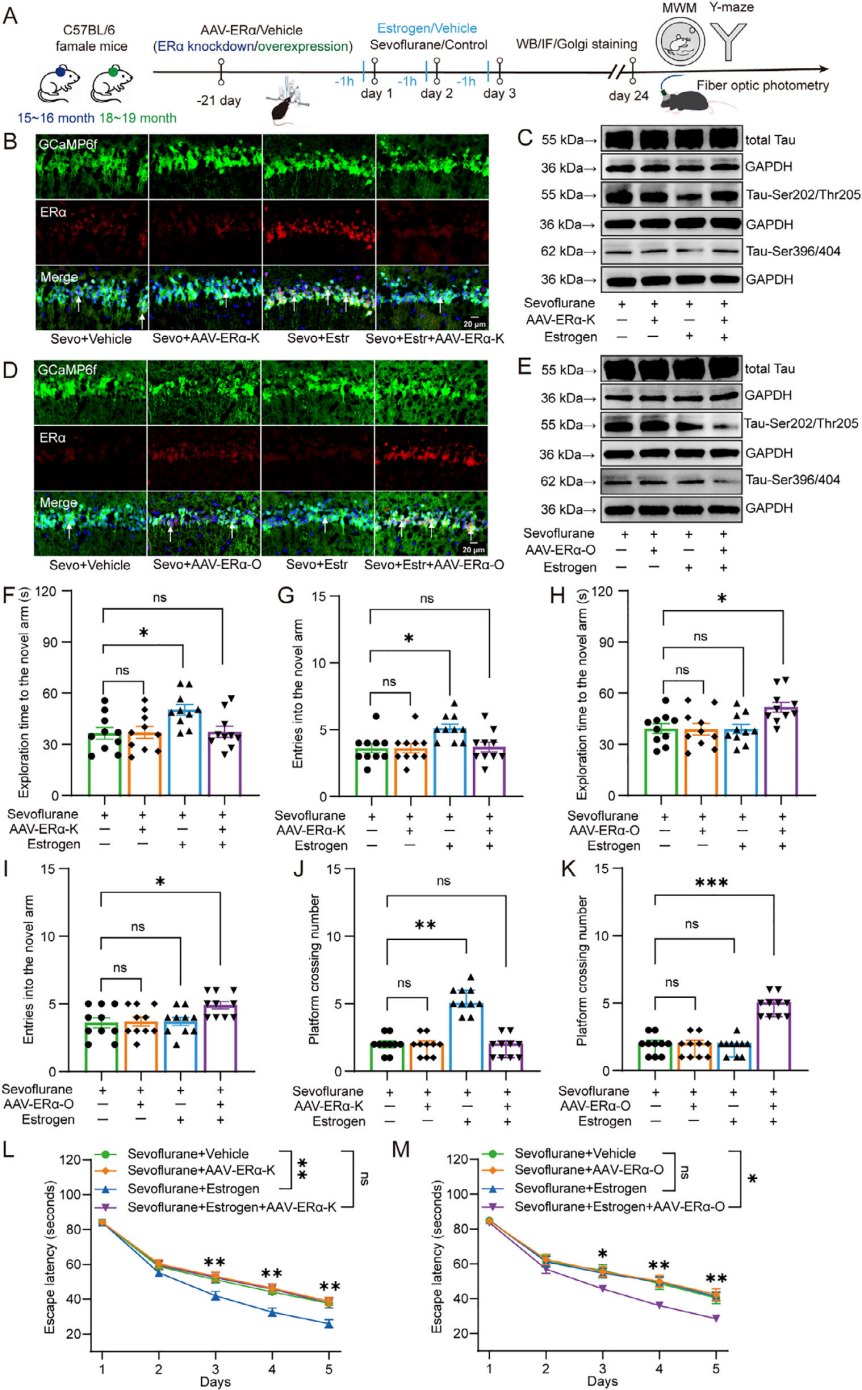
Effects of estrogen combined with viral injection and sevoflurane on Tau phosphorylation and cognitive function in the hippocampus of middle‐aged and aged female mice. A) Timeline of the experimental design for this part of the study. B,D) Representative immunofluorescence images showing co‐expression of ERα and pyramidal neurons in middle‐aged (B) and aged mice (D), respectively. C,E) Representative Western blot images showing Tau‐Ser202/Thr205 and Tau‐Ser396/404 levels in the hippocampus of middle‐aged (C) and aged mice (E), respectively. F,G) Time spent exploring the novel arm and the number of entries into the novel arm in the Y‐maze task for middle‐aged female mice (*n* = 10 per group). H,I) Time spent exploring the novel arm and the number of entries into the novel arm in the Y‐maze task for aged female mice (*n* = 10 per group). J,K) Latency to locate the hidden platform and number of platform crossings in the Morris water maze task for middle‐aged female mice (*n* = 10 per group). L,M) Latency to locate the hidden platform and number of platform crossings in the Morris water maze task for middle‐aged female mice (*n* = 10 per group). Data in (F), (G), (H), (I), (L), and (M) are presented as mean ± SEM; data in (J) and (K) are presented as median and IQR. Statistical analysis: One‐way ANOVA with Bonferroni post hoc test was used for (F), (G), (H), and (I); Kruskal‐Wallis test with Dunn's post hoc was used for (J) and (K); Repeated measures ANOVA was applied to (L) and (M). Abbreviations: Estrogen (Estr); Sevoflurane (Sevo). ns, no significance; *p < 0.05; **p < 0.01; ***p < 0.001.

To evaluate the impact of ERα protein expression on the efficacy of estrogen treatment, we first examined ERα expression levels across different age groups. The detailed experimental workflow is illustrated in **Figure** **5**A. We found that ERα protein levels significantly declined with increasing age (Figure S6A,B, 10.00 ± 4.46 vs 69.98 ± 4.55 vs 30.34 ± 3.98; One‐way ANOVA, F(2,15) = 64.48, *p* < 0.001, Supporting Information). We further validated the efficiency of viral modulation. Compared with the control vector, AAV‐ERα‐K significantly reduced ERα protein expression (Figure S6C,D, 100.00 ± 12.04 vs 72.45 ± 11.03, Unpaired *t*‐test, t(10) = 4.13, *p =* 0.002, Supporting Information), while AAV‐ERα‐O markedly increased ERα protein levels (Figure S6E,F, 100.00 ± 11.19 vs 137.77 ± 14.86, Unpaired *t*‐test, t(10) = −4.97, *p =* 0.001, Supporting Information).

To further evaluate the effect of ERα expression on estrogen responsiveness, we performed Western blot analysis. The results showed that in middle‐aged mice, AAV‐ERα‐K significantly reduced ERα expression compared to the sevoflurane group (Figure S6G,H, 100.00 ± 4.17 vs 75.44 ± 4.83, One‐way ANOVA with Bonferroni post hoc test, t(20) = −3.77, *p* = 0.007, Supporting Information), whereas estrogen treatment significantly increased ERα levels (100.00 ± 4.17 vs 120.57 ± 3.09, One‐way ANOVA with Bonferroni post hoc test, t(20) = −3.15, *p* = 0.03). In aged mice, AAV‐ERα‐O significantly elevated ERα protein levels (Figure S6I,J, 100.00 ± 7.83 vs 132.92 ± 6.24, One‐way ANOVA with Bonferroni post hoc test, t(20) = −3.78, *p* = 0.007, Supporting Information), while estrogen alone had no effect (100.00 ± 7.83 vs 105.80 ± 5.36, One‐way ANOVA with Bonferroni post hoc test, t(20) = −0.67, *p* = 1). Notably, combined treatment with estrogen and the AAV‐ERα‐O further augmented ERα expression relative to overexpression alone (132.92 ± 6.24 vs 158.84 ± 4.74, One‐way ANOVA with Bonferroni post hoc test, t(20) = −2.98, *p* = 0.045). To assess ERα expression specifically in pyramidal neurons, we labeled these cells with GCaMP6f and performed co‐immunostaining for ERα. In middle‐aged mice, AAV‐ERα‐K reduced the number of ERα‐positive pyramidal neurons (Figure 5B; Figure S6K, 40.00 ± 3.82 vs 26.50 ± 1.89, One‐way ANOVA with Bonferroni post hoc test, t(20) = 3.15, *p* = 0.03, Supporting Information), whereas estrogen treatment significantly increased their number (40.00 ± 3.82 vs 55.33 ± 3.41, One‐way ANOVA with Bonferroni post hoc test, t(20) = −3.57, *p* = 0.011). In aged mice, AAV‐ERα‐O enhanced ERα co‐localization with pyramidal neurons (Figure 5D; Figure S6L, 26.67 ± 2.40 vs 40.83 ± 3.67, One‐way ANOVA with Bonferroni post hoc test, t(20) = −3.38, *p* = 0.018, Supporting Information), while estrogen alone had no significant effect (26.67 ± 2.40 vs 27.50 ± 2.49, One‐way ANOVA with Bonferroni post hoc test, t(20) = −0.20, *p* = 1). Importantly, co‐treatment with estrogen and the AAV‐ERα‐O further increased the number of ERα‐positive pyramidal neurons compared to overexpression alone (40.83 ± 3.67 vs 54.17 ± 3.11, One‐way ANOVA with Bonferroni post hoc test, t(20) = −3.18, *p* = 0.028), suggesting that ERα overexpression reestablishes neuronal sensitivity to estrogen.

To investigate the role of ERα expression in estrogen‐mediated neuroprotection, we assessed hippocampal Tau phosphorylation via Western blot. In middle‐aged mice, estrogen significantly reduced hippocampal Tau‐Ser202/Thr205 (Figure 5C; Figure S6M, 100.00 ± 4.49 vs 60.87 ± 5.66, One‐way ANOVA with Bonferroni post hoc test, t(20) = 4.96, *p* < 0.001, Supporting Information) and Tau‐Ser396/404 (Figure S6N, 100.00 ± 6.07 vs 56.37 ± 5.17, One‐way ANOVA with Bonferroni post hoc test, t(20) = 5.89, *p* < 0.001, Supporting Information) expression compared to the sevoflurane group. However, AAV‐ERα‐K abolished this effect (Tau‐Ser202/Thr205: 100.00 ± 4.49 vs 101.66 ± 7.13, One‐way ANOVA with Bonferroni post hoc test, t(20) = −0.21, *p* = 1; Tau‐Ser396/404: 100.00 ± 6.07 vs 94.61 ± 5.38, One‐way ANOVA with Bonferroni post hoc test, t(20) = 0.73, *p* = 1). In aged mice, estrogen alone did not affect Tau‐Ser202/Thr205 (Figure 5E; Figure S6O, 100.00 ± 4.40 vs 96.89 ± 8.89, One‐way ANOVA with Bonferroni post hoc test, t(20) = 0.39, *p* = 1, Supporting Information) or Tau‐Ser396/404 (Figure S6P, 100.00 ± 7.35 vs 94.63 ± 7.10, One‐way ANOVA with Bonferroni post hoc test, t(20) = 0.57, *p* = 1, Supporting Information) expression. Notably, estrogen combined with AAV‐ERα‐O significantly reduced both Tau‐Ser202/Thr205 (100.00 ± 4.40 vs 47.28 ± 3.92, One‐way ANOVA with Bonferroni post hoc test, t(20) = 6.57, *p* < 0.001) and Tau‐Ser396/404 (100.00 ± 7.35 vs 53.87 ± 6.01, One‐way ANOVA with Bonferroni post hoc test, t(20) = 4.86, *p* = 0.001) levels. Immunofluorescence staining yielded consistent results. In middle‐aged mice, estrogen markedly reduced Tau‐Ser202/Thr205 expression in the dCA1 region (Figure S7A,B, 54.17 ± 3.40 vs 31.50 ± 2.93, One‐way ANOVA with Bonferroni post hoc test, t(20) = 4.42, *p* = 0.002, Supporting Information), an effect blocked by AAV‐ERα‐K (54.17 ± 3.40 vs 54.67 ± 3.05, One‐way ANOVA with Bonferroni post hoc test, t(20) = −0.10, *p* = 1). In aged mice, estrogen alone had no effect on dCA1 Tau‐Ser202/Thr205 levels (Figure S7C,D, 55.00 ± 3.99 vs 57.83 ± 3.77, One‐way ANOVA with Bonferroni post hoc test, t(20) = −0.53, *p* = 1, Supporting Information), while co‐administration with AAV‐ERα‐O significantly reduced Tau‐Ser202/Thr205 expression (55.00 ± 3.99 vs 30.33 ± 2.93, One‐way ANOVA with Bonferroni post hoc test, t(20) = 4.64, *p* = 0.001).

We evaluated the impact of ERα expression on cognitive function using the Morris water maze and Y‐maze tests. In the Morris water maze, estrogen significantly reduced the latency to locate the hidden platform (Figure 5L; Figure S7E, 55.18 ± 1.75 vs 48.00 ± 1.75, Repeated measures ANOVA, F(1,18) = 8.44, *p =* 0.009, Supporting Information) and increased the number of platform crossings (Figure 5J, Mdn = 2.00, IQR = [1.75–2.25] vs Mdn = 5.00, IQR = [4.75–6.00], Kruskal‐Wallis test with Dunn's post hoc, Z = 3.73, *p* = 0.001) in middle‐aged mice compared to the sevoflurane group. However, these effects were abolished by AAV‐ERα‐K (Latency: 55.18 ± 2.08 vs 55.88 ± 2.08, Repeated measures ANOVA, F(1,18) = 0.06, *p =* 0.815; Crossings: Mdn = 2.00, IQR = [1.75–2.25] vs Mdn = 2.00, IQR = [1.00–2.25], Kruskal‐Wallis test with Dunn's post hoc, Z = 0.48, *p* = 1). In aged mice, estrogen alone did not significantly alter latency (Figure 5M; Figure S7F, 58.29 ± 2.56 vs 55.37 ± 2.56, Repeated measures ANOVA, F(1,18) < 0.001, *p =* 0.983, Supporting Information) or platform crossings (Figure 5K, Mdn = 2.00, IQR = [1.00–2.25] vs Mdn = 2.00, IQR = [1.00–2.00], Kruskal‐Wallis test with Dunn's post hoc, Z = 0.20, *p* = 1) relative to the sevoflurane group. Notably, co‐administration of estrogen and the AAV‐ERα‐O markedly improved performance, as evidenced by reduced latency (58.29 ± 2.16 vs 50.17 ± 2.16, Repeated measures ANOVA, F(1,18) = 7.06, *p =* 0.016) and increased platform crossings (Mdn = 2.00, IQR = [1.00–2.25] vs Mdn = 5.00, IQR = [4.00–5.25], Kruskal‐Wallis test with Dunn's post hoc, Z = 3.82, *p* < 0.001). In the Y‐maze test, estrogen significantly increased both the time spent in the novel arm (Figure 5F; Figure S7G, 36.44 ± 3.43 vs 50.21 ± 3.56, One‐way ANOVA with Bonferroni post hoc test, t(36) = −2.90, *p* = 0.037, Supporting Information) and the number of novel arm entries (Figure 5G, 3.60 ± 0.34 vs 5.10 ± 0.31, One‐way ANOVA with Bonferroni post hoc test, t(36) = −3.11, *p* = 0.022) in middle‐aged mice compared to the sevoflurane group, whereas AAV‐ERα‐K abolished these effects (Exploration time: 36.44 ± 3.43 vs 37.34 ± 3.28, One‐way ANOVA with Bonferroni post hoc test, t(36) = −0.19, *p* = 1; Entries: 3.60 ± 0.34 vs 3.70 ± 0.37, One‐way ANOVA with Bonferroni post hoc test, t(36) = −0.21, *p* = 1). In aged mice, estrogen alone had no significant effect on novel arm exploration time (Figure 5H; Figure S7H, 39.22 ± 2.87 vs 38.93 ± 2.77, One‐way ANOVA with Bonferroni post hoc test, t(36) = 0.07, *p* = 1, Supporting Information) or the number of novel arm entries (Figure 5I, 3.60 ± 0.37 vs 3.70 ± 0.30, One‐way ANOVA with Bonferroni post hoc test, t(36) = −0.22, *p* = 1). However, combined treatment with estrogen and AAV‐ERα‐O significantly enhanced both exploration time (39.22 ± 2.87 vs 51.73 ± 2.97, One‐way ANOVA with Bonferroni post hoc test, t(36) = −2.93, *p* = 0.035) and number of entries into the novel arm (3.60 ± 0.37 vs 4.90 ± 0.28, One‐way ANOVA with Bonferroni post hoc test, t(36) = −2.85, *p* = 0.043).

We further examined the effect of ERα expression on PSD95 levels. In middle‐aged mice, estrogen treatment significantly increased hippocampal PSD95 expression compared with the sevoflurane group (Figure S7I,J, 100.00 ± 11.51 vs 144.21 ± 8.51, One‐way ANOVA with Bonferroni post hoc test, t(20) = −3.42, *p* = 0.016, Supporting Information); however, this effect was abolished by AAV‐ERα‐K (100.00 ± 11.51 vs 102.79 ± 7.48, One‐way ANOVA with Bonferroni post hoc test, t(20) = −0.22, *p* = 1). In aged mice, estrogen alone did not significantly alter PSD95 expression compared with the sevoflurane group (Figure S7K,L, 100.00 ± 8.34 vs 98.63 ± 8.20, One‐way ANOVA with Bonferroni post hoc test, t(20) = 0.12, *p* = 1, Supporting Information). Notably, only the combined treatment of estrogen and AAV‐ERα‐O significantly upregulated PSD95 levels (100.00 ± 8.34 vs 135.70 ± 8.11, One‐way ANOVA with Bonferroni post hoc test, t(20) = −3.21, *p* = 0.027).

These findings suggest that ERα expression is required for the beneficial cognitive effects of estrogen in sevoflurane‐exposed mice, and that restoring ERα expression can rescue the responsiveness to estrogen, particularly in aged animals.

### 2.6 Estrogen Combined with AAV‐ERα‐O Enhanced Neuronal Ca^2^⁺ Signaling Fluctuations and Firing Activity, and Improved Dendritic Morphology in the dCA1 Region of Aged Mice

**Figure 6:**
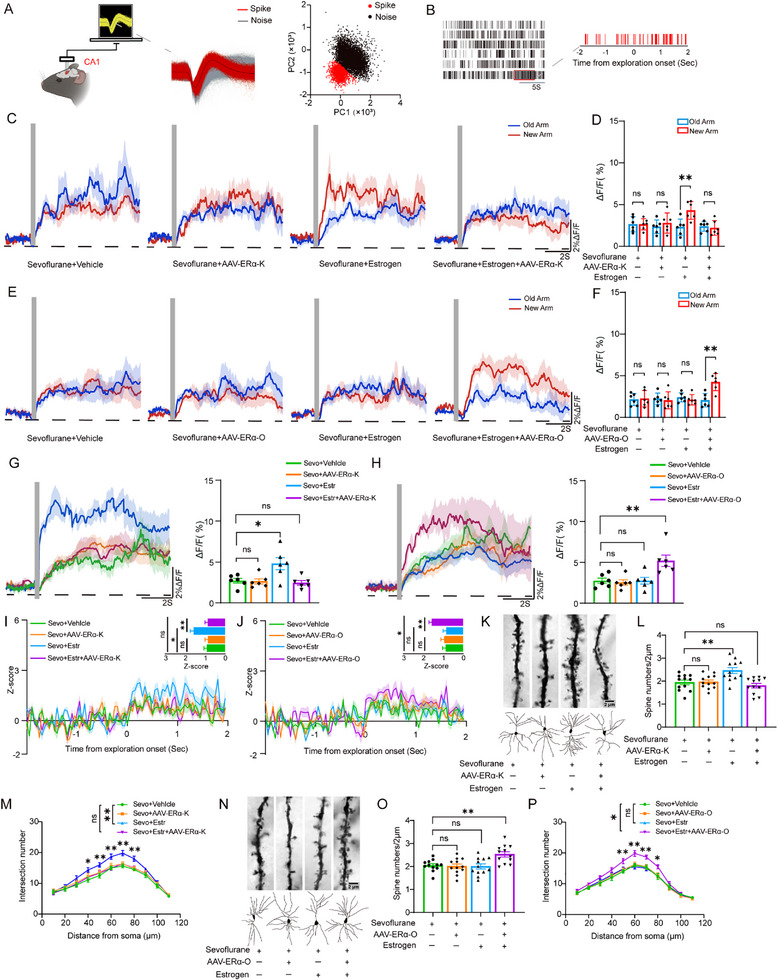
Effects of estrogen combined with viral injection and sevoflurane on spontaneous Ca^2^⁺ activity, neuronal firing, and dendritic spine morphology in dCA1 neurons of middle‐aged and aged female mice. A,B) Schematic of the process for in vivo electrophysiological recordings of clustered cells and the extraction of behavior‐associated firing data. C,D) Representative traces and quantification of spontaneous Ca^2^⁺ signal fluctuations in middle‐aged female mice during exploration of the novel versus familiar arms in the Y‐maze (*n* = 6 per group). E,F) Representative traces and quantification of spontaneous Ca^2^⁺ signal fluctuations in aged female mice during exploration of the novel versus familiar arms in the Y‐maze (*n* = 6 per group). G,H) Transient Ca^2^⁺ signal fluctuations upon entry into the novel arm in middle‐aged (G) and aged (H) female mice (left: representative trace; right: quantification; *n* = 6 per group). I) Visualization and quantification of neuronal firing frequency in middle‐aged female mice during exploration of a novel object (14 cells from *n* = 6 mice per group). J) Visualization and quantification of neuronal firing frequency in aged female mice during exploration of a novel object (14 cells from *n* = 6 mice per group). K) Representative images of dendritic spines (top) and dendritic shafts (bottom) in dCA1 neurons of middle‐aged female mice. L,M) Quantification of dendritic spine density (L) and dendritic shaft number (M) in dCA1 neurons of middle‐aged female mice (*n* = 6 per group). N) Representative images of dendritic spines (top) and dendritic shafts (bottom) in dCA1 neurons of aged female mice. O,P) Quantification of dendritic spine density (O) and dendritic shaft number (P) in dCA1 neurons of aged female mice (*n* = 6 per group). Data in (D) and (F) are presented as mean ± SD; data in (G), (H), (I), (J), (L), (M), (O), and (P) are presented as mean ± SEM. Statistical analysis: Unpaired *t*‐test was used for (D) and (F); One‐way ANOVA with Bonferroni post hoc test was applied to (G), (H), (I), (J), (L), and (O); Repeated measures ANOVA was applied to (M) and (P). ns, no significance; *p < 0.05; **p < 0.01.

To further investigate the effect of ERα expression on hippocampal neuronal activity, we employed fiber photometry to monitor neuronal Ca^2^⁺ dynamics in mice. The results showed that sevoflurane eliminated the difference in Ca^2^⁺ signal amplitude between exploration of the novel and familiar arms in both middle‐aged (Figure 6C,D, 2.64 ± 0.90 vs 2.63 ± 0.67, Unpaired *t*‐test, t(10) = 0.02, *p =* 0.983) and aged mice (Figure 6E,F, 2.14 ± 0.72 vs 2.25 ± 0.99, Unpaired *t*‐test, t(10) = 0.21, *p =* 0.838). Estrogen restored the fluctuation in Ca^2^⁺ signal amplitude during exploration of the novel vs familiar arms in middle‐aged mice exposed to sevoflurane (2.29 ± 0.97 vs 4.29 ± 1.08, Unpaired *t*‐test, t(10) = −3.38, *p =* 0.007), and also increased the amplitude of transient Ca^2^⁺ signals upon entry into the novel arm (Figure 6G, 2.70 ± 0.29 vs 4.78 ± 0.74, One‐way ANOVA with Bonferroni post hoc test, t(20) = −3.27, *p* = 0.023). However, AAV‐ERα‐K abolished both effects (Exploration: 2.38 ± 0.58 vs 2.19 ± 0.89, Unpaired *t*‐test, t(10) = 0.44, *p =* 0.672; Novel arm entry: 2.70 ± 0.29 vs 2.44 ± 0.28, One‐way ANOVA with Bonferroni post hoc test, t(20) = 0.41, *p* = 1). In aged mice, estrogen alone did not affect the fluctuation of Ca^2^⁺ signal amplitude between the novel and familiar arms (2.40 ± 0.55 vs 2.11 ± 0.63, Unpaired *t*‐test, t(10) = 0.84, *p =* 0.422), nor the amplitude of transient Ca^2^⁺ signals upon novel arm entry (Figure 6H, 2.74 ± 0.32 vs 2.75 ± 0.41, One‐way ANOVA with Bonferroni post hoc test, t(20) = −0.01, *p* = 1). However, combined treatment with estrogen and AAV‐ERα‐O restored the fluctuation in Ca^2^⁺ signal amplitude during exploration (2.06 ± 0.81 vs 4.23 ± 1.03, Unpaired *t*‐test, t(10) = −4.08, *p =* 0.002) and significantly increased the transient Ca^2^⁺ signal amplitude upon novel arm entry (2.74 ± 0.32 vs 5.19 ± 0.70, One‐way ANOVA with Bonferroni post hoc test, t(20) = −3.74, *p* = 0.008) in sevoflurane‐exposed aged mice.

We also conducted in vivo electrophysiological recordings, following the procedures illustrated in **Figure** **6**A,B. In middle‐aged mice, compared with the sevoflurane group, estrogen significantly enhanced the firing rate of hippocampal dCA1 neurons during novel object exploration (time 0 indicates the onset of exploratory behavior; Figure 6I; Figure S8A, 0.91 ± 0.16 vs 1.57 ± 0.18, One‐way ANOVA with Bonferroni post hoc test, t(52) = −2.85, *p* = 0.038, Supporting Information). However, this estrogen‐induced enhancement was abolished by AAV‐ERα‐K (0.91 ± 0.16 vs 0.85 ± 0.15, One‐way ANOVA with Bonferroni post hoc test, t(52) = 0.23, *p* = 1). In aged mice, compared with the sevoflurane group, estrogen alone did not alter the neuronal firing rate in the dCA1 region during novel object exploration (Figure 6J; Figure S8B, 0.87 ± 0.16 vs 0.85 ± 0.16, One‐way ANOVA with Bonferroni post hoc test, t(52) = 0.13, *p* = 1, Supporting Information). In contrast, estrogen combined with AAV‐ERα‐O significantly increased the neuronal firing rate in the dCA1 region (0.87 ± 0.16 vs 1.57 ± 0.14, One‐way ANOVA with Bonferroni post hoc test, t(52) = −3.14, *p* = 0.017).

Golgi staining revealed that, in middle‐aged mice, estrogen significantly increased dendritic spine density (Figure 6K–M, 1.95 ± 0.10 vs 2.47 ± 0.13, One‐way ANOVA with Bonferroni post hoc test, t(44) = −3.52, *p* = 0.006) and the number of dendritic branches (11.03 ± 0.40 vs 13.26 ± 0.40, Repeated measures ANOVA, F(1,10) = 15.91, *p =* 0.003) in the dCA1 region compared with the sevoflurane group. However, these enhancements were abolished by AAV‐ERα‐K (Spine density: 1.95 ± 0.10 vs 1.80 ± 0.10, One‐way ANOVA with Bonferroni post hoc test, t(44) = 0.95, *p* = 1; Branching: 11.03 ± 0.40 vs 11.44 ± 0.40, Repeated measures ANOVA, F(1,10) = 0.53, *p =* 0.484). In aged mice, estrogen alone had no significant effect on dendritic spine density (Figure 6N–P, 2.05 ± 0.07 vs 2.00 ± 0.11, One‐way ANOVA with Bonferroni post hoc test, t(44) = 0.30, *p* = 1) or branching (10.59 ± 0.49 vs 10.73 ± 0.49, Repeated measures ANOVA, F(1,10) = 0.04, *p =* 0.849) compared with the sevoflurane group. Notably, co‐administration of estrogen with AAV‐ERα‐O significantly enhanced both spine density (2.05 ± 0.07 vs 2.53 ± 0.11, One‐way ANOVA with Bonferroni post hoc test, t(44) = −3.42, *p* = 0.008) and dendritic branching (10.59 ± 0.57 vs 12.46 ± 0.57, Repeated measures ANOVA, F(1,10) = 5.38, *p =* 0.043). We further found that, compared to the sevoflurane group, estrogen significantly increased the number of mushroom‐type dendritic spines in the dCA1 region of middle‐aged mice (Figure S8C, 0.29 ± 0.02 vs 0.38 ± 0.03, one‐way ANOVA with Bonferroni post hoc test, t(44) =  −3.02, *p* = 0.025, Supporting Information). However, this enhancement was abolished by AAV‐ERα‐K (0.29 ± 0.02 vs 0.28 ± 0.02, t(44)  =  0.22, *p*  =  1). In aged mice, estrogen alone did not significantly affect mushroom spine density compared to the sevoflurane group Figure S8D, 0.27 ± 0.02 vs 0.28 ± 0.02, one‐way ANOVA with Bonferroni post hoc test, t(44)  =  −0.43, *p*  =  1, Supporting Information). Notably, co‐administration of estrogen with AAV‐ERα‐O significantly increased the number of mushroom‐type spines (0.27 ± 0.02 vs 0.37 ± 0.02, one‐way ANOVA with Bonferroni post hoc test, t(44)  =  −3.48, *p*  =  0.007), suggesting that enhanced ERα availability is critical for the restoration of estrogen‐mediated synaptic plasticity in aged mice.

### 2.7 Estrogen Competitively Inhibits the Interaction Between Tau Protein and ERα

**Figure 7:**
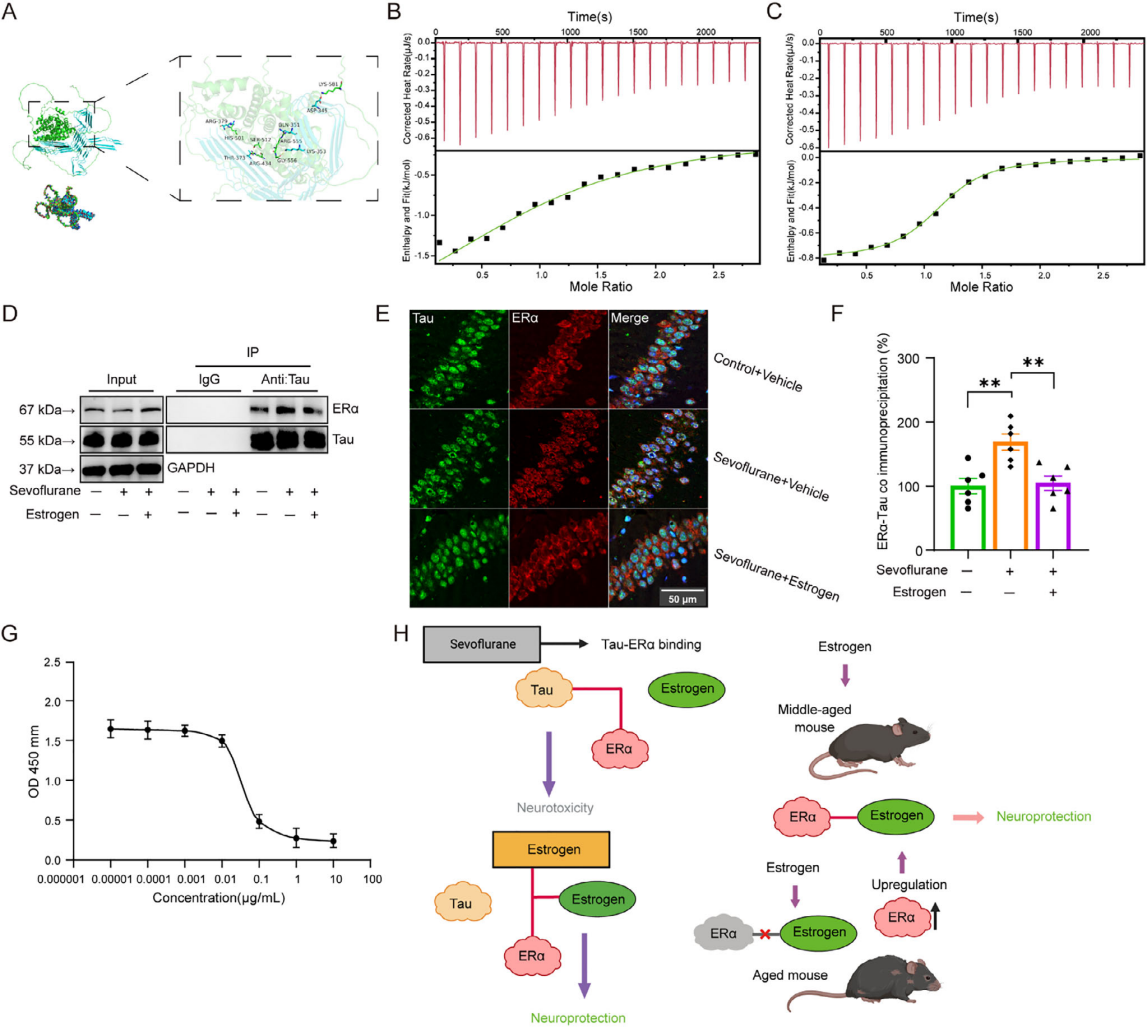
The predicted and experimentally validated interaction between Tau and ERα proteins, as well as the competitive inhibition of this interaction by estrogen. A) On the left, a schematic diagram of molecular docking; on the right, hydrogen bond docking; and below, a cartoon illustration of the docking process. B) and C) ITC results of Tau and Tau following sevoflurane treatment titrated with ERα receptor. Upper panels show raw heat changes recorded during the ITC titration process, while lower panels depict the corresponding nonlinear curve fitting of the binding isotherms. D) Representative co‐immunoprecipitation showing the effects of sevoflurane and estrogen on the interaction between Tau and ERα. E,F) Representative immunofluorescence images (E) and corresponding quantification (F) of Tau and ERα colocalization in hippocampal neurons. G) Competitive inhibition of estrogen on Tau binding to ERα. H) Schematic illustration of proposed mechanisms. This schematic illustrates sevoflurane‐induced neurotoxicity and estrogen‐mediated neuroprotection, along with the age‐dependent effects of estrogen in middle‐aged and aged mice. Data in (F) is presented as mean ± SEM. Statistical analysis: One‐way ANOVA with Bonferroni post hoc test was applied to (F). **p < 0.01.

To investigate whether Tau affects ERα responsiveness to estrogen, we first performed a protein–protein interaction prediction analysis. As shown in **Figure** **7**A, Tau was predicted to bind ERα with a binding energy of −19.7 kcal mol^−1^, indicating a strong binding potential. Several hydrogen bonds were identified at key residues, including LYS‐581 and ASP‐345.

To experimentally validate this predicted interaction, we conducted ITC. As illustrated in Figure 7B, titration of Tau protein into ERα solution resulted in measurable heat release (exothermic reaction), confirming a direct physical interaction. The upper panel shows the raw thermogram (heat flow over time), while the lower panel presents the nonlinear fitting curve based on an independent binding model. The calculated parameters were as follows: dissociation constant (Kd) = 3.28 × 10^−5^ m, enthalpy change (ΔH) = −2.62 kJ mol^−1^, entropy change (ΔS) = 77.07 J mol^−1^·K^−1^, with a binding stoichiometry (n) close to 1. The negative ΔH and positive ΔS suggest that the interaction is enthalpy‐driven, likely involving hydrogen bonding, consistent with our molecular docking prediction.

Following repeated sevoflurane anesthesia, we re‐evaluated Tau–ERα binding using Tau protein extracted from treated mice. As shown in Figure 7C, the binding affinity was markedly enhanced, with a lower Kd of 2.20 × 10^−6^ m, ΔH = −0.81 kJ mol^−1^, and ΔS = 105.6 J mol^−1^ K^−1^. The increase in ΔS suggests a more disordered interaction post‐sevoflurane exposure.

In addition, co‐immunoprecipitation assays revealed that sevoflurane markedly enhanced the interaction between Tau and ERα, whereas estrogen significantly attenuated this interaction (Figure 7D). Consistently, immunofluorescence analysis showed that sevoflurane markedly increased the number of cells exhibiting Tau–ERα colocalization (Figure 7E,F, 100.00 ± 11.89 vs 168.57 ± 12.56; one‐way ANOVA with Bonferroni post hoc test, t(15) = −4.08, *p* = 0.003), while estrogen significantly reduced the number of colocalized cells (168.57 ± 12.56 vs 104.57 ± 11.15; one‐way ANOVA with Bonferroni post hoc test, t(15) = −3.81, *p* = 0.005).

Finally, to assess the effect of estrogen on the Tau–ERα interaction, we performed a competitive ELISA. The results showed that estrogen competes with Tau for ERα binding and effectively displaces Tau from the complex in a concentration‐dependent manner, with an IC50 of 376.2 ng mL^−1^ (Figure 7B). These findings indicate that estrogen competitively inhibits the binding of Tau to ERα.

## 3 Discussion

To investigate whether the neurotoxic effects of sevoflurane exhibit sex differences and whether estrogen exerts therapeutic effects, we conducted comparative experiments using female and male mice across different age groups. Our results demonstrated that the neurotoxic effects of sevoflurane are sex‐specific, as it induced cognitive dysfunction in middle‐aged female mice but not in male mice. Moreover, estrogen alleviated sevoflurane‐induced neurotoxicity in middle‐aged female mice via ERα, though this effect was abolished by ERα knockdown. In contrast, in aged female mice, estrogen alone did not confer cognitive benefits unless ERα was concurrently overexpressed, highlighting the critical role of ERα expression in mediating the neuroprotective effects of estrogen. Additionally, we observed a competitive interaction between estrogen and the Tau protein at the ERα site. This finding provides new insights into the relationship between estrogen signaling and Tau pathology in anesthetic‐induced cognitive dysfunction.

Consistent with previous studies, our results showed that sevoflurane anesthesia did not induce neurotoxicity in adult mice but led to increased hippocampal Tau phosphorylation and cognitive dysfunction in aged mice.^[^ ^20^ ^]^ Earlier studies have reported that after sevoflurane exposure, neonatal female mice tend to favor M2 polarization and exhibit greater resistance to sevoflurane‐induced neurotoxicity.^[^ ^6^ ^]^ Other research has shown that sevoflurane exposure can cause long‐term cognitive impairment in neonatal female mice during early adulthood, whereas male mice are unaffected.^[^ ^21^ ^]^ Although discrepancies among these results may be partly due to differences in anesthetic agents and dosages, they collectively underscore the presence of sex‐specific differences in anesthetic‐induced neurotoxicity during rapid brain development. Our study extends these findings to mature brains, demonstrating that repeated sevoflurane anesthesia in middle‐aged mice also exhibits sex‐specific effects: female mice show increased hippocampal Tau phosphorylation and cognitive dysfunction, while male mice remain unaffected. This difference may be attributed to the inherent differences between male and female sex hormones.

In addition to promoting Tau protein phosphorylation, sevoflurane also impairs hippocampal neuronal activity, which represents another key aspect of its neurotoxic effects. Studies have shown that following sevoflurane anesthesia, hippocampal neuronal activity is significantly suppressed, characterized by reduced neuronal firing rates and delayed synaptic transmission.^[^ ^22^, ^23^ ^]^ Fiber photometry is commonly used to assess neuronal activity,^[^ ^24^ ^]^ therefore, we employed fiber photometry combined with in vivo electrophysiology to monitor Ca^2^⁺ signal fluctuations and neuronal firing frequency in mice. Our results demonstrated that sevoflurane exposure led to decreased amplitude of transient Ca^2^⁺ signals and reduced firing frequency in hippocampal dCA1 neurons of middle‐aged and aged female mice, consistent with previous findings.^[^ ^25^, ^26^ ^]^ Golgi staining further revealed that sevoflurane caused a reduction in dendritic spine density and the number of primary dendrites in the dCA1 region of the hippocampus in both middle‐aged and aged female mice.^[^ ^27^, ^28^ ^]^ Given that estrogen can also be locally synthesized and exert functional effects within the hippocampus, we measured hippocampal estrogen levels.^[^ ^10^, ^11^ ^]^ Consistent with previous studies showing that estrogen enhances neuronal responsiveness and improves electrical activity, our results demonstrate that estrogen improves transient Ca^2^⁺ signal fluctuations and firing frequency in the dCA1 neurons of mice.^[^ ^29^, ^30^ ^]^ Interestingly, we also observed results similar to those from HRT studies: estrogen supplementation was able to alleviate sevoflurane‐induced neuronal damage in middle‐aged female mice but failed to do so in aged female mice. We also found that sevoflurane reduced PSD95 expression in the hippocampus of mice. Estrogen treatment increased PSD95 expression in the hippocampus of middle‐aged mice exposed to sevoflurane, but had no such effect in aged mice. These findings highlight a limitation of estrogen therapy in treating sevoflurane‐induced neurotoxicity.

The decline in estrogen therapy efficacy with advancing age or prolonged hormone deprivation suggests the existence of a critical window governed by molecular responsiveness rather than hormone availability alone.^[^ ^31^, ^32^ ^]^ In our study, estrogen failed to rescue sevoflurane‐induced neurotoxicity in aged female mice, indicating a loss of therapeutic responsiveness beyond the critical window.^[^ ^33^, ^34^ ^]^ Our results also confirmed that both hippocampal estrogen levels and ERα receptor expression significantly decline with age in female mice. Interestingly, upregulation of ERα expression in the hippocampus restored estrogen‐mediated neuroprotection, suggesting that the availability of ERα is a critical determinant for the success of the treatment. Evidence from ERβ knockout models suggests that ERβ deletion exacerbates cognitive deficits by impairing ERα‐driven transcription, thereby sensitizing the hippocampus to aging‐related stressors.^[^ ^35^, ^36^ ^]^ Studies using viral vector‐mediated expression of hippocampal ERα and ERβ have demonstrated that ERα is crucial for maintaining cognitive function in young animals.^[^ ^37^, ^38^ ^]^ Other studies have shown that ERα can activate PI3K and Akt, thereby inhibiting GSK3β activity and reducing Tau phosphorylation.^[^ ^39^, ^40^ ^]^ Furthermore, independent enhancement of ERα expression has been shown to restore cognitive function in young ERα KO mice as well as in middle‐aged and aged female rodents, highlighting the central role of ERα in memory maintenance.^[^ ^41^, ^42^, ^43^ ^]^ Consistent with these findings, we demonstrated that inhibition or knockdown of ERα abolished estrogen's neuroprotective effects against sevoflurane exposure. Interestingly, we found that estrogen could upregulate ERα expression in middle‐aged mice, but not in naturally aged mice unless combined with ERα overexpression, suggesting that age‐associated ERα degradation underlies the loss of estrogen responsiveness. This aligns with prior observations that ERαKO mice exhibit blunted responses to estrogen and that targeted restoration of ERα expression reinstates hormonal sensitivity.^[^ ^44^, ^45^ ^]^ Collectively, in summary, these results highlight that ERα degradation and the consequent decline in ERα responsiveness to estrogen are central mechanisms limiting the efficacy of delayed estrogen treatment.

Sevoflurane anesthesia induces excessive phosphorylation of Tau protein, whereas physiological aging elevates total Tau levels, suggesting that Tau abnormalities may contribute to adverse effects.^[^ ^46^, ^47^ ^]^ Considering that the therapeutic window of estrogen may be constrained by a decline in ERα responsiveness, we hypothesized that sevoflurane‐induced Tau abnormalities could disrupt ERα–estrogen binding. Molecular docking predictions, further validated by ITC, provided direct evidence of a physical interaction between Tau and ERα. Consistently, co‐immunoprecipitation and immunofluorescence assays revealed that sevoflurane markedly enhanced Tau–ERα interaction, while estrogen significantly reduced this binding. Sevoflurane not only strengthened the binding affinity between Tau and ERα but also increased the disorderliness of the interaction process. Importantly, although sevoflurane did not alter the overall Tau protein levels, it substantially elevated Tau phosphorylation, which likely accounted for the enhanced Tau–ERα association.^[^ ^48^, ^49^ ^]^ Moreover, competitive ELISA assays demonstrated that Tau and estrogen compete for ERα binding. In line with previous findings, Tau overexpression has been shown to inhibit ERα responsiveness to estrogen, presumably by sequestering ERα and preventing its activation.^[^ ^50^ ^]^ As illustrated in Figure 7H, these observations collectively suggest a mechanistic basis for sevoflurane‐induced neurotoxicity. In adult mice, relatively high levels of endogenous estrogen allow it to outcompete Tau for ERα binding, thereby preventing sevoflurane‐induced neurotoxicity. In middle‐aged mice, reduced estrogen levels weaken its binding to ERα, while sevoflurane‐enhanced Tau shows stronger affinity for ERα, thereby blocking estrogen's neuroprotective effects and promoting neurotoxicity. Supplementation with exogenous estrogen can competitively bind to ERα and restore its protective function. Importantly, ERα responsiveness to estrogen remains intact in middle‐aged mice, allowing estrogen replacement to reverse sevoflurane‐induced neurotoxic effects. In aged mice, however, physiological aging leads to ERα degradation or diminished activity,^[^ ^51^, ^52^ ^]^ thereby reducing ERα sensitivity to estrogen and effectively closing the therapeutic window. Nevertheless, upregulation of ERα expression restores estrogen's neuroprotective capacity in these aged animals.^[^ ^44^ ^]^

While this study provides valuable insights, several limitations should be acknowledged. First, although we examined the neurotoxic effects of sevoflurane in middle‐aged and aged mice, species‐specific differences in drug metabolism and neurobiology may limit the direct extrapolation of these findings to humans. Second, although sex differences in sevoflurane‐induced neurotoxicity were observed, the underlying mechanisms responsible for these differences were not further explored. Finally, the precise mechanism by which sevoflurane enhances the binding affinity between Tau and ERα remains to be clarified. In addition, the development of specific inhibitors targeting the Tau–ERα interaction would be essential to definitively establish its critical role in sevoflurane‐induced neurotoxicity. Despite these limitations, our key findings remain robust and provide a foundation for future investigations.

## 4 Conclusion

In summary, our study emphasizes the crucial role of ERα in both sevoflurane‐induced neurotoxicity and the therapeutic effects of estrogen. These findings provide a basis for developing estrogen‐based interventions to mitigate sevoflurane‐induced neurotoxicity.

## 5 Experimental Section

### Animals

C57BL/6 mice at three age stages—young adult (3–4 months), middle‐aged (15–16 months), and aged (18–19 months)—were obtained from Beijing Huafukang Biological Technology Co., Ltd. Prior to experimentation, mice were acclimated for 1 week under standard laboratory conditions (22 ± 2 °C, 50 ± 10% humidity, 12 h light/dark cycle) with ad libitum access to food and water. All procedures were approved by the Animal Welfare and Ethics Committee of Tianjin Medical University (IRB2024‐DW‐61) and conducted in accordance with institutional guidelines.

A total of 1336 mice were used in this study (excluding those that died accidentally), including 192 males (64 adult, 64 middle‐aged, and 64 aged) and 1144 females (64 adult, 616 middle‐aged, and 464 aged). Group allocation was performed using a computer‐generated randomization method. For behavioral experiments, ten mice were included per group, while six mice per group were used for Western blotting, immunofluorescence, Golgi staining, fiber photometry, and in vivo electrophysiology. To ensure ethical standards, all efforts were made to minimize the number of animals used, and euthanasia was performed using an overdose of sevoflurane to ensure humane treatment.

### Repeated Sevoflurane Exposure and Administration of Estrogen and Receptor Antagonists

Sevoflurane‐induced neurotoxicity was induced using a previously established protocol.^[^ ^53^ ^]^ Mice were placed in a sealed chamber connected to an oxygen supply and a calibrated vaporizer delivering 3% sevoflurane (Cat#S027B617, Baxter, USA) in 40% oxygen for 2 h per day over three consecutive days. Body temperature was maintained at 37.0 ± 0.5 °C with a heating pad. Control mice were exposed to 40% oxygen under identical conditions, without sevoflurane.

Doses of 17β‐estradiol and estrogen receptor antagonists were selected based on prior studies. Mice received intraperitoneal injections of 17β‐estradiol (0.2 mg kg^−1^, Cat#E8875, Sigma, USA) or vehicle 1 h before each exposure. To block receptor activity, ERα antagonist MPP (0.2 mg kg^−1^, Cat#1991, R&D, USA) or ERβ antagonist PHTPP (4 mg kg^−1^, Cat#2662, R&D, USA) was administered 30 min before estrogen treatment.

### Hippocampal AAV Injections

AAV vectors for ERα knockdown (AAV‐ERα‐K) and overexpression (AAV‐ERα‐O) were custom‐produced by Heyuan Bio (China). Viral injections were performed using the same stereotaxic procedure as electrode implantation. Mice were anesthetized and secured in a stereotaxic apparatus, and a small craniotomy was made above the hippocampus (AP −1.85 mm, ML ±1.5 mm). A total of 500 nL of virus or control vector was slowly injected at a depth of 1.30 mm. After injection, the micropipette was withdrawn, the scalp was sutured, and 1 mL of saline was administered intraperitoneally. Mice were returned to their home cages upon recovery, and experiments were initiated three weeks post‐injection.

### Western Blotting

Following anesthesia and decapitation, hippocampal tissues were rapidly isolated and homogenized in RIPA buffer containing protease and phosphatase inhibitors. The mixture was sonicated on ice and centrifuged, and the supernatant was collected. Protein concentrations were determined and adjusted to 5 µg µL^−1^. Samples were mixed with loading buffer, boiled at 100 °C for 10 min, and subjected to SDS‐PAGE, followed by transfer to PVDF membranes (Milliporre, USA). Membranes were blocked for 15 min and incubated overnight at 4 °C with primary antibodies against total Tau (1:1000, Cat#ab80579, Abcam, UK), Tau‐Ser202/Thr205 (1:1500, Cat#MN1020 Thermo Scientific, USA), Tau‐Ser396/404 (1:2000, custom‐made by Santa Cruz Biotechnology, USA), ERα (1:2000, Cat#ab32063, Abcam, UK), PSD95 (1:3000, Cat#20665‐1‐AP, Proteintech, China) and GAPDH (1:10 000, Cat#AF7021, Affinity, USA). After washing, HRP‐conjugated secondary antibodies were applied for 2 h at room temperature. Protein bands were visualized using enhanced chemiluminescence and quantified with ImageJ.

### Co‐Immunoprecipitation

Hippocampal tissues were lysed in ice‐cold lysis buffer supplemented with protease and phosphatase inhibitors, and the lysates were centrifuged at 12 000 × g for 15 min at 4 °C to obtain the supernatant. Anti‐Tau primary antibody was first incubated with Protein A/G agarose beads at 4 °C for 2 h, after which the antibody–bead complexes were incubated with the prepared lysates overnight at 4 °C. Following extensive washing, the immunocomplexes were eluted by boiling in SDS sample buffer and analyzed by western blotting with reciprocal antibodies (anti‐Tau or anti‐ERα) to detect protein–protein interactions. Normal IgG was used as a negative control to exclude nonspecific binding.

### Immunofluorescence

Mice were anesthetized and transcardially perfused with 4% paraformaldehyde (PFA). Brains were collected, post‐fixed in 4% PFA overnight at 4 °C, and dehydrated. Tissues were embedded in OCT, and sagittal sections (8 µm) were cut using a cryostat. Sections were fixed in 4% PFA for 15 min, then permeabilized and blocked in PBS containing 0.1% Triton X‐100 and 0.3% BSA for 1.5 h at room temperature. Sections were incubated overnight at 4 °C with primary antibodies against Tau‐Ser202/Thr205, 1:300, and ERα (1:200), followed by fluorescent secondary antibodies for 2 h at room temperature in the dark. Images were acquired using a fluorescence microscope (Cat# TE2000‐U, Nikon, Japan) focused on the dorsal hippocampal CA1 (dCA1) region, and the number of positive cells was quantified using ImageJ software.

### Morris Water Maze

Cognitive function was evaluated using the Morris water maze three weeks after modeling. The test was conducted in a circular pool (120 cm diameter, 50 cm height) filled with opaque water using non‐toxic white dye. A transparent platform (10 cm diameter) was submerged 2 cm below the surface in the second quadrant. Water temperature, platform location, and visual cues were kept constant. Mice underwent four training trials per day for five consecutive days, starting from different quadrants and searching for the platform for up to 90 s. If unsuccessful, mice were guided to the platform and allowed to remain for 15 s; if successful, escape latency was recorded. On the fifth day, the platform was removed for a 90‐s probe test. Mice were placed in the opposite quadrant, and the number of platform crossings and time spent in the target quadrant were recorded to assess spatial memory.

### Y‐Maze

A Y‐maze with three opaque arms (start, novel, and familiar), each 30 cm long and 5 cm wide, was used to assess spatial recognition memory. The maze was placed in a quiet, temperature‐controlled room with uniform lighting, and mice were acclimated to the environment for one week. During training, the novel arm was blocked, and mice explored the start and familiar arms freely for 15 min. 1 h later, in the testing phase, the novel arm was opened, and mice were allowed to explore all three arms for 10 min. The number of entries into the novel arm and the time spent there were recorded to assess exploratory behavior and memory performance.

### Golgi Staining

Golgi staining was used to assess dendritic spine density in hippocampal neurons. Mouse brains were immersed in AB solution (prepared 24 h in advance, FD NeuroTechnologies, USA) for 48 h, then in solution C for 5 days, all at room temperature in the dark. Brains were sectioned into 110 µm slices using a vibrating microtome and mounted on slides. Sections were washed, stained with a D/E/water mixture (1:1:2), dehydrated through graded ethanol, cleared in xylene, and sealed with neutral resin. The dCA1 region was identified under low magnification, and dendritic spine density was examined under 100× oil immersion. Images were analyzed using ImageJ, and dendritic spines were classified based on previous studies.^[^ ^48^, ^54^ ^]^

### In Vivo Optical Fiber Photometry for Ca^2+^ Recording

Fiber photometry was performed as previously described.^[^ ^12^ ^]^ Briefly, mice were anesthetized and secured in a stereotaxic frame for virus injection and optical fiber implantation. After exposing the skull, a small craniotomy was made above the dCA1 region (AP − 1.85 mm, ML + 1.5 mm), and 100–200 nL of pAAV‐Syn‐GCaMP6f (Heyuan Bio, China) was injected at a depth of 1.27 mm. An optical fiber cannula (Shanghai Fiblaser, China) was then inserted to 1.25 mm and fixed with dental cement. After recovery, fiber photometry was used to monitor Ca^2^⁺ signals in dCA1 during the Y‐maze test. Mice explored the start and familiar arms for 15 min (training phase), and 1 h later, all arms were opened for a 10‐min test phase. Ca^2^⁺ signals were recorded throughout, with behavioral events (e.g., entry into the novel arm) marked for analysis. Signal data were extracted and visualized using MATLAB, followed by statistical analysis.

### In Vivo Electrophysiology

The implantation of in vivo electrophysiological electrodes was similar to fiber photometry. A 4 × 4 tungsten microelectrode (Bio‐signal, China) was inserted into the same dCA1 location and secured with dental cement. Following recovery, neuronal activity was recorded during the novel object recognition test. The novel object recognition test was carried out in a transparent open‐field box (40 × 40 × 40 cm) with a replaceable white paper floor. One day prior to testing, mice were habituated by exploring the empty arena for 10 min. During the training phase, two identical objects (A1 and A2) were placed in opposite corners for a 10‐min exploration period. 1 h later, one familiar object was replaced with a novel object (B), and mice were allowed to explore for 5 min. The time of exploration toward the novel object was recorded for analysis. Raw data were processed with Offline Sorter to isolate single‐unit activity, and event‐related firing rates were visualized and analyzed using NeuroExplorer for subsequent data analysis.

### Molecular Docking Prediction

To investigate the potential interaction between ERα and Tau, we first retrieved experimentally validated 3D structures of human ERα and Tau proteins from the UniProt (Universal Protein Resource) database. Protein–protein docking was then performed using GRAMM docking software, with parameter optimization to improve the accuracy of the predicted interaction and to refine the docking conformations. The resulting complexes were evaluated using the PDBePISA scoring system to analyze the binding interface and assess the stability of the protein–protein interaction. Key parameters such as interface area, hydrogen bonding, hydrophobic interactions, and energy contributions were examined to identify high‐affinity binding conformations. Molecular visualization and structural analysis were conducted using PyMOL.

### Competitive ELISA

Following previously established protocols,^[^ ^55^ ^]^ high‐binding 96‐well plates were coated with recombinant ERα (2 µg mL^−1^) in 0.05 mol L^−1^ carbonate buffer and incubated overnight at 4 °C. After washing with 1× PBS, the plates were blocked with 3% BSA at 37 °C for 1.5 h, washed again, air‐dried, and stored at 4 °C until use. For detection, 100 µL of His‐tagged Tau protein (0.5 µg mL^−1^) was mixed with 100 µL of standard estrogen solutions (ranging from 0.00001 to 100 µg mL^−1^) and added to the coated wells, followed by 1‐h incubation at 37 °C. After PBS washing, anti‐His‐HRP conjugate and TMB substrate were applied for color development, and absorbance was measured at 450 nm using a microplate reader.

### Tau Protein Isolation and Purification

Mice were euthanized, and hippocampal tissues were homogenized in five volumes of 37 °C reassembly (RA) buffer (0.5 mm MgSO_4_, 2 mm GTP, 1 mm EGTA, 2 mm DTT, 20 µm paclitaxel, 0.1% Triton X‐100, 1 mm PMSF, 1 mm Na_3_VO_4_, 1 mm NaF, protease inhibitors, pH 6.5). After sonication and centrifugation (3000 × g, 2 min), the supernatant was ultracentrifuged at 100000 × g for 20 min at 25 °C to obtain soluble (non‐microtubule‐bound) Tau. The supernatant was buffer‐exchanged into 20 mm HEPES, 50 mm NaCl (pH 7.4), purified by cation exchange chromatography (HiTrap SP HP column), and eluted with a NaCl gradient (50 mm–1 m). Tau‐containing fractions were further purified by size exclusion chromatography (Superdex 75) in 50 mm HEPES, 150 mm NaCl (pH 7.4), concentrated, quantified, and stored at −80 °C for subsequent experiments.

### Isothermal Titration Calorimetry

The isothermal titration calorimetry (ITC) experiment was performed as previously described.^[^ ^56^ ^]^ Briefly, ERα and Tau proteins were dissolved in their respective buffers and diluted to the desired concentrations. All solutions were degassed at 20 °C under a vacuum pressure exceeding 400 mmHg for 10 min, and the buffer pH and ionic strength were adjusted to appropriate levels. The solutions were then filtered through a 0.22 µm membrane to remove particulates. A volume of 300 µL ERα solution was loaded into the sample cell, ensuring no air bubbles remained. Simultaneously, 50 µL of Tau protein solution was loaded into the syringe and also degassed for 10 min. After initiating the ITC system, Tau protein was titrated into the sample cell in 20 injections of 2 µL each, with 120‐s intervals between injections. The titration was conducted at 25 °C with a stirring speed of 350 rpm. Heat flow data were analyzed using the appropriate software. To correct for the heat of dilution, a control titration was conducted by injecting the same Tau protein solution into buffer alone.

### Statistical Analysis

All data were analyzed using SPSS (Version 22.0) and visualized with GraphPad Prism (Version 8.02). Data normality was assessed using the Shapiro–Wilk test. For normally distributed data, values are expressed as mean ± standard deviation (SD) for two‐group comparisons and as mean ± standard error of the mean (SEM) for multiple‐group comparisons. For non‐normally distributed data, values were reported as median and interquartile range (IQR). For normally distributed data, two‐group comparisons were performed using independent‐samples *t*‐tests, while comparisons among multiple groups were conducted using one‐way ANOVA followed by Bonferroni post hoc corrections. For non‐normally distributed data, two‐group comparisons were analyzed using the Mann–Whitney U test, and multiple‐group comparisons were performed using the Kruskal–Wallis test followed by Dunn's post hoc test. *, **, ***, and ns indicate *p* < 0.05, *p* < 0.01, *p* < 0.001, and not significant (*p* > 0.05), respectively.

### Ethics Approval Statement

All procedures were approved by the Animal Welfare and Ethics Committee of Tianjin Medical University (IRB2024‐DW‐61) and conducted in accordance with institutional guidelines.

## Conflict of Interest

The authors declare no conflict of interest.

## Supporting information



Supporting Information

Supporting Information

## Data Availability

The data that support the findings of this study are available from the corresponding author upon reasonable request.
