# Supplementary material for: Estrogen Alleviates Sevoflurane‐Induced Neurotoxicity by Inhibiting ERα‐Tau Binding
Source: Adv Sci (Weinh). 2025 Sep 6;12(45):e08568. doi: 10.1002/advs.202508568 (PMC12677654; doi:10.1002/advs.202508568)
Supplement: Supplementary file 1 — Supporting Information [file ADVS-12-e08568-s002.docx]

**Supplementary materials**

**Supplementary Legends**

**Supplementary Figure S1.** Effects of sevoflurane on cognitive function in male and female mice across different age groups. (A, B) Representative tracking trajectories in the Y-maze task for male (A) and female (B) mice at different ages. (C, D) Quantification of time spent exploring the novel arm (C) and number of entries into the novel arm (D) in male and female mice across different age groups following sevoflurane exposure (n = 10/group). Data in (C) and (D) are presented as mean ± SD. Statistical analysis: Unpaired t-test was used for (C) and (D). ns, no significance; **P*<0.05; ***P*<0.01.

**Supplementary Figure S2.** Hippocampal estrogen levels across different experimental groups. (A) Estrogen levels in the hippocampus of female mice of different age groups (n = 6 per group). (B) Quantification of hippocampal estrogen concentrations in female mice of varying age groups and treatment conditions (n = 6 per group). Data in (A) and (B) are presented as mean ± SEM. Statistical analysis: One-way ANOVA with Bonferroni post hoc test was used for (A). Two-way ANOVA with Bonferroni post hoc test. ****P*<0.001.

**Supplementary Figure S3.** Effects of estrogen and sevoflurane on cognitive function in the hippocampus of middle-aged and aged female mice. (A) and (D) Representative swimming trajectories in the Morris water maze task for middle-aged (A) and aged (D) female mice. (B, C) Latency to locate the hidden platform and number of platform crossings in the Morris water maze task for middle-aged female mice (n = 10 per group). (E, F) Latency to locate the hidden platform and number of platform crossings in the Morris water maze task for middle-aged female mice (n = 10 per group). (G, H) Representative Western blot image (G) and corresponding quantification (H) of hippocampal PSD95 expression in middle-aged mice treated with sevoflurane and estrogen (n=6 per group). (I, J) Representative Western blot image (I) and corresponding quantification (J) of hippocampal PSD95 expression in aged mice treated with sevoflurane and estrogen (n=6 per group). Data in (B), (E), (H) and (J)are presented as mean ± SEM; data in (C) and (F) are presented as median and IQR. Statistical analysis: Repeated measures ANOVA was applied to (B) and (E); Kruskal-Wallis test with Dunn’s post hoc was used for (C) and (F); One-way ANOVA with Bonferroni post hoc test was used for (H) and (J). Abbreviations: Estrogen (Estr); Sevoflurane (Sevo). ns, no significance; ***P*<0.01; ****P*<0.001; ^#^*P*<0.05; ^##^*P*<0.01; ^###^*P*<0.001.

**Supplementary Figure S4.** Effects of estrogen and sevoflurane on neuronal firing in dCA1 neurons of middle-aged and aged female mice. (A) and (B) Representative in vivo electrophysiological recordings of clustered neurons in the dorsal CA1 region of middle-aged (A) and aged (B) female mice. (C) and (D) respectively show the effects of estrogen and sevoflurane on mushroom-type dendritic spines in the dCA1 region of middle-aged (C) and aged (D) female mice (n=6 per group). Data in (C) and (D) are presented as mean ± SEM. Statistical analysis: One-way ANOVA with Bonferroni post hoc test was used for (C) and (D). ns, no significance; ***P*<0.01; ****P*<0.001.

**Supplementary Figure S5.** Effects of estrogen combined with receptor antagonists and sevoflurane on cognitive function in middle-aged and aged female mice. (A) and (B) Representative tracking trajectories of middle-aged female mice in the Morris water maze (A) and Y-maze (B) tasks. (C, D) Representative Western blot image (C) and corresponding quantification (D) of hippocampal PSD95 expression in middle-aged mice treated with estrogen, receptor antagonists, and sevoflurane (n=6 per group). (E) The effects of estrogen combined with receptor antagonists and sevoflurane on mushroom-type dendritic spines in the dCA1 region of middle-aged female mice (n=6 per group). Data in (D) and (E) are presented as mean ± SEM. Statistical analysis: One-way ANOVA with Bonferroni post hoc test was used for (D) and (E). Abbreviations: Estrogen (Estr); Sevoflurane (Sevo). ns, no significance; **P*<0.05; ***P*<0.01.

**Supplementary Figure S6.** Effects of estrogen combined with viral injection on hippocampal ERα expression and Tau phosphorylation in female mice. (A) and (B) Representative Western blot image (A) and corresponding quantification (B) of hippocampal ERα protein expression in female mice of different age groups (n = 6 per group). (C) and (D) Representative Western blot image (C) and corresponding quantification (D) of hippocampal ERα protein expression in middle-aged female mice treated with AAV-ERα-K (n = 6 per group). (E) and (F) Representative Western blot image (E) and corresponding quantification (F) of hippocampal ERα protein expression in aged female mice treated with AAV-ERα-O (n = 6 per group). (G) and (H) Representative Western blot image (G) and corresponding quantification (H) of ERα protein expression in the hippocampus of middle-aged female mice (n = 6 per group). (I) and (J) Representative Western blot image (I) and corresponding quantification (J) of ERα protein expression in the hippocampus of aged female mice (n = 6 per group). (K) Quantification of ERα co-localization with pyramidal neurons in the hippocampus of middle-aged mice via immunofluorescence (n = 6 per group). (L) Quantification of ERα co-localization with pyramidal neurons in the hippocampus of aged mice via immunofluorescence (n = 6 per group). (M,N) Western blot quantification of Tau-Ser202/Thr205 (M) and Tau-Ser396/404 (N) in the hippocampus of middle-aged female mice (n=6 per group). (O, P) Western blot quantification of Tau-Ser202/Thr205 (O) and Tau-Ser396/404 (P) in the hippocampus of middle-aged female mice (n=6 per group). Data in (B), (H), (J), (K), (L), (M), (N), (O) and (P) are presented as mean ± SEM. Data in (D) and (F) are presented as mean ± SD. Statistical analysis: One-way ANOVA with Bonferroni post hoc test was used for (B), (H), (J), (K), (L), (M), (N), (O) and (P). Unpaired t-test was used for (D) and (F). ns, no significance; **P*<0.05; ***P*<0.01; ****P*<0.001.

**Supplementary Figure S7.** Effects of estrogen combined with viral injection and sevoflurane on Tau phosphorylation and cognitive function in the hippocampus of middle-aged and aged female mice. (A, B) Representative immunofluorescence images (A) and quantification of Tau-Ser202/Thr205 (B) in the CA1 region of the hippocampus in middle-aged female mice (indicated by white arrows, n = 6 per group). (C, D) Representative immunofluorescence images (C) and quantification of Tau-Ser202/Thr205 (D) in the CA1 region of the hippocampus in aged female mice (indicated by white arrows, n = 6 per group). (E) and (G) Representative tracking trajectories of middle-aged female mice in the Morris water maze (E) and Y-maze (G) tasks. (F) and (H) Representative tracking trajectories of middle-aged female mice in the Morris water maze (F) and Y-maze (H) tasks. (I, J) Representative Western blot image (I) and corresponding quantification (J) of hippocampal PSD95 expression in middle-aged mice following combined treatment with estrogen, viral injection, and sevoflurane (n=6 per group). (K, L) Representative Western blot image (K) and corresponding quantification (L) of hippocampal PSD95 expression in aged mice following combined treatment with estrogen, viral injection, and sevoflurane (n=6 per group). Data in (A), (C), (J) and (L) are presented as mean ± SEM. Statistical analysis: One-way ANOVA with Bonferroni post hoc test was used for (A), (C), (J) and (L). Abbreviations: Estrogen (Estr); Sevoflurane (Sevo). ns, no significance; ***P*<0.01.

**Supplementary Figure S8.** Effects of estrogen combined with viral injection on neuronal firing in dCA1 neurons of middle-aged and aged female mice. (A) and (B) Representative in vivo electrophysiological recordings of clustered neurons in the dorsal CA1 region of middle-aged (A) and aged (B) female mice. (C) and (D) show the effects of estrogen combined with viral injection on mushroom-type dendritic spines in the dCA1 region of middle-aged (C) and aged (D) female mice (n=6 per group). Data in (C) and (D) are presented as mean ± SEM. Statistical analysis: One-way ANOVA with Bonferroni post hoc test was used for (C) and (D). ns, no significance; **P*<0.05; ***P*<0.01

**Supplementary Figures**


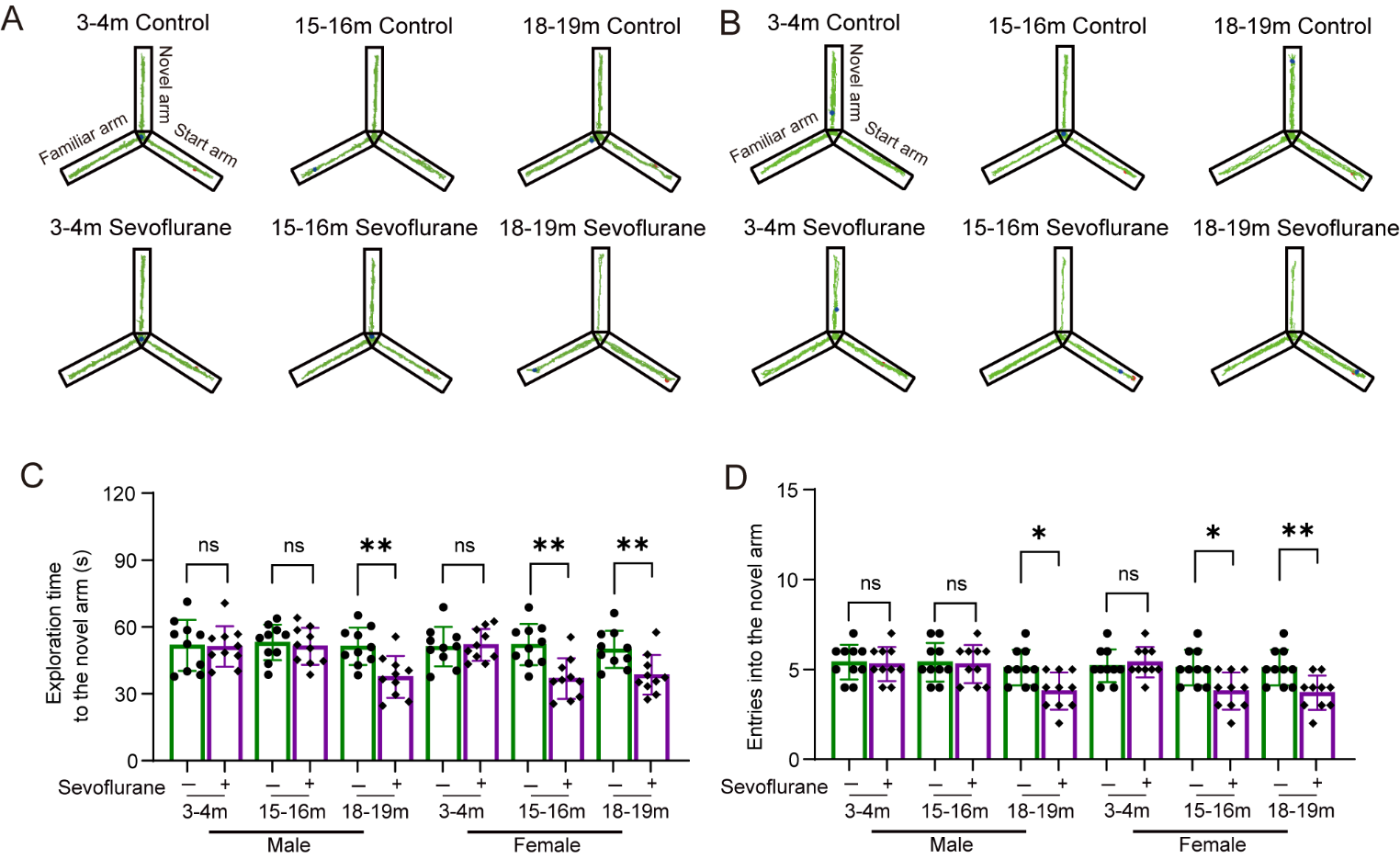


**Supplementary Figure S1**


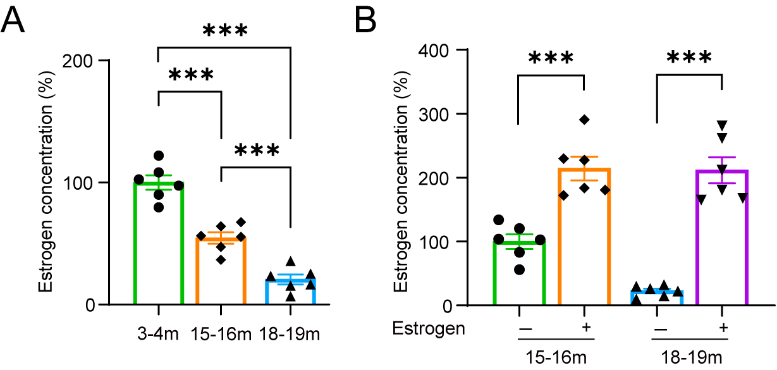


**Supplementary Figure S2**


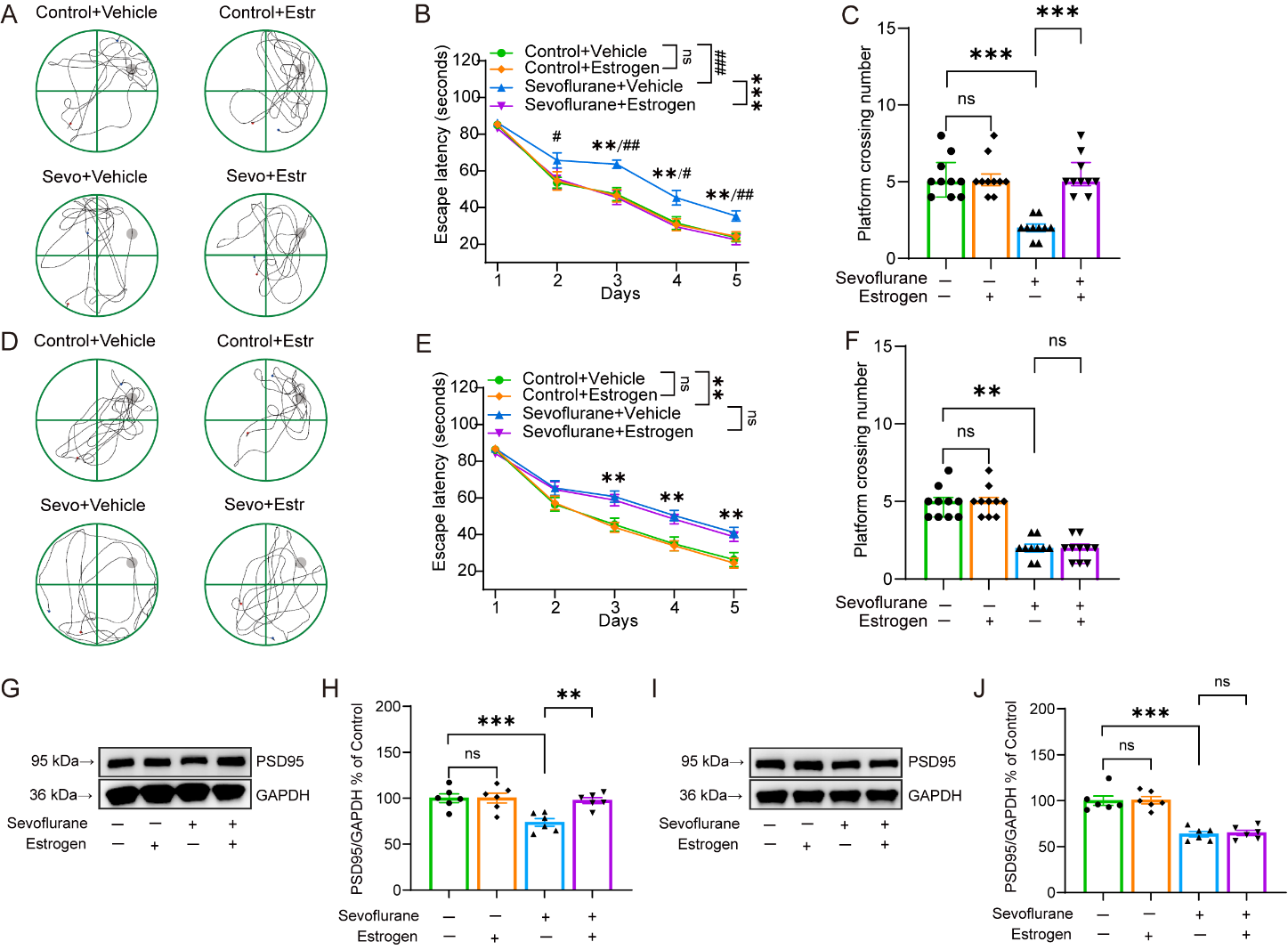


**Supplementary Figure S3**


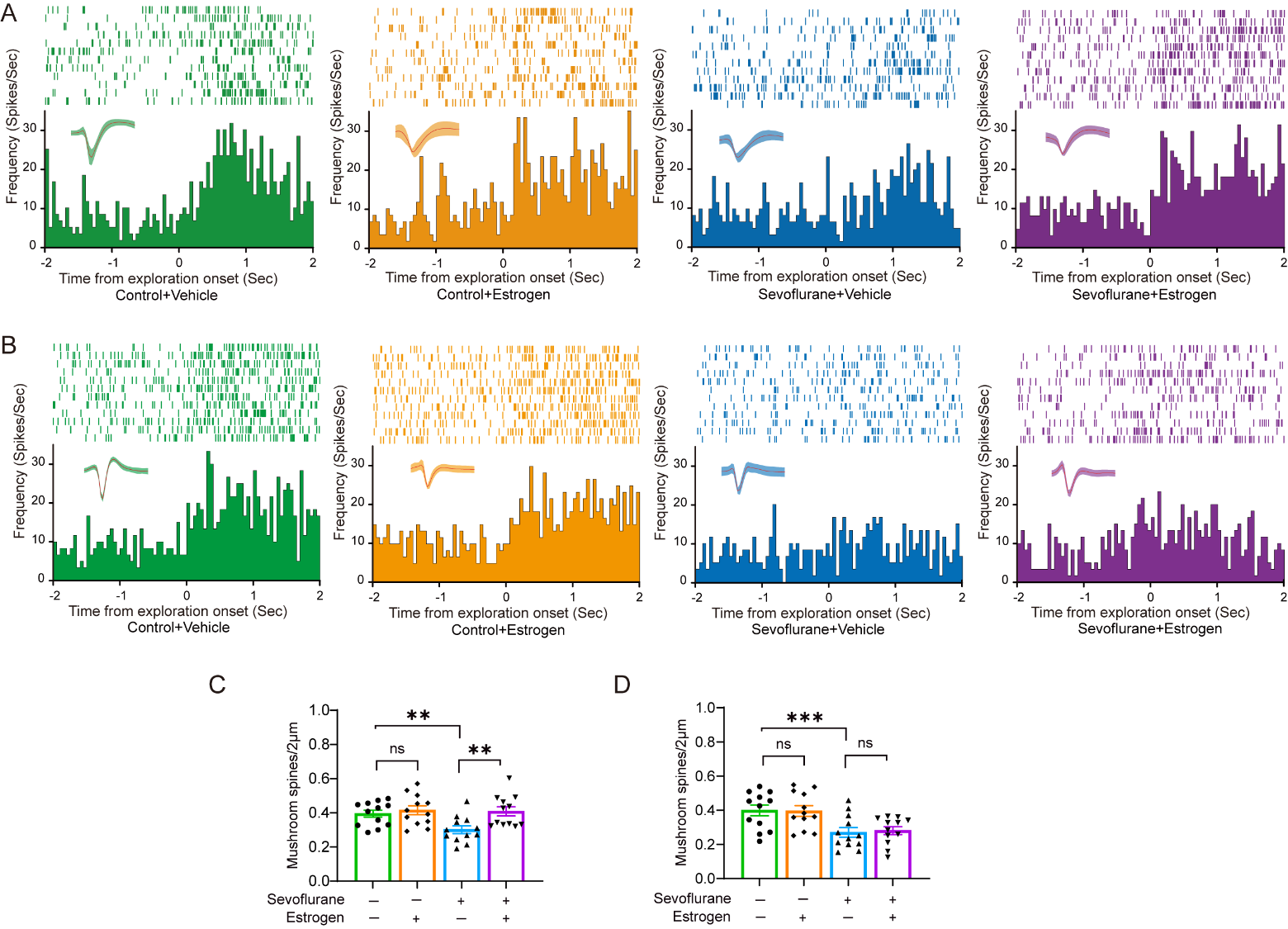


**Supplementary Figure S4**


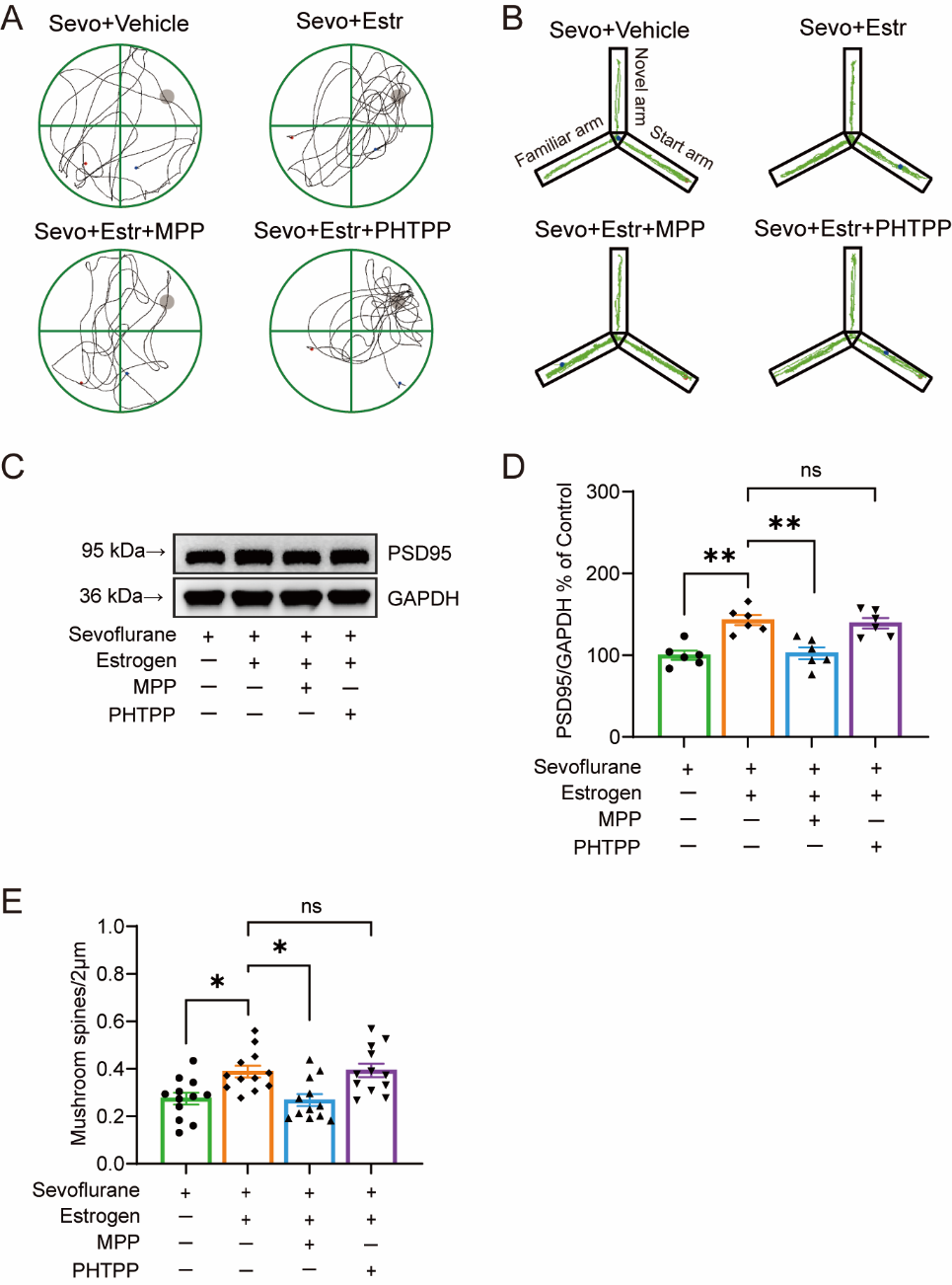


**Supplementary Figure S5**


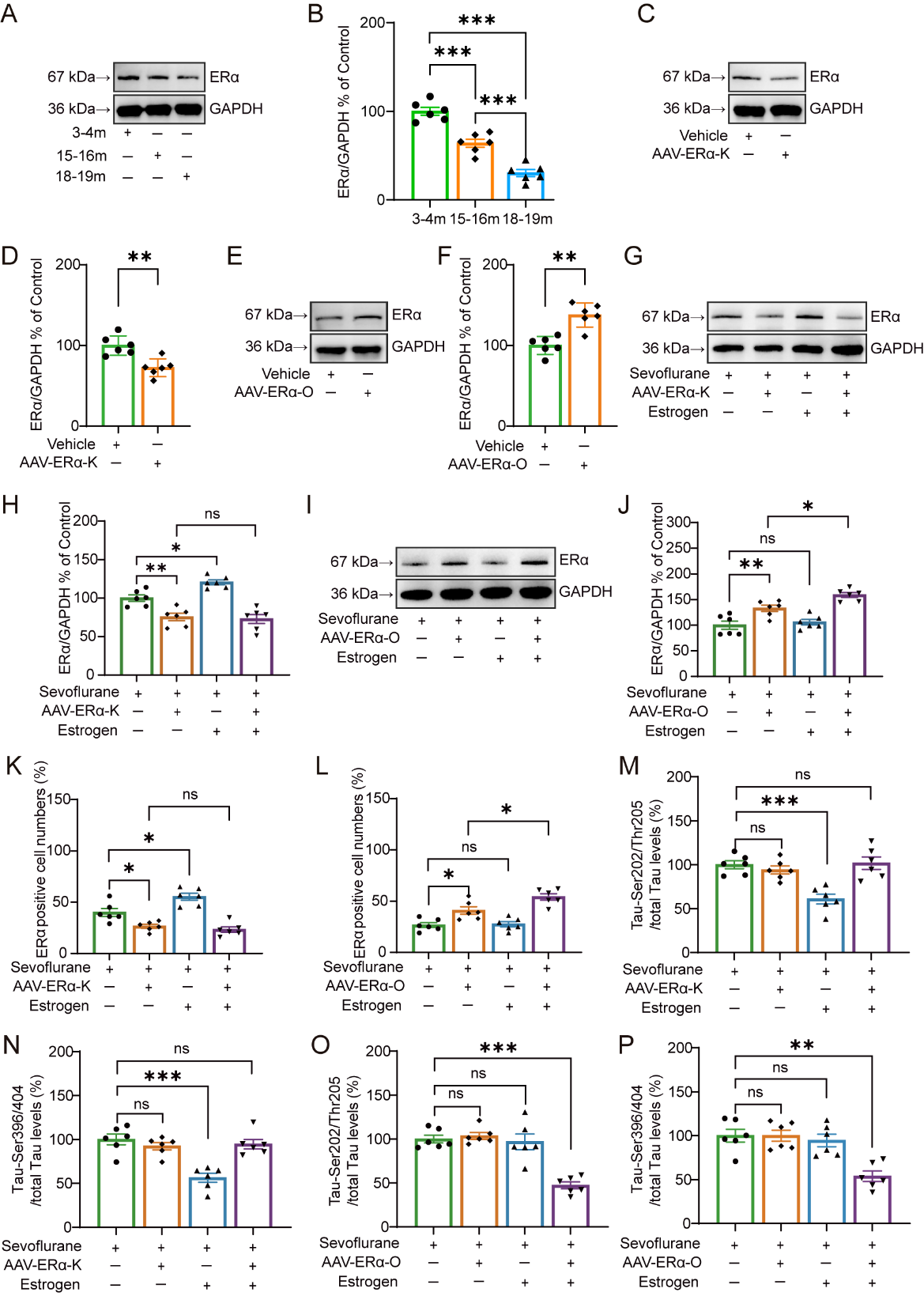


**Supplementary Figure S6**


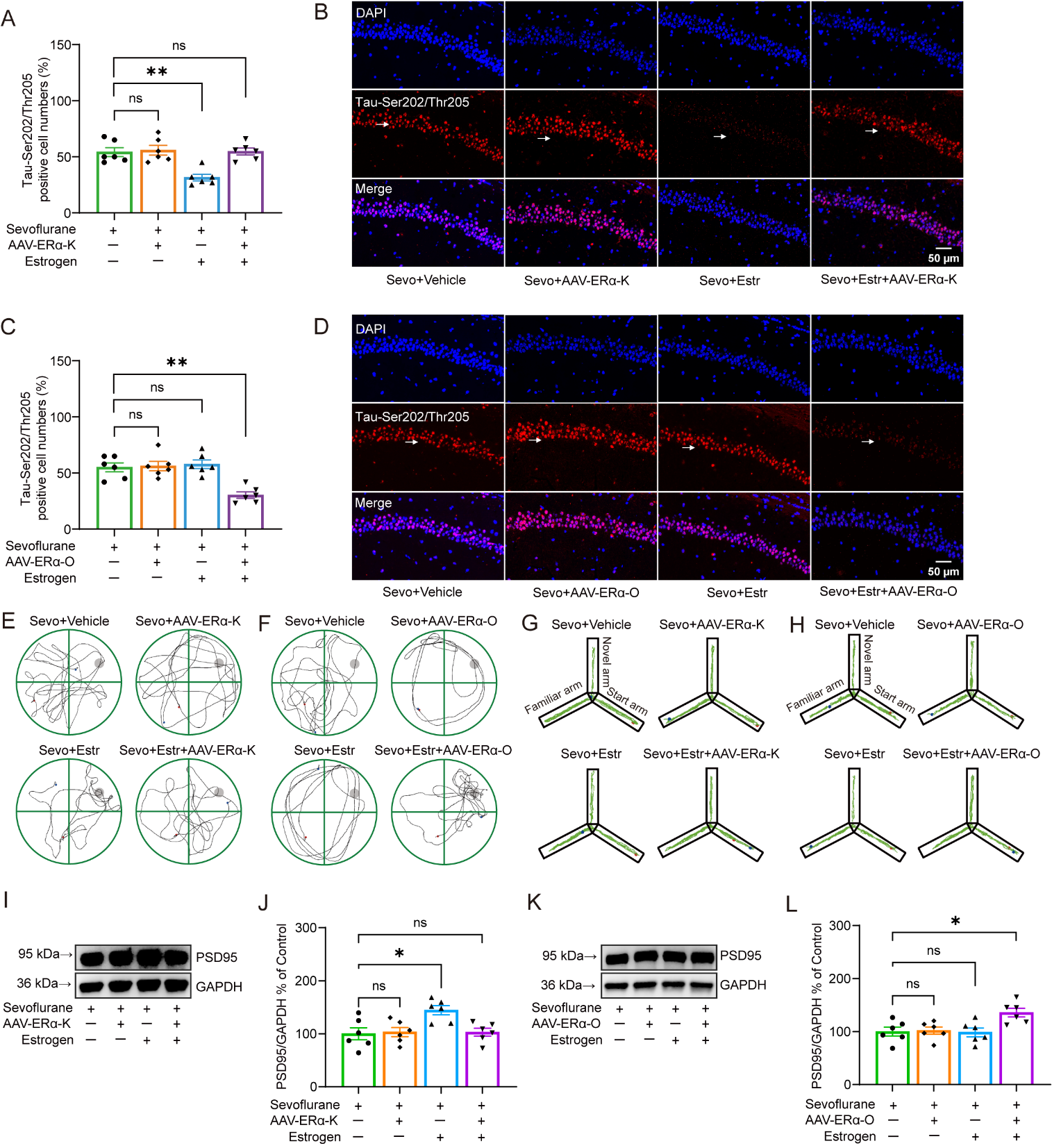


**Supplementary Figure S7**


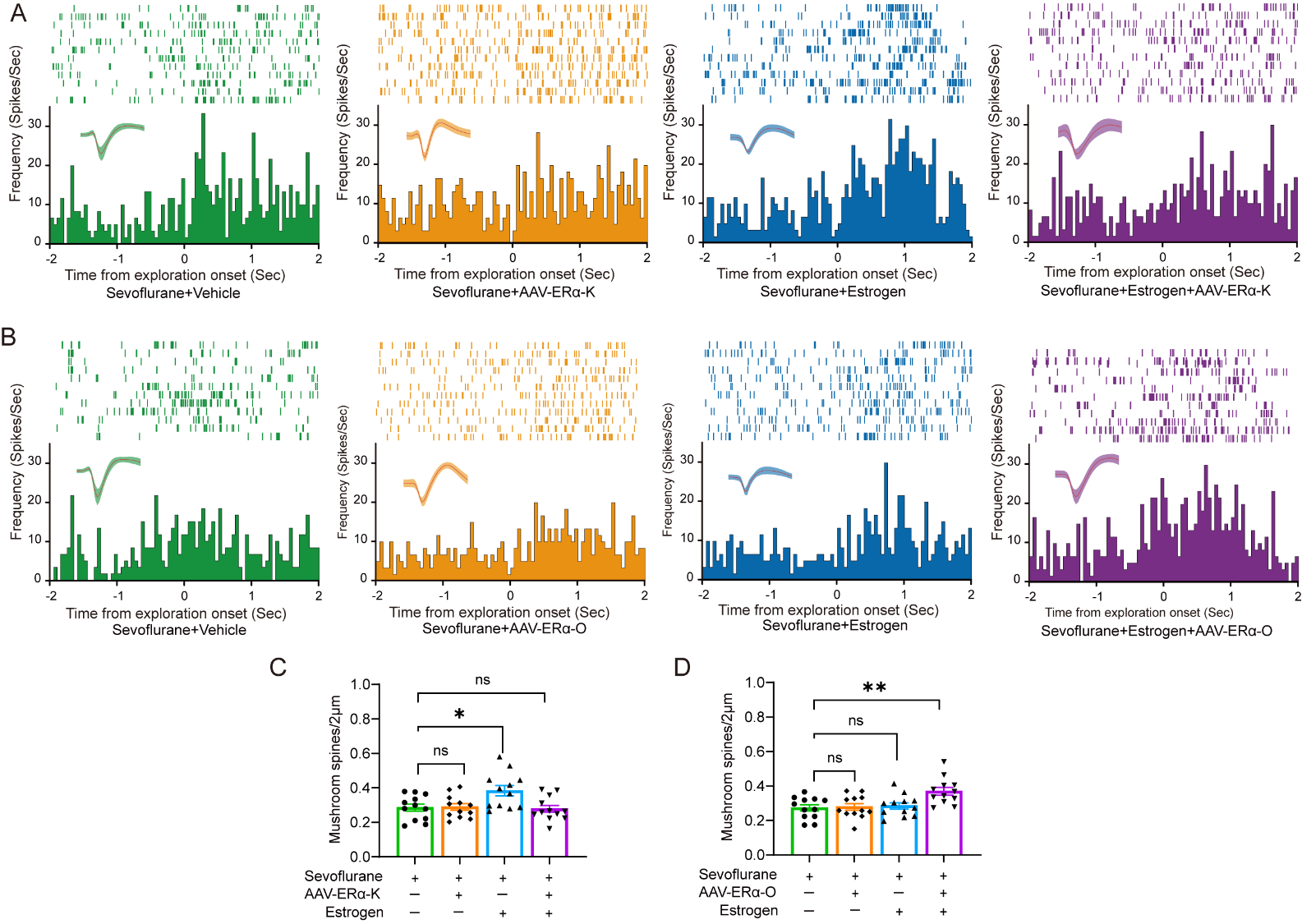


**Supplementary Figure S8**
