# Supplementary material for: Estrogen Alleviates Sevoflurane‐Induced Neurotoxicity by Inhibiting ERα‐Tau Binding
Source: Adv Sci (Weinh). 2025 Sep 6;12(45):e08568. doi: 10.1002/advs.202508568 (PMC12677654; doi:10.1002/advs.202508568)

The original image analyzed by Sholl in Figure 3 J is as follows:


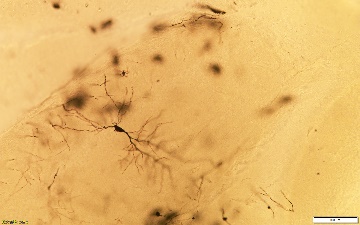

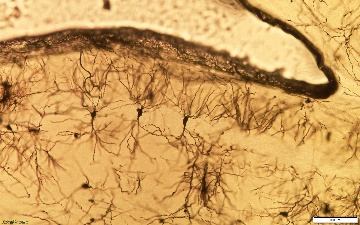

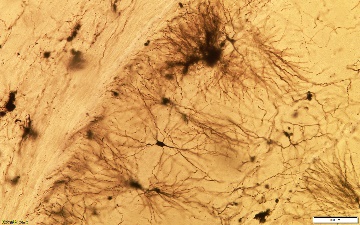

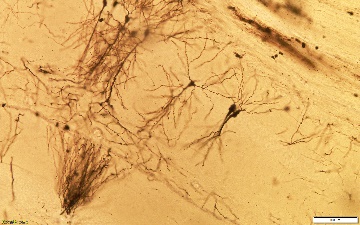


The original image analyzed by Sholl in Figure 3 M is as follows:


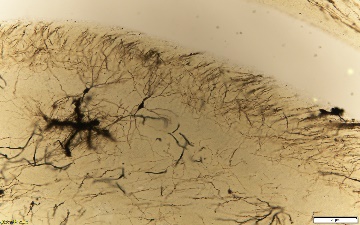

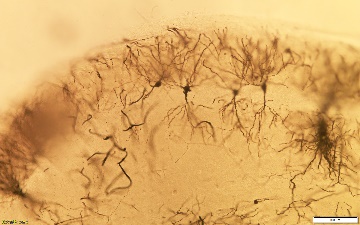

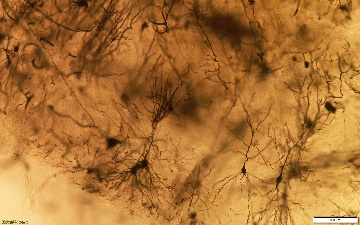

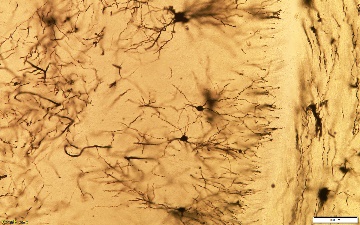


The original image analyzed by Sholl in Figure 4 K is as follows:


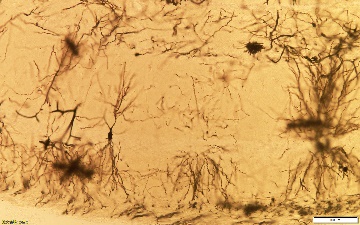

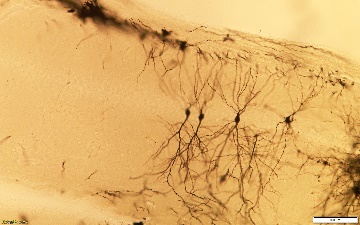

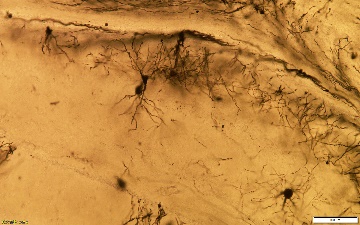

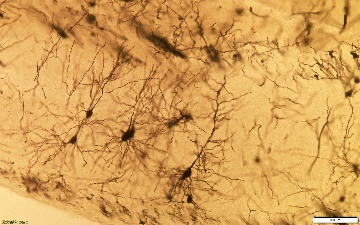


The original image analyzed by Sholl in Figure 6 J is as follows:


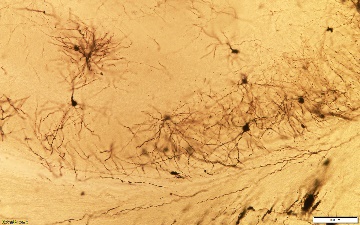

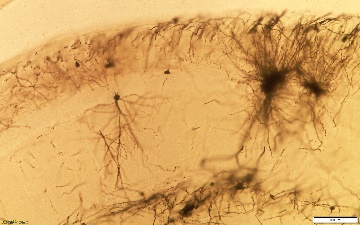

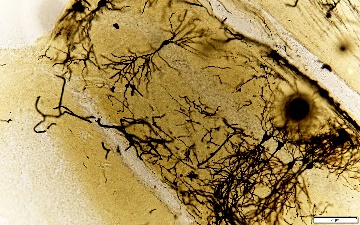

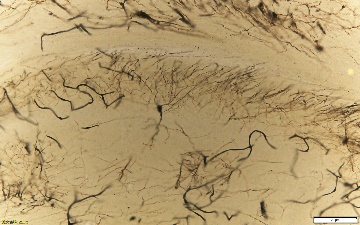


The original image analyzed by Sholl in Figure 6 M is as follows:


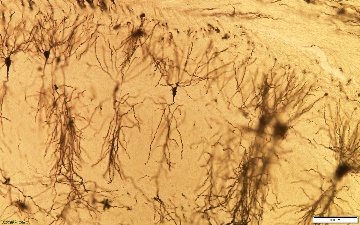

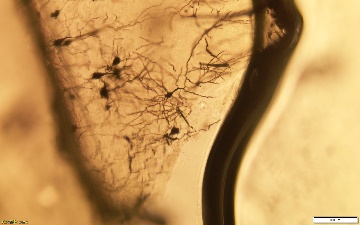

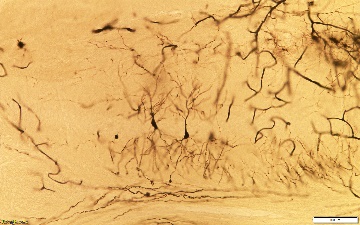

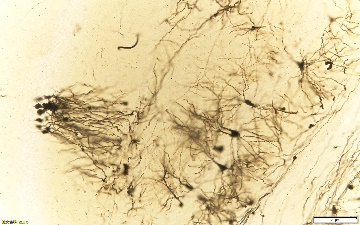

Supplement: Supplementary file 2 — Supporting Information [file ADVS-12-e08568-s001.zip › sholl .docx]
