# Supplementary material for: Estrogen Alleviates Sevoflurane‐Induced Neurotoxicity by Inhibiting ERα‐Tau Binding
Source: Adv Sci (Weinh). 2025 Sep 6;12(45):e08568. doi: 10.1002/advs.202508568 (PMC12677654; doi:10.1002/advs.202508568)
Supplement: Supplementary file 2 — Supporting Information [file ADVS-12-e08568-s001.zip › WB Figure.docx]

The chemiluminescent pre-stained protein marker reagent from Biosharp, catalog number BL1106A, has KDAs labeled from bottom to top as 22, 28, 38, 50, 62,78 and 113. The specific instructions can be found on the official website: <http://www.jinpanlab.com/archives/109636>.

Figure 1 B


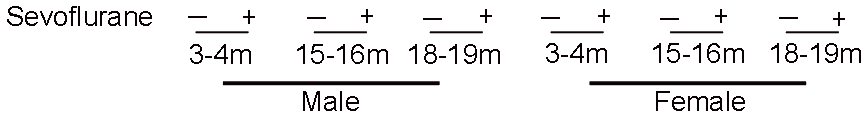

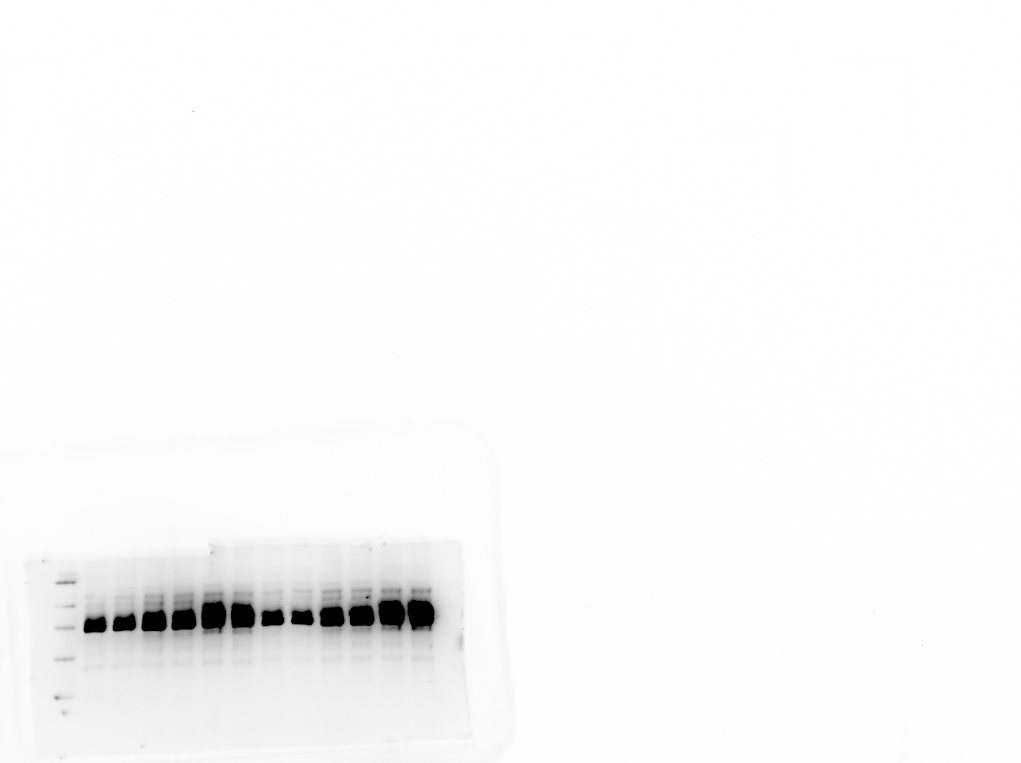


total Tau

50kDa

38kDa

28kDa

22kDa


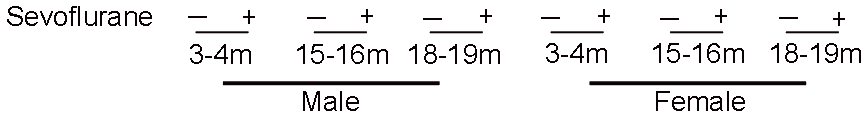

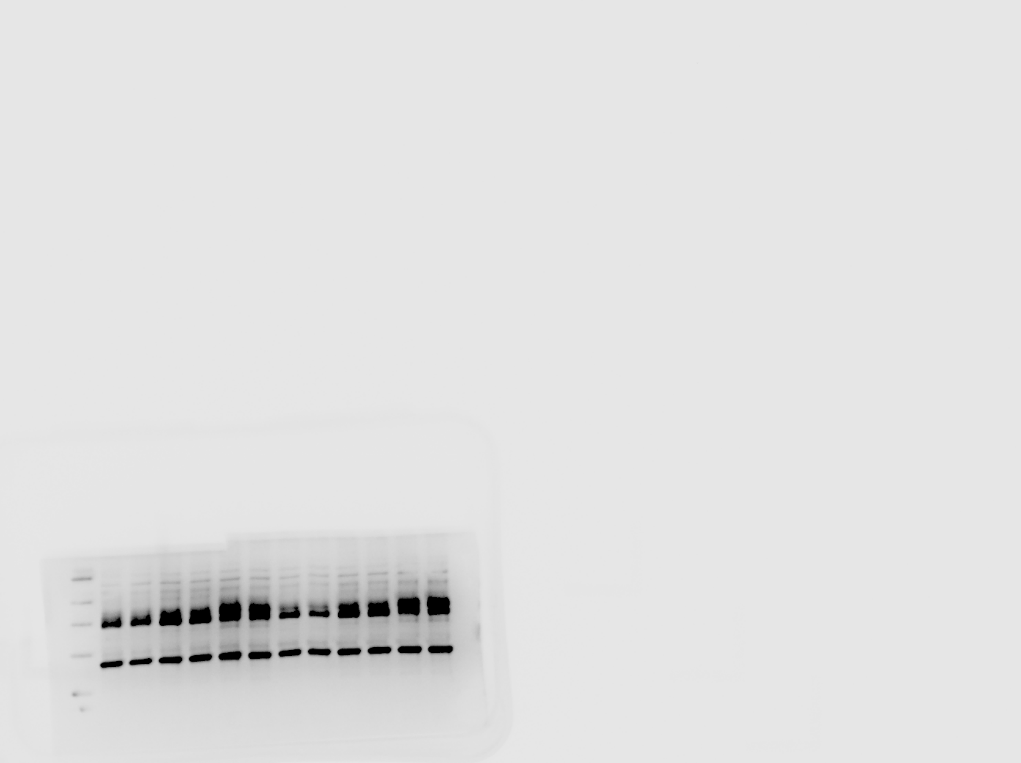


50kDa

38kDa

28kDa

22kDa

GAPDH

Full unedited blot for Figure 1B total Tau and GAPDH


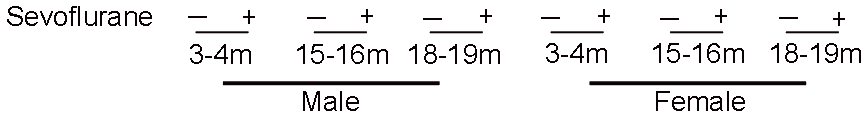

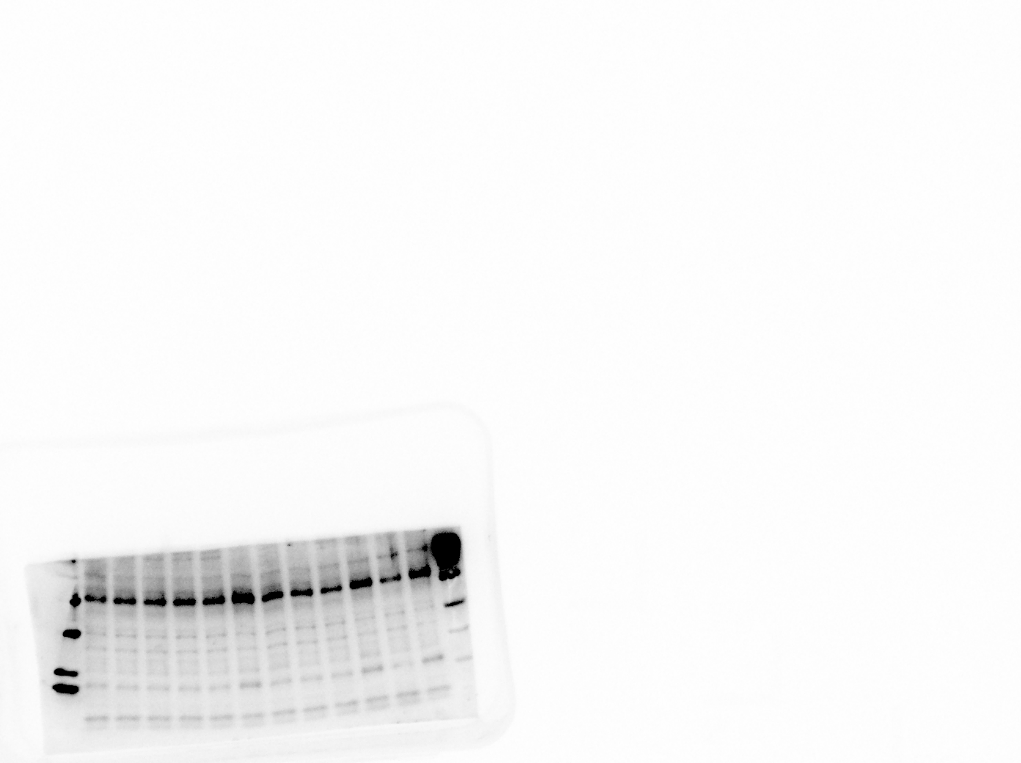


P-Tau-Ser202/Thr205

50kDa

38kDa

28kDa

22kDa


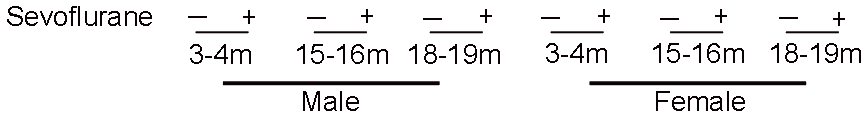

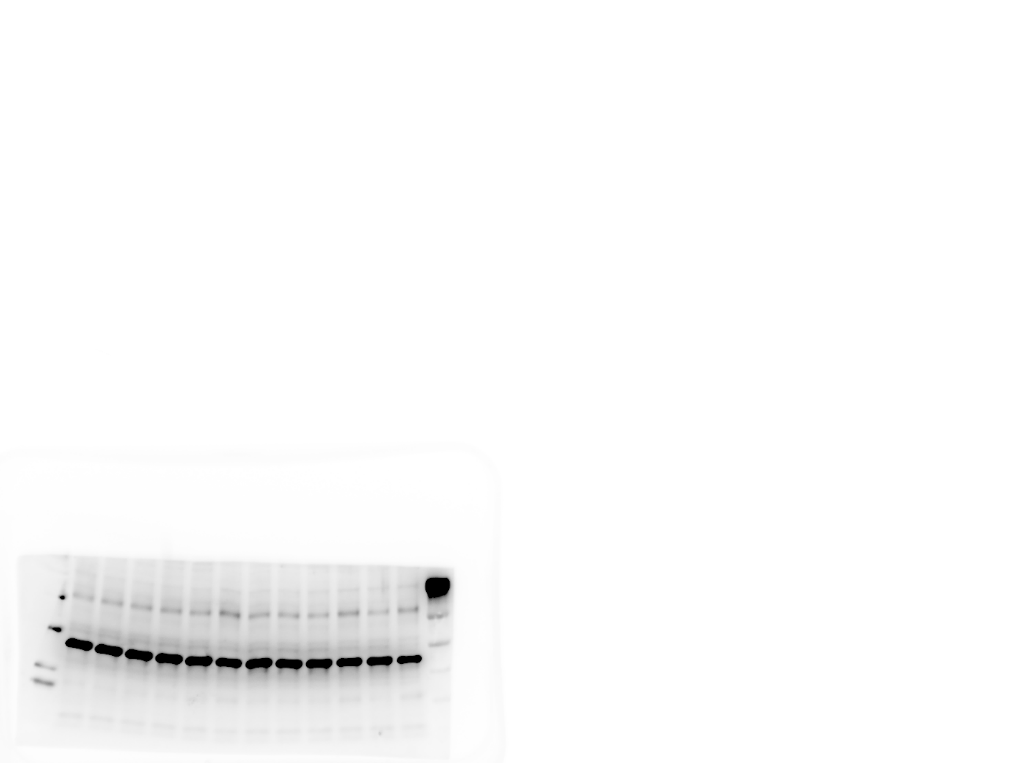


50kDa

38kDa

28kDa

22kDa

GAPDH

Full unedited blot for Figure 1B P-Tau-Ser202/Thr205 and GAPDH


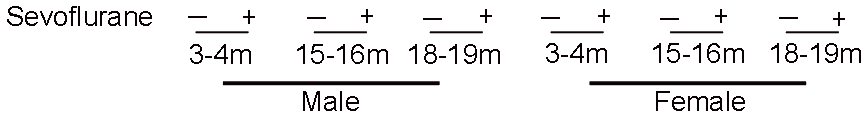

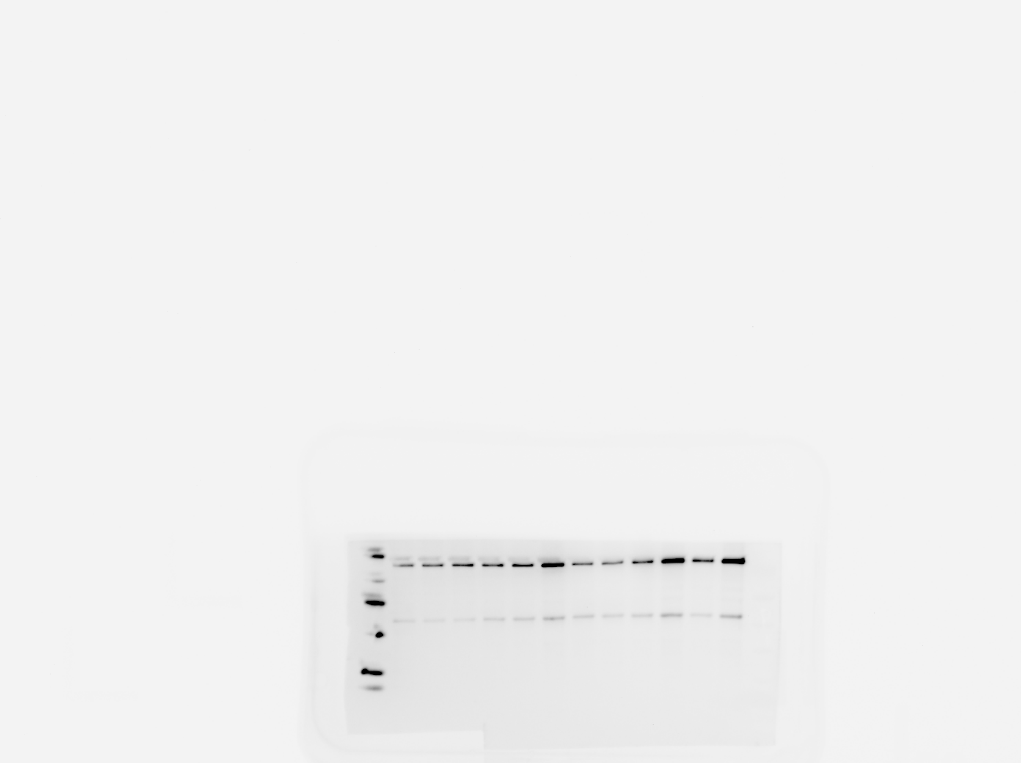


P-Tau-Ser396/404

total Tau

62kDa

50kDa

38kDa

28kDa

22kDa

50kDa

38kDa

28kDa

22kDa


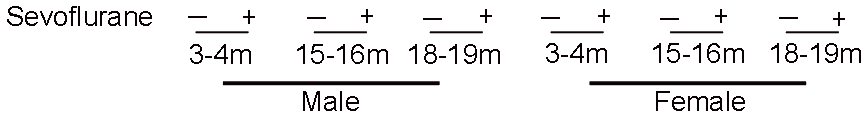

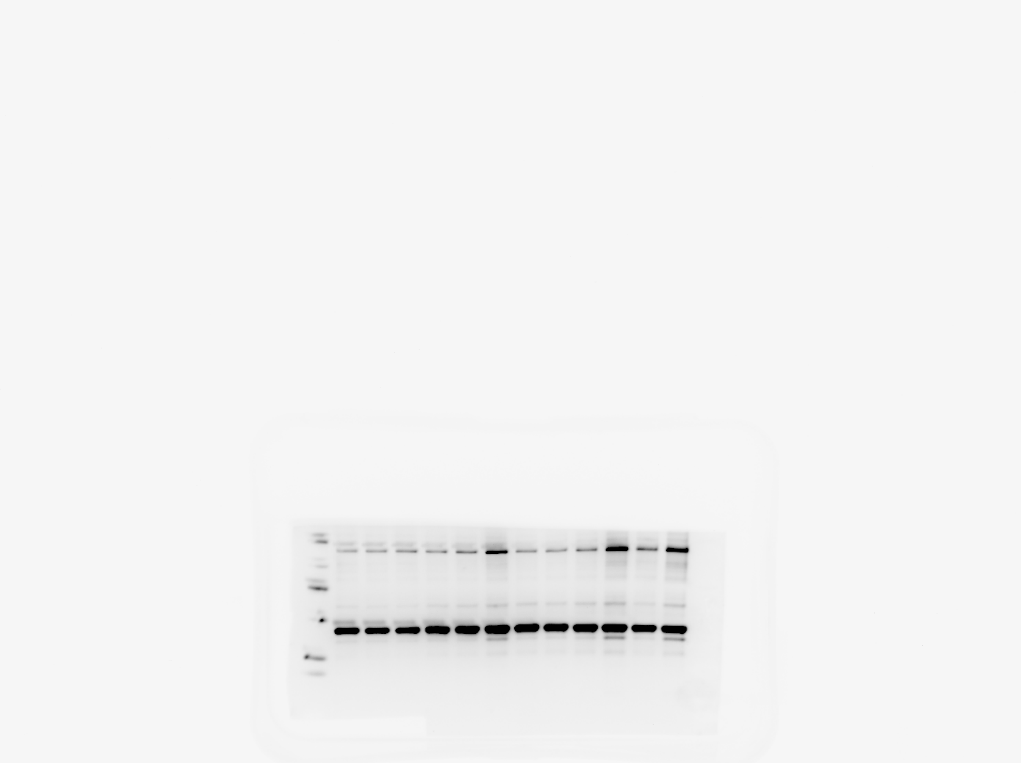


62kDa

50kDa

38kDa

28kDa

22kDa

GAPDH

Full unedited blot for Figure 1B P-Tau-Ser396/404 and GAPDH

Figure 2B


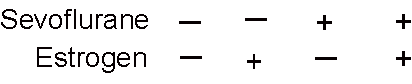

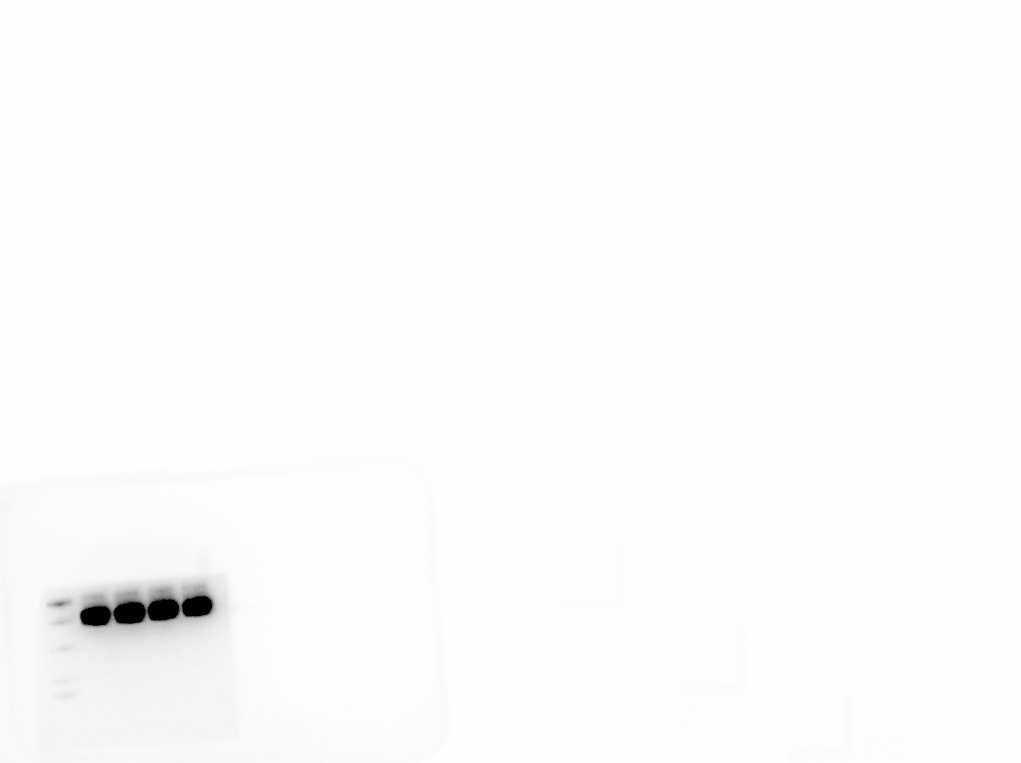


total Tau

50kDa

38kDa

28kDa

22kDa


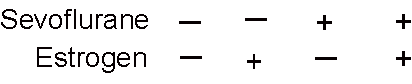

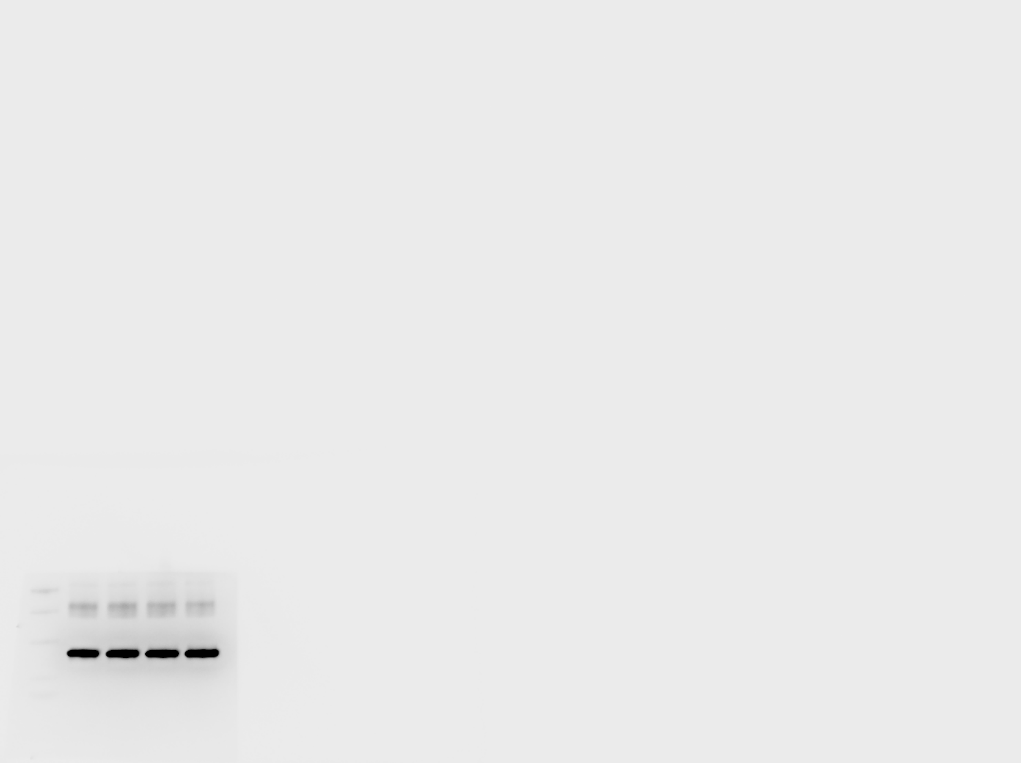


50kDa

38kDa

28kDa

22kDa

GAPDH

Full unedited blot for Figure 2B total Tau and GAPDH


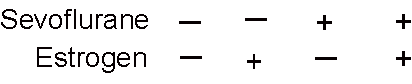

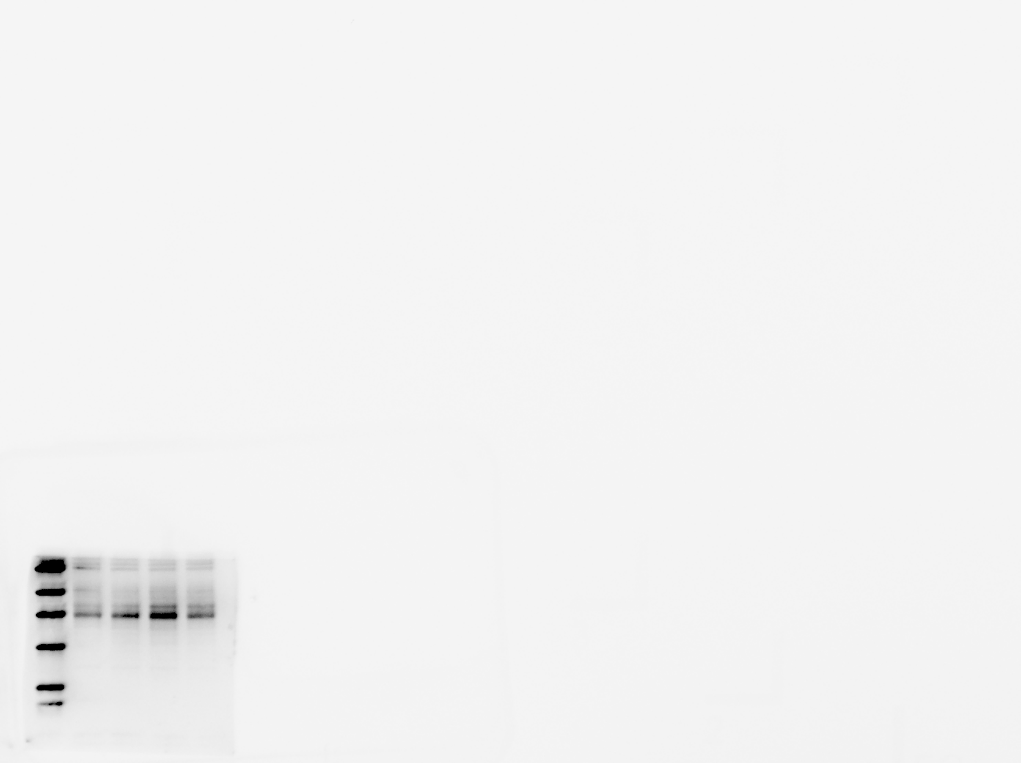


P-Tau-Ser202/Thr205

P-Tau-Ser202/Thr205

50kDa

38kDa

28kDa

22kDa


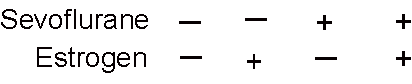

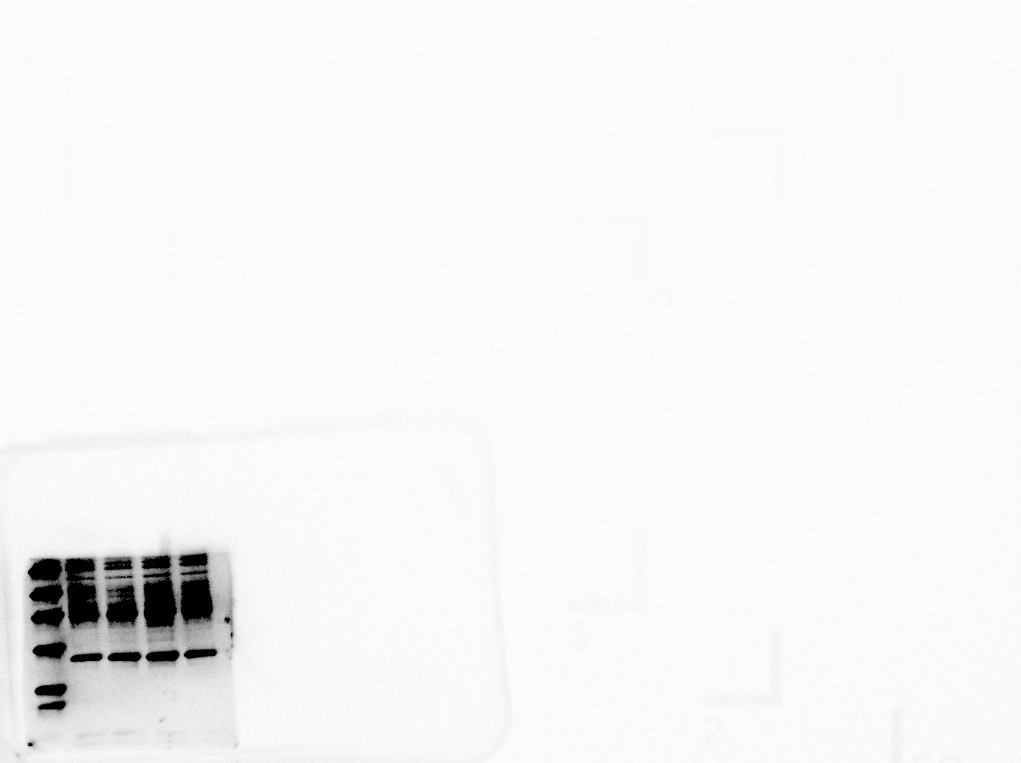


50kDa

38kDa

28kDa

22kDa

GAPDH

Full unedited blot for Figure 2B P-Tau-Ser202/Thr205 and GAPDH


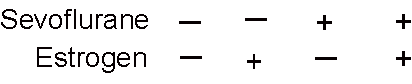

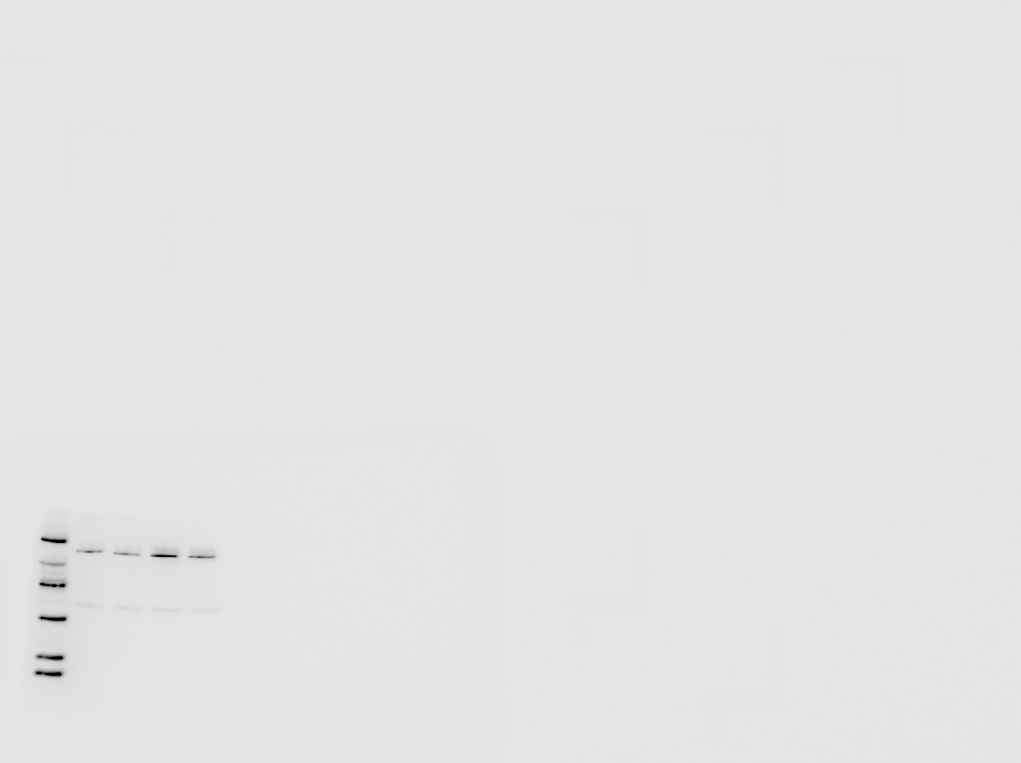


P-Tau-Ser396/404

62kDa

50kDa

38kDa

28kDa

22kDa

62kDa

50kDa

38kDa

28kDa

22kDa


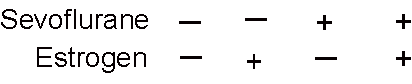

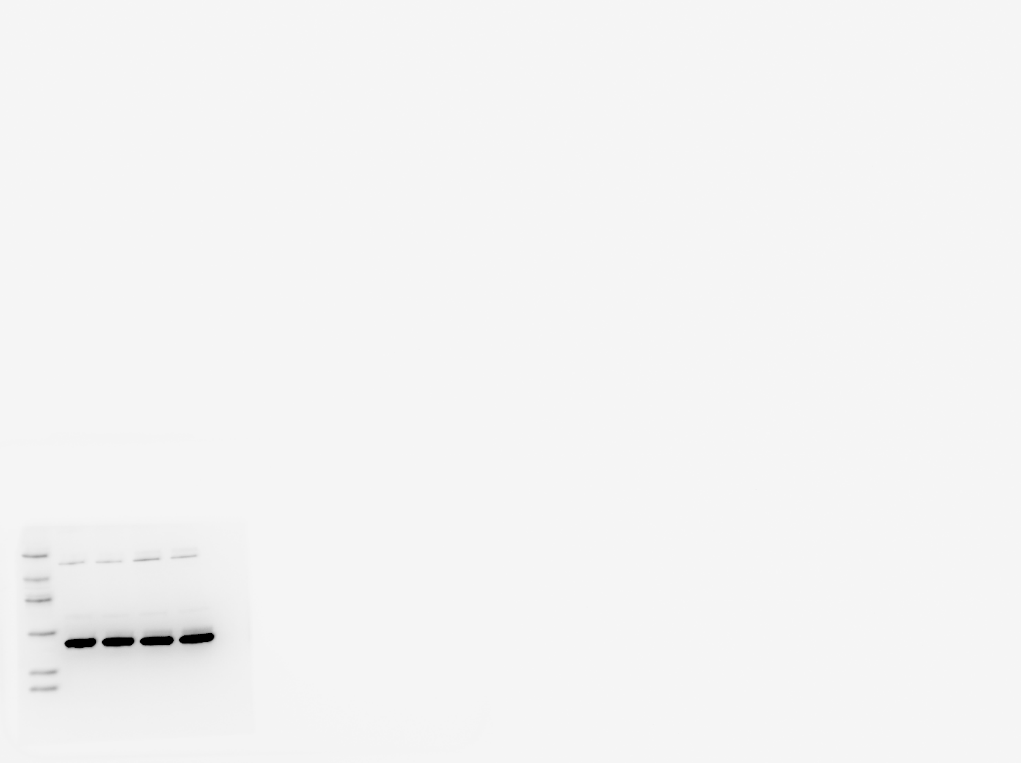


62kDa

50kDa

38kDa

28kDa

22kDa

GAPDH

Full unedited blot for Figure 2B P-Tau-Ser396/404 and GAPDH

Figure 2E


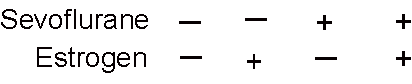

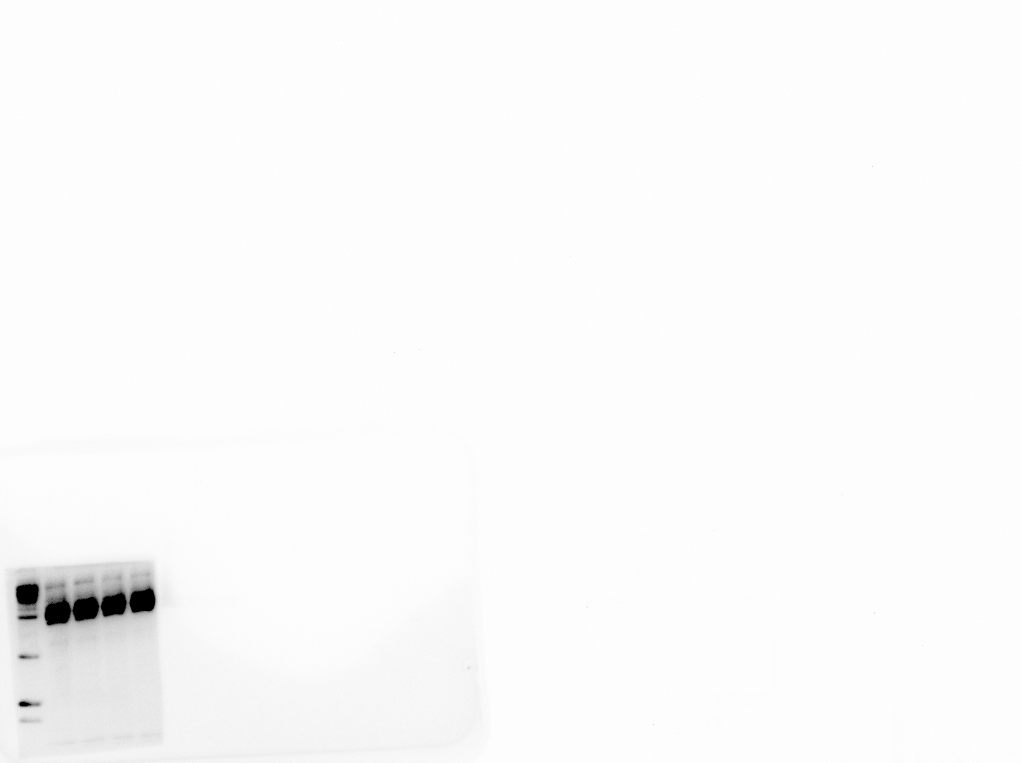


total Tau

50kDa

38kDa

28kDa

22kDa


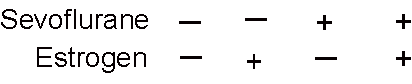

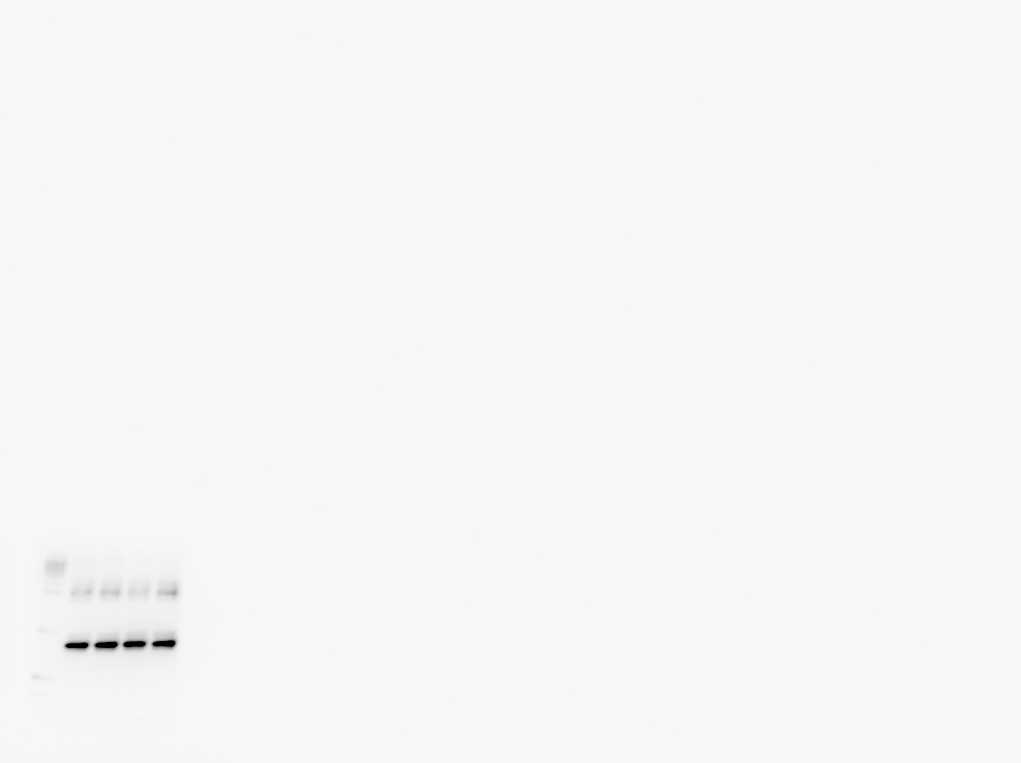


GAPDH

50kDa

38kDa

28kDa

22kDa

Full unedited blot for Figure 2E total Tau and GAPDH


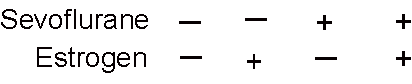

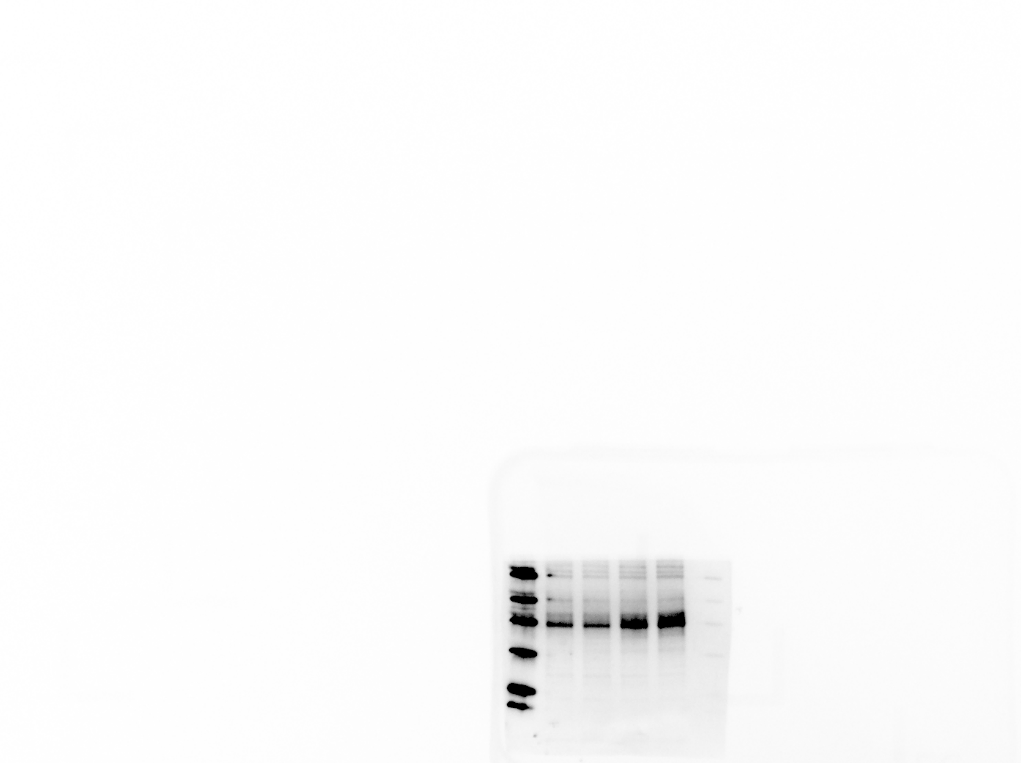


P-Tau-Ser202/Thr205

50kDa

38kDa

28kDa

22kDa


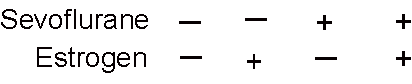

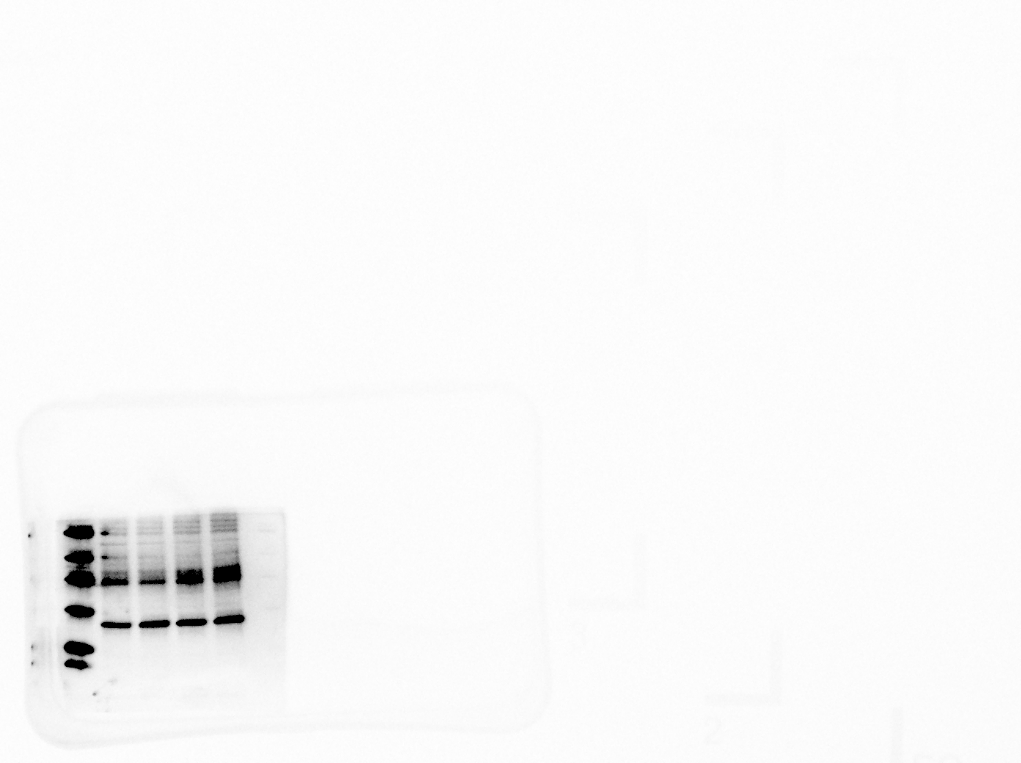


50kDa

38kDa

28kDa

22kDa

GAPDH

Full unedited blot for Figure 2E P-Tau-Ser202/Thr205 and GAPDH


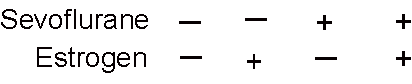

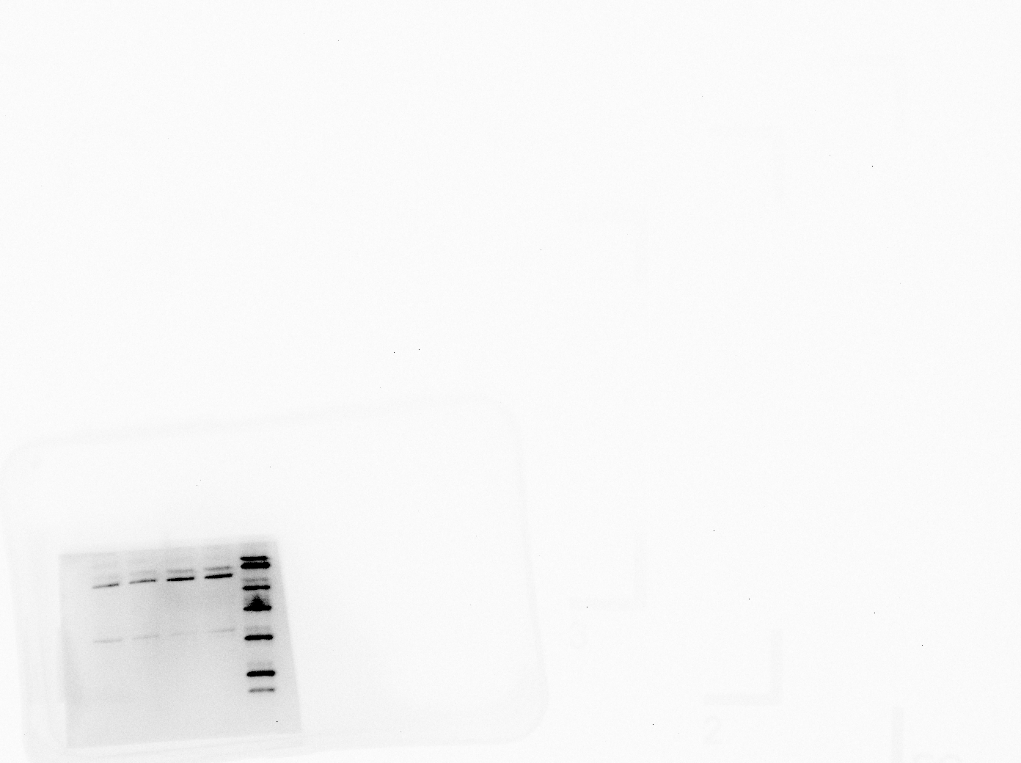


P-Tau-Ser396/404

62kDa

50kDa

38kDa

28kDa

22kDa


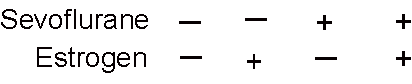

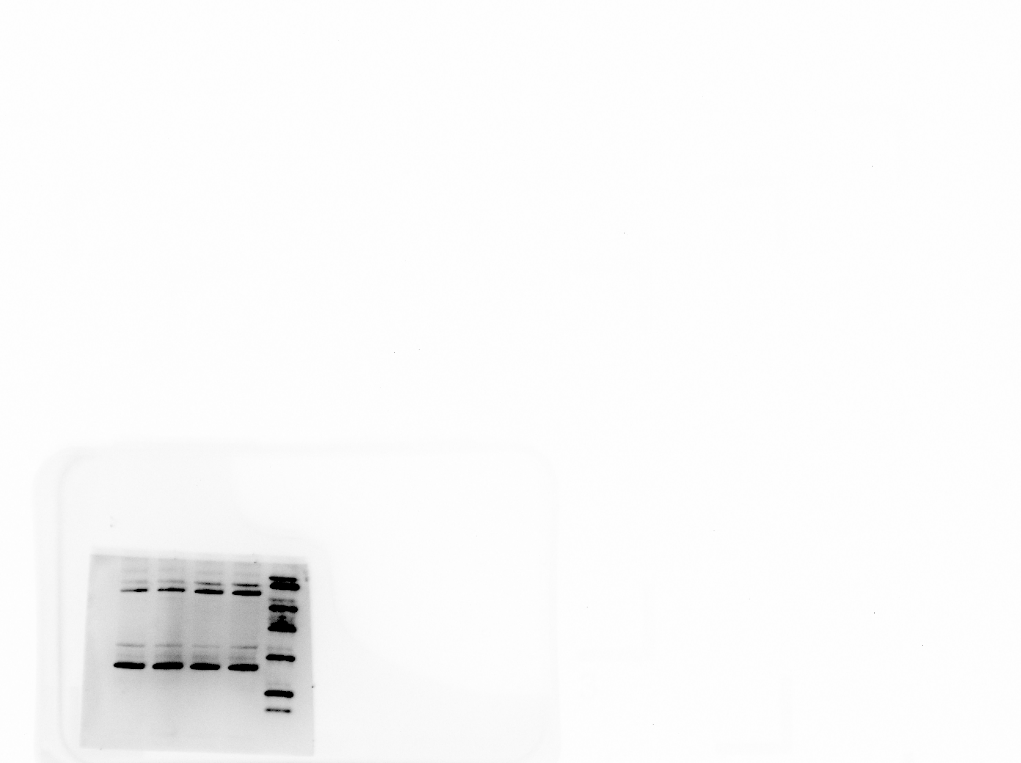


62kDa

50kDa

38kDa

28kDa

22kDa

GAPDH

Full unedited blot for Figure 2B P-Tau-Ser396/404 and GAPDH

Figure 4B


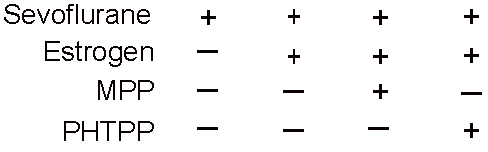

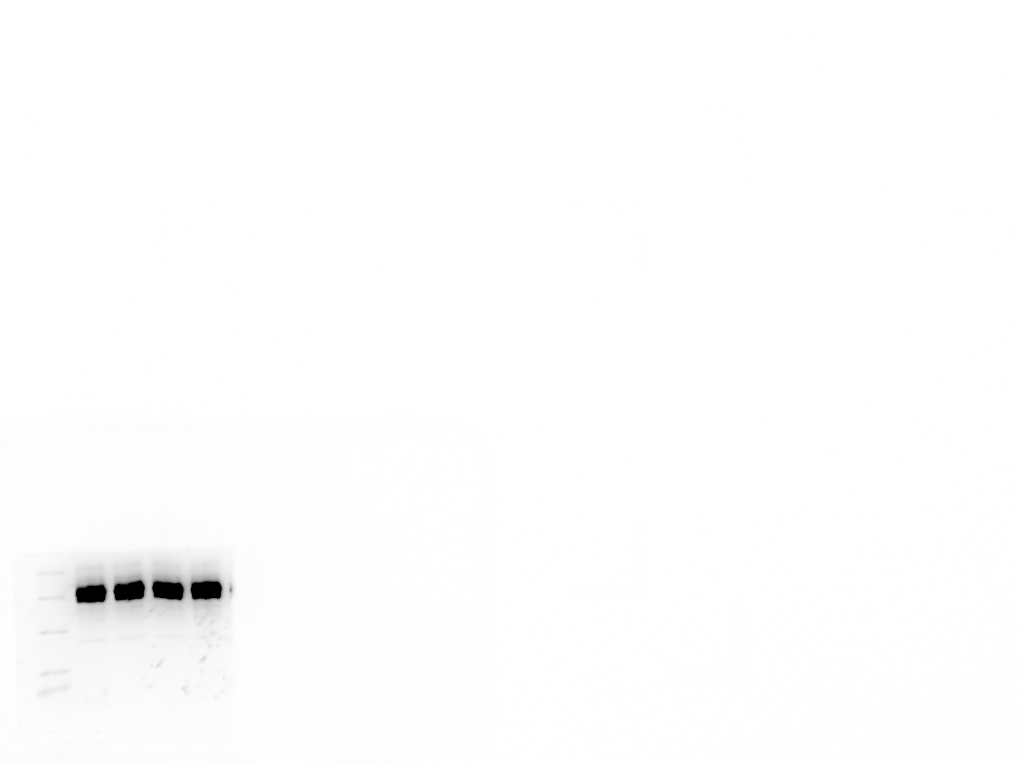


total Tau

50kDa

38kDa

28kDa

22kDa


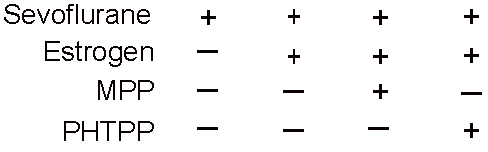

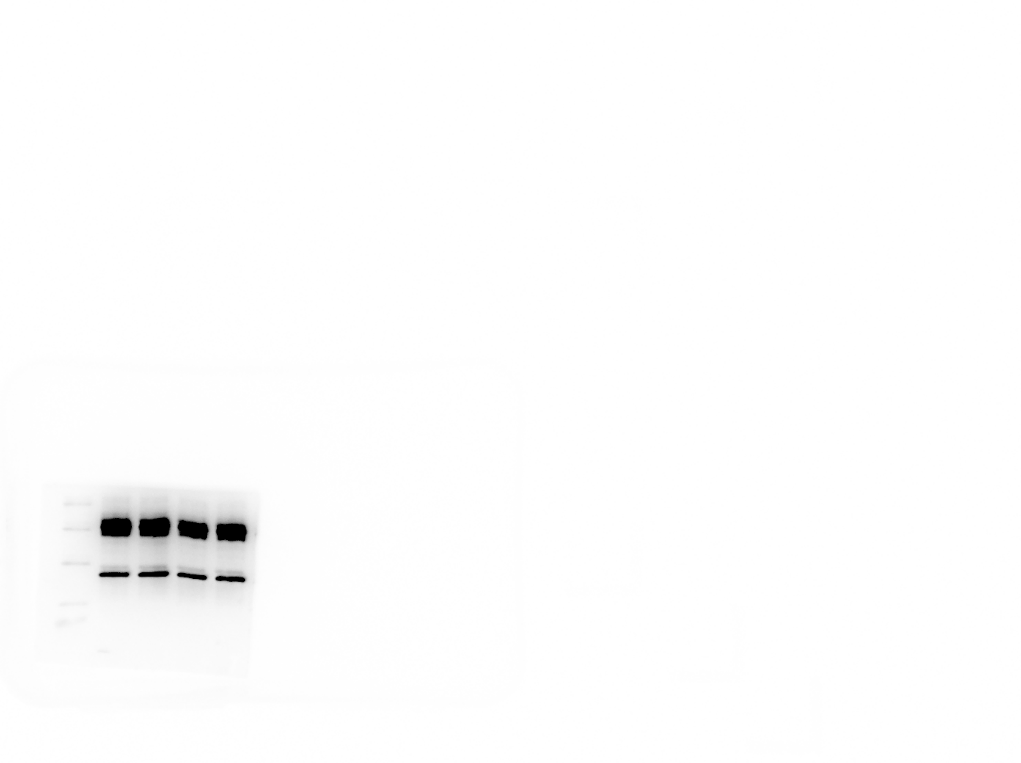


GAPDH

50kDa

38kDa

28kDa

22kDa

Full unedited blot for Figure 4B total Tau and GAPDH


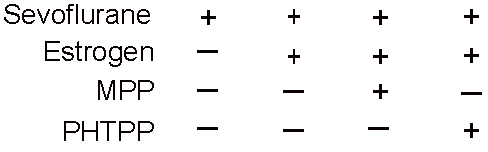

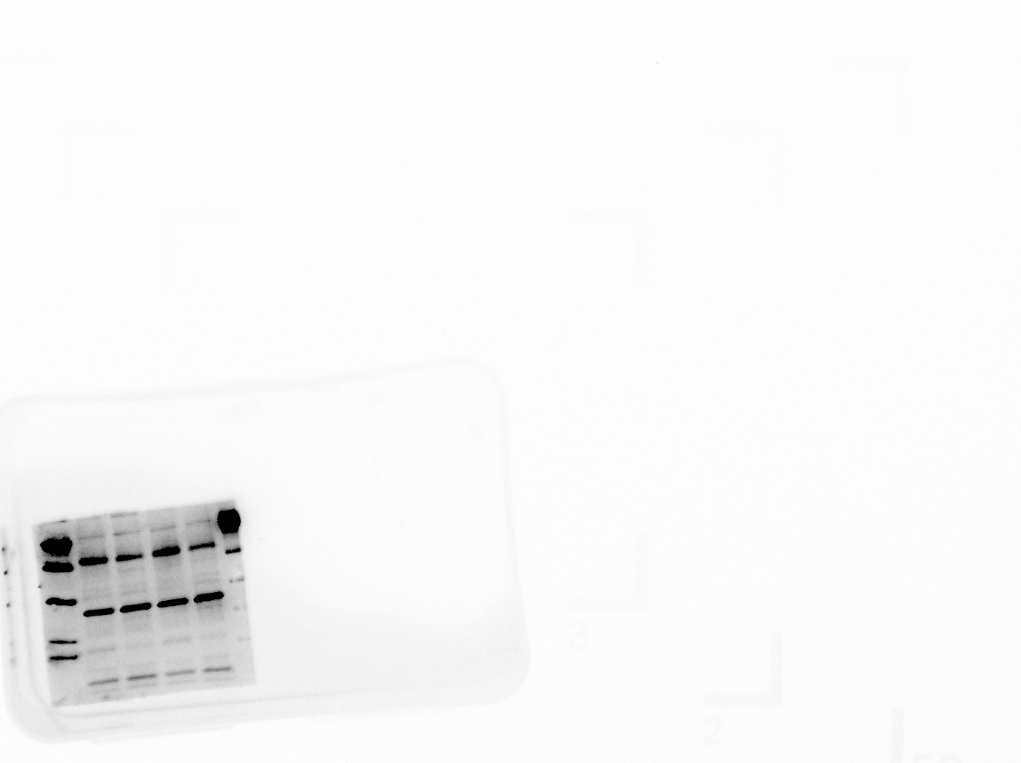


P-Tau-Ser202/Thr205

50kDa

38kDa

28kDa

22kDa


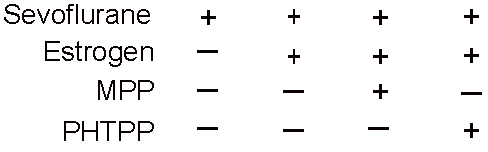

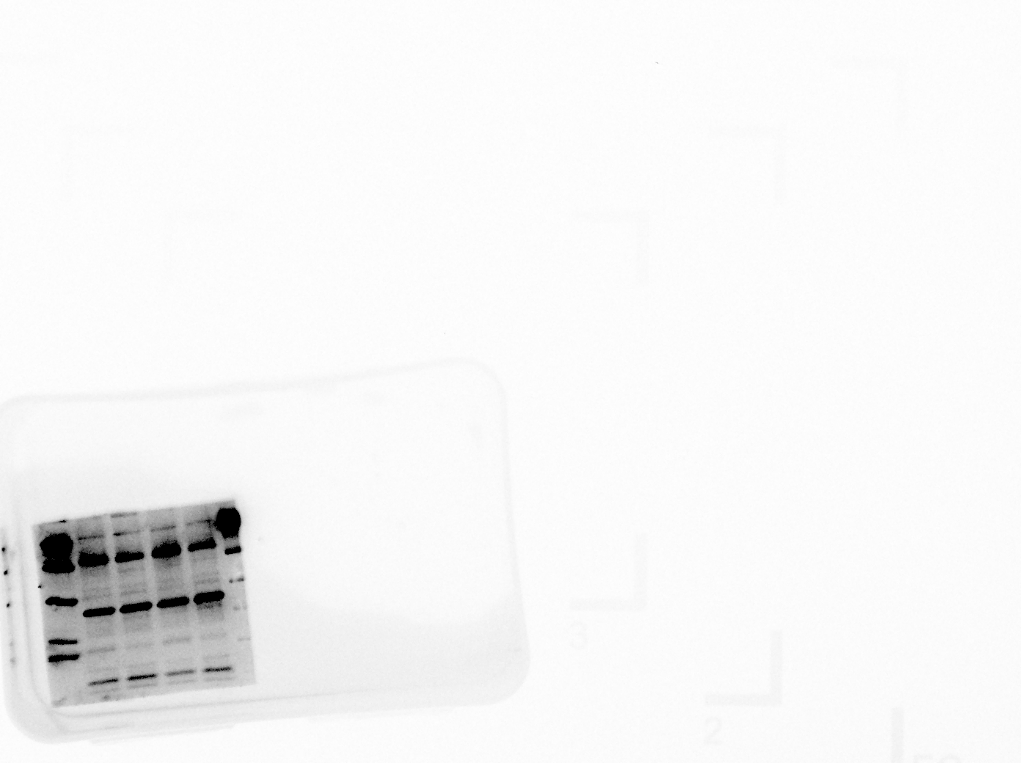


GAPDH

50kDa

38kDa

28kDa

22kDa

Full unedited blot for Figure 4B P-Tau-Ser202/Thr205 and GAPDH


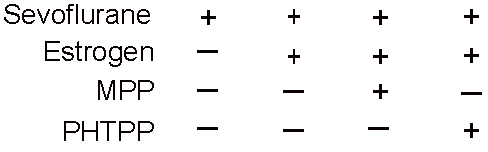

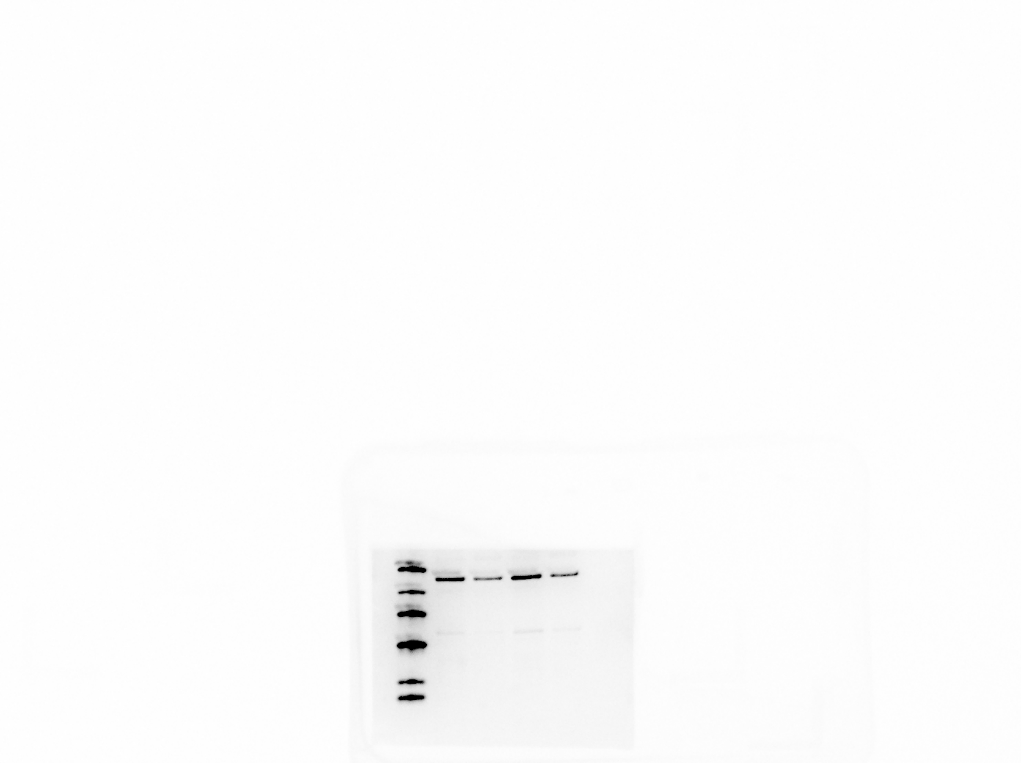


P-Tau-Ser396/404

62kDa

50kDa

38kDa

28kDa

22kDa


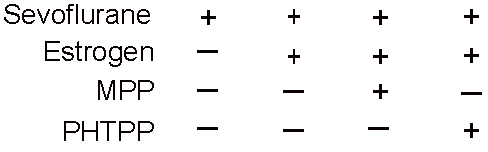

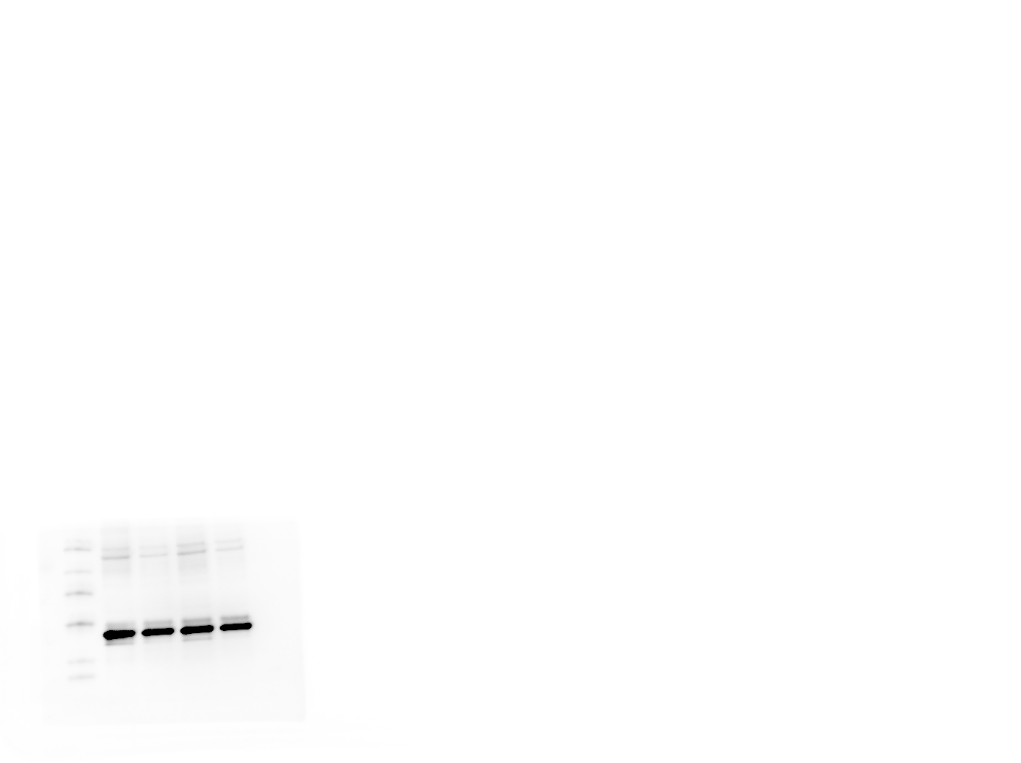


GAPDH

62kDa

50kDa

38kDa

28kDa

22kDa

Full unedited blot for Figure 4B P-Tau-Ser396/404 and GAPDH

Figure 5C


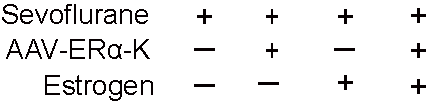

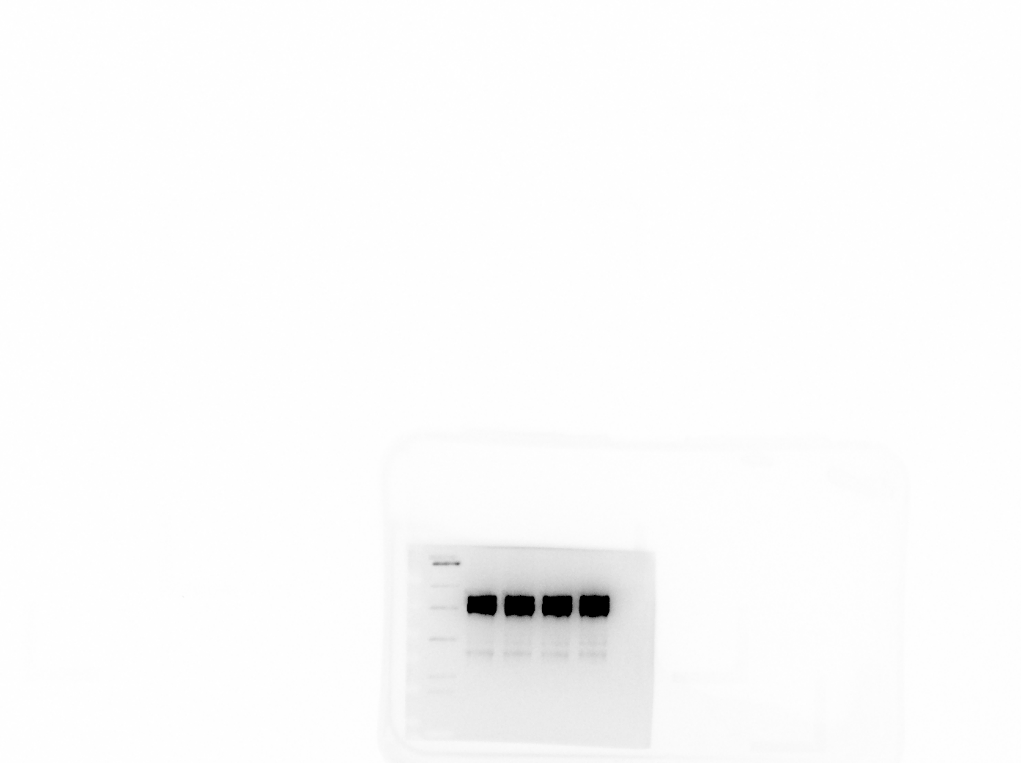


total Tau

50kDa

38kDa

28kDa

22kDa


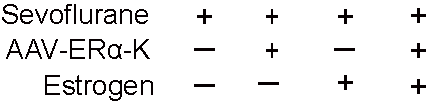

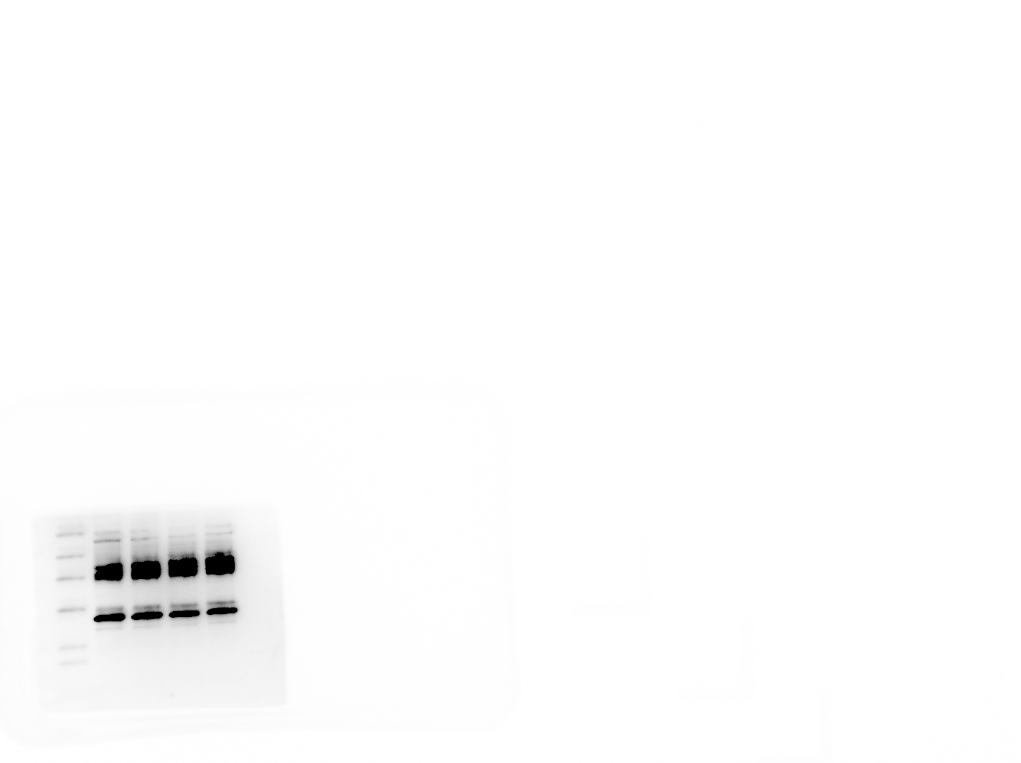


GAPDH

50kDa

38kDa

28kDa

22kDa

Full unedited blot for Figure 5C total Tau and GAPDH


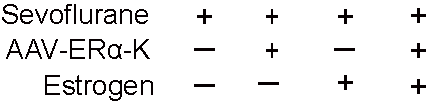

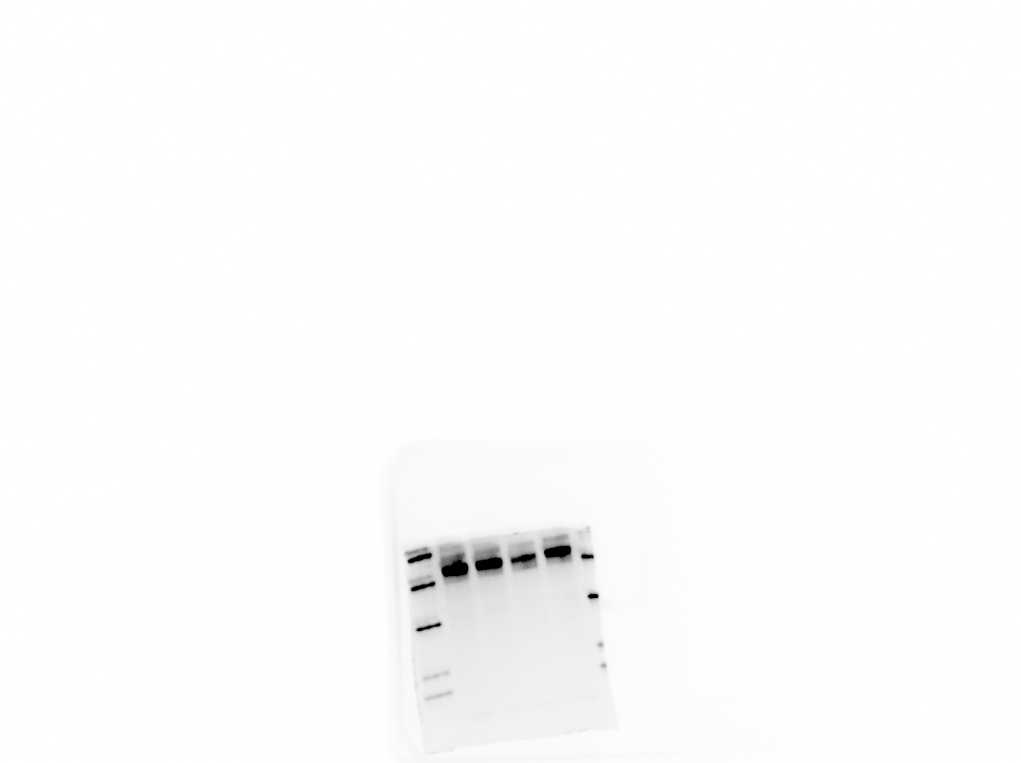


P-Tau-Ser202/Thr205

50kDa

38kDa

28kDa

22kDa


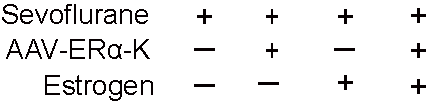

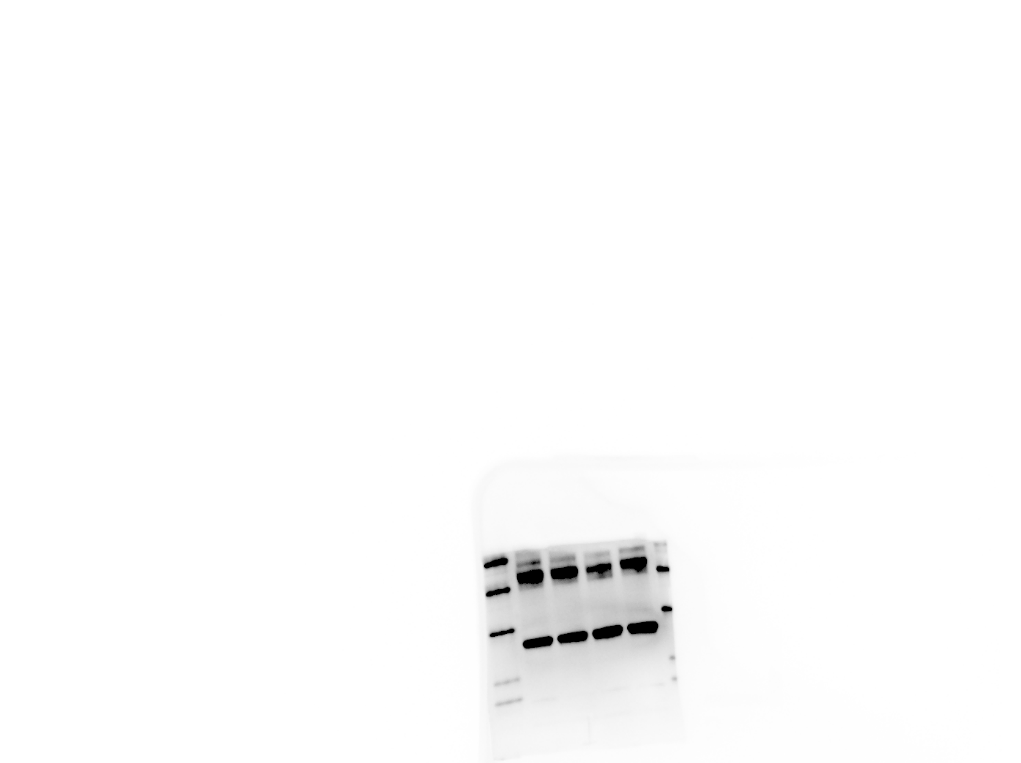


GAPDH

50kDa

38kDa

28kDa

22kDa

Full unedited blot for Figure 5C P-Tau-Ser202/Thr205 and GAPDH


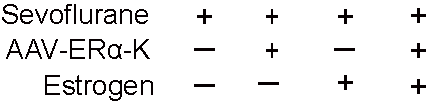

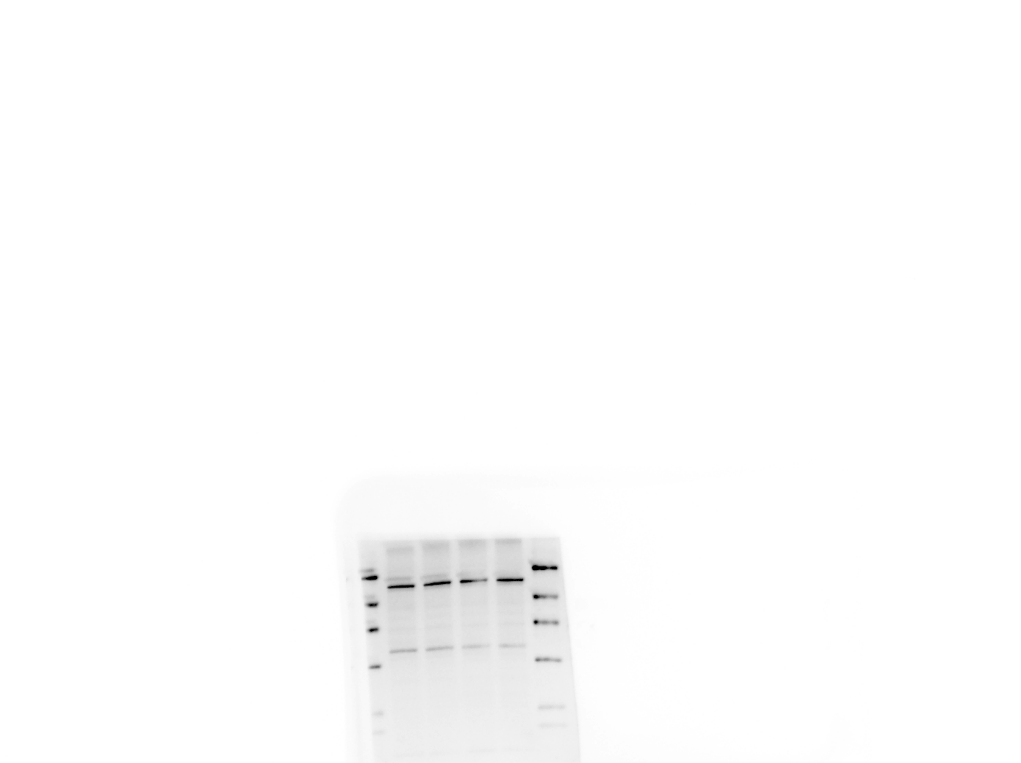


P-Tau-Ser396/404

62kDa

50kDa

38kDa

28kDa

22kDa


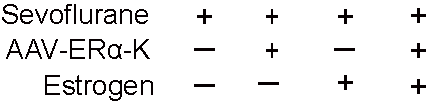

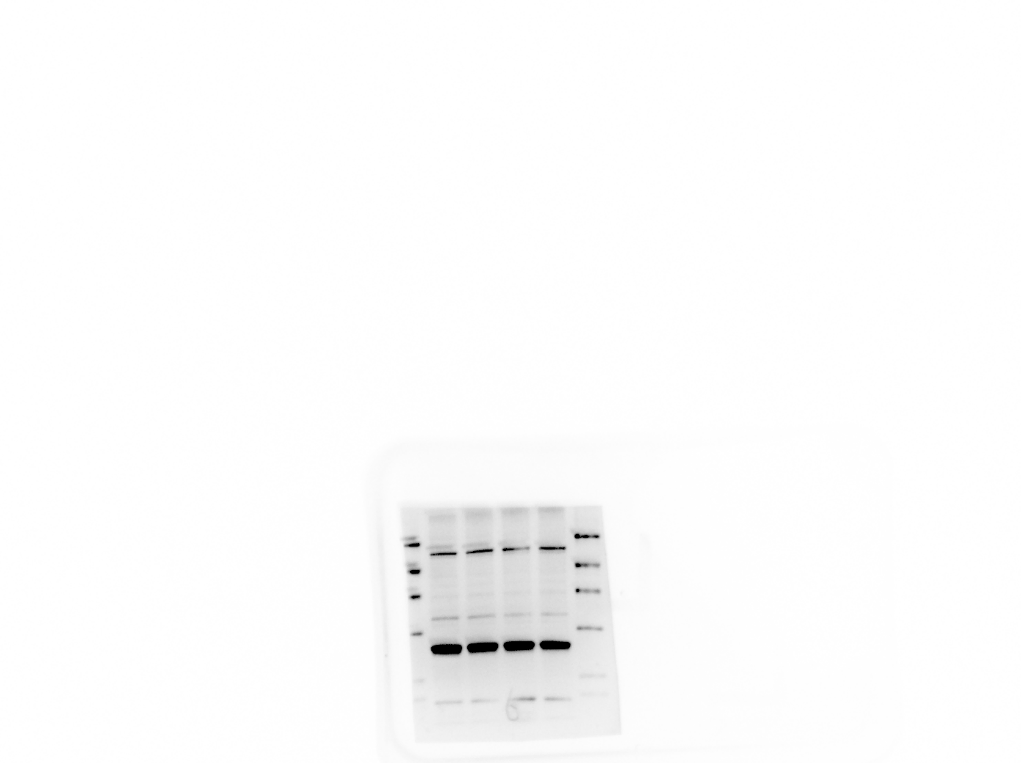


GAPDH

62kDa

50kDa

38kDa

28kDa

22kDa

Full unedited blot for Figure 5C P-Tau-Ser396/404 and GAPDH

Figure 5E


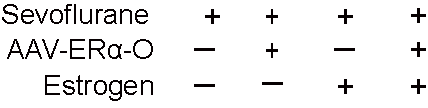

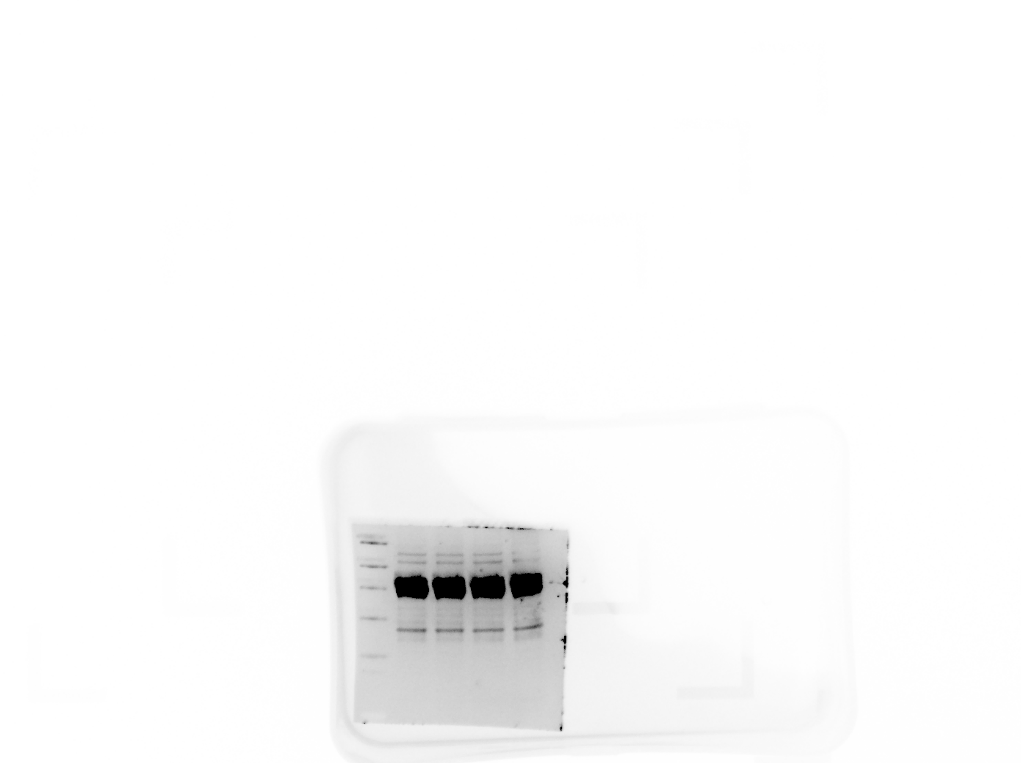


total Tau

50kDa

38kDa

28kDa

22kDa


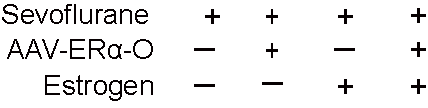

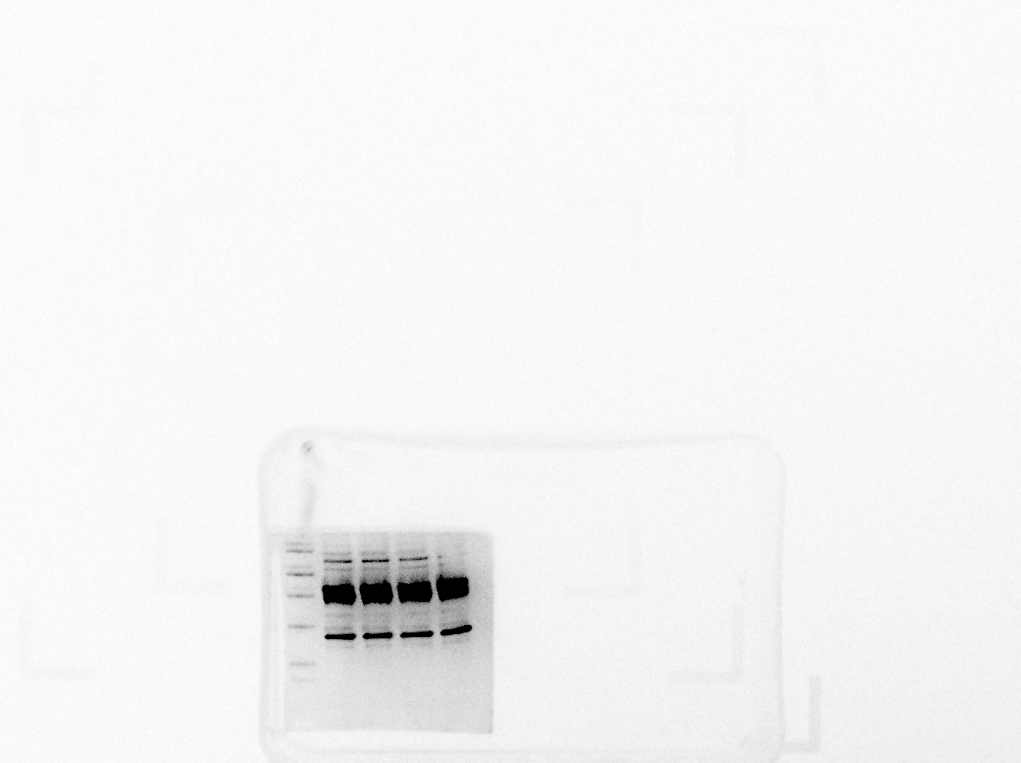


GAPDH

50kDa

38kDa

28kDa

22kDa

Full unedited blot for Figure 5E total Tau and GAPDH


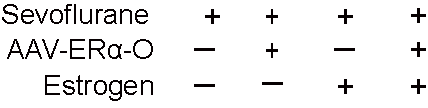

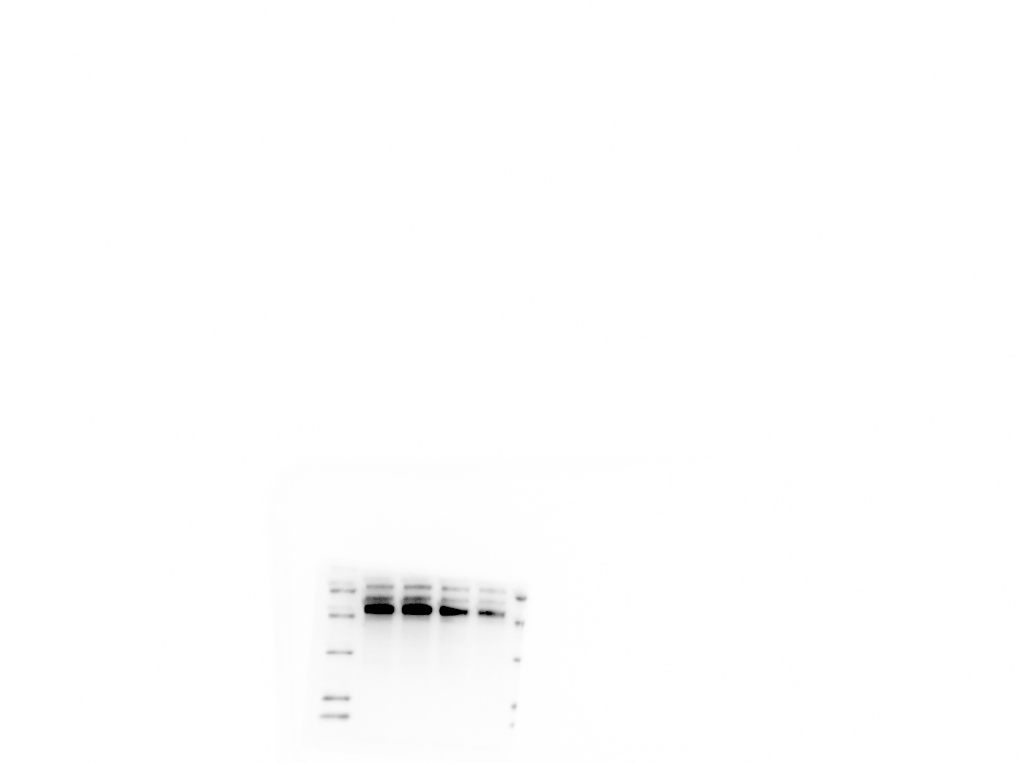


P-Tau-Ser202/Thr205

50kDa

38kDa

28kDa

22kDa


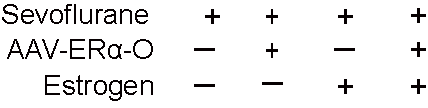

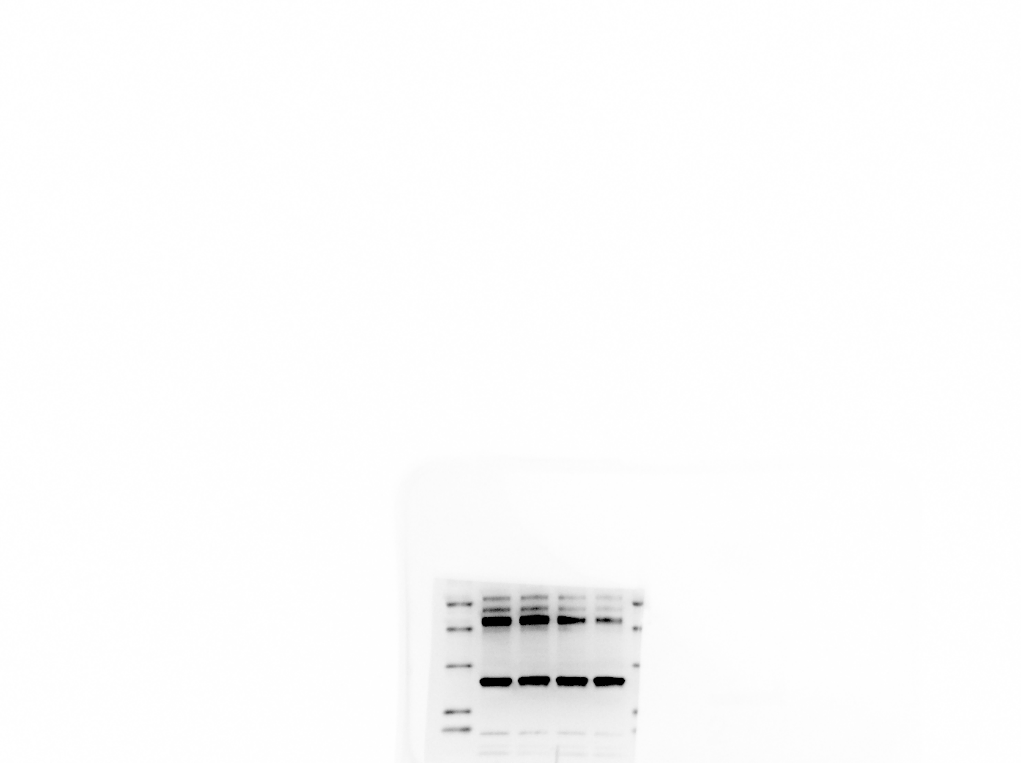


GAPDH

50kDa

38kDa

28kDa

22kDa

Full unedited blot for Figure 5E P-Tau-Ser202/Thr205 and GAPDH


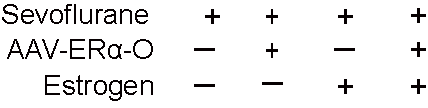

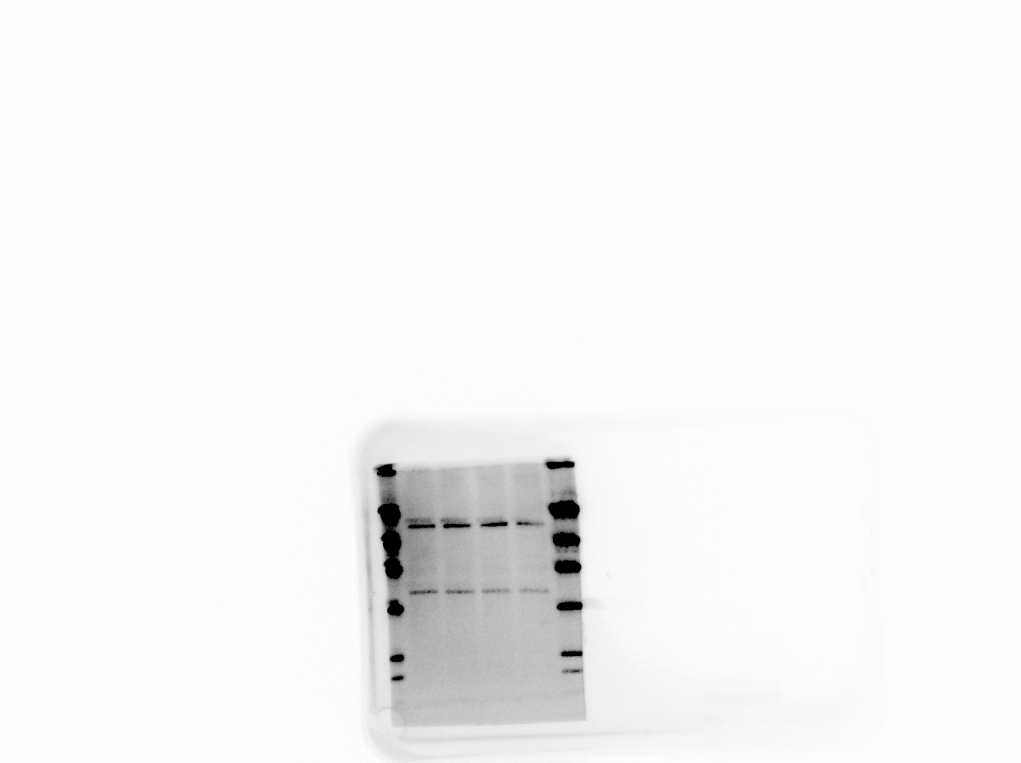


P-Tau-Ser396/404

50kDa

38kDa

28kDa

22kDa


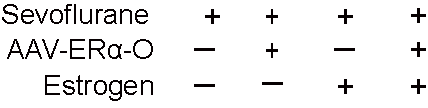

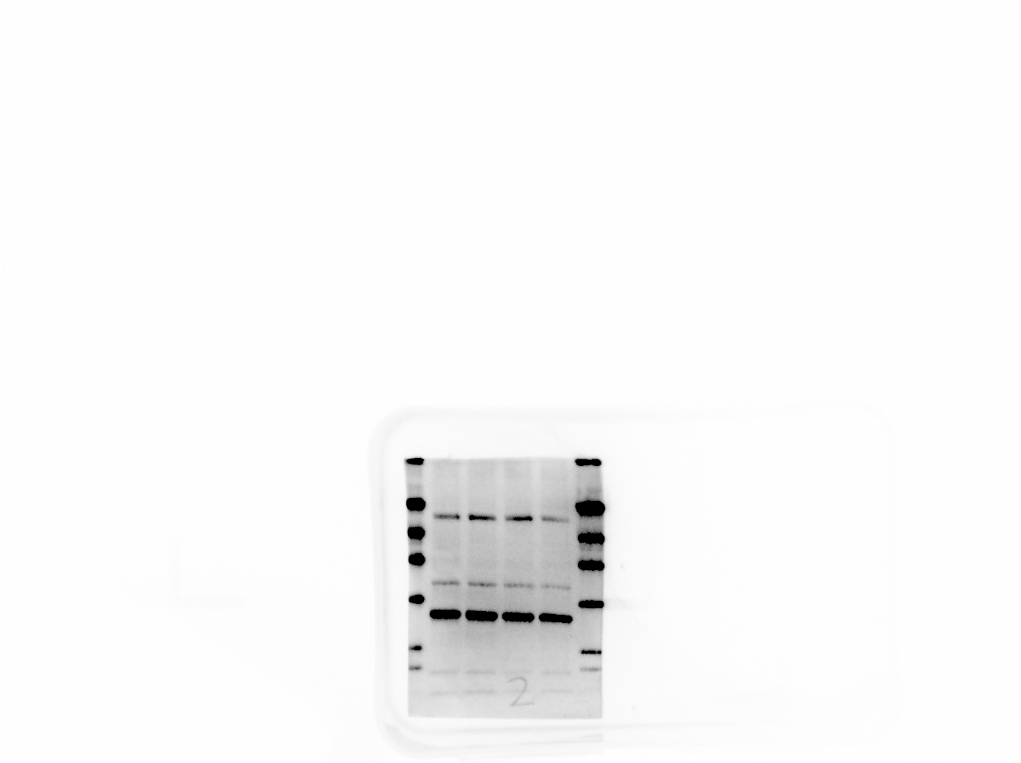


50kDa

38kDa

28kDa

22kDa

GAPDH

Full unedited blot for Figure 5E P-Tau-Ser396/404 and GAPDH

Figure 7D


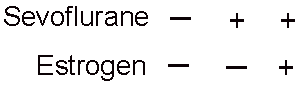

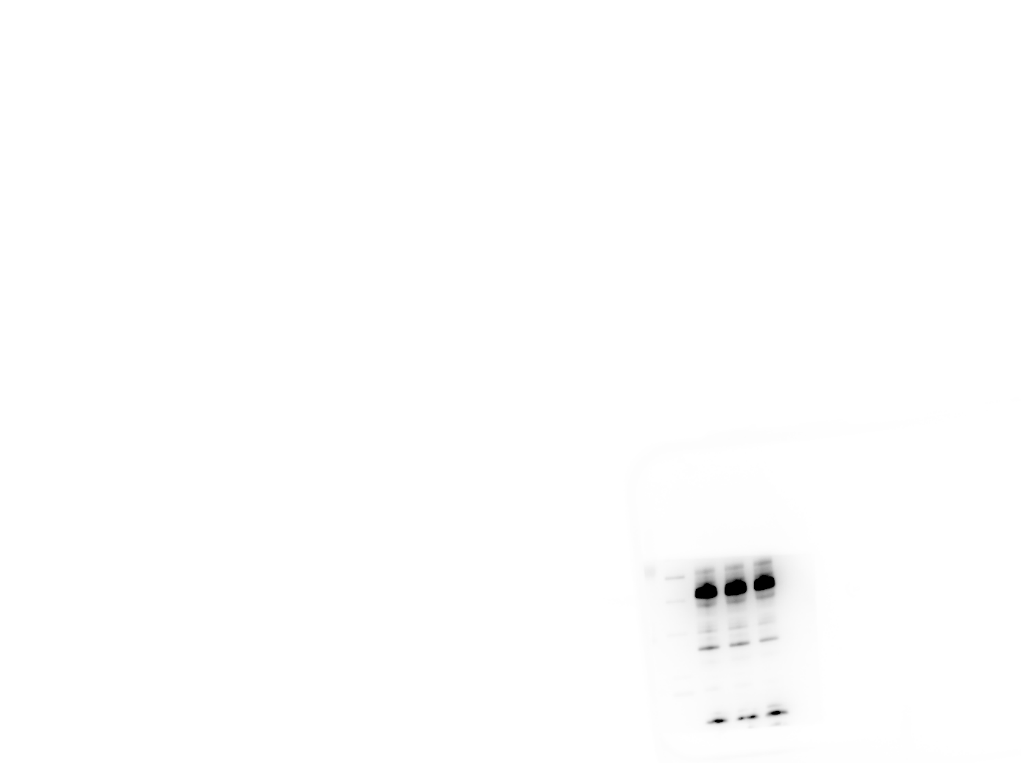


50kDa

38kDa

28kDa

22kDa

Tau

Full unedited blot for Figure 7D Input-Tau


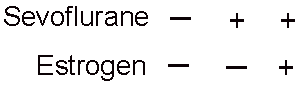

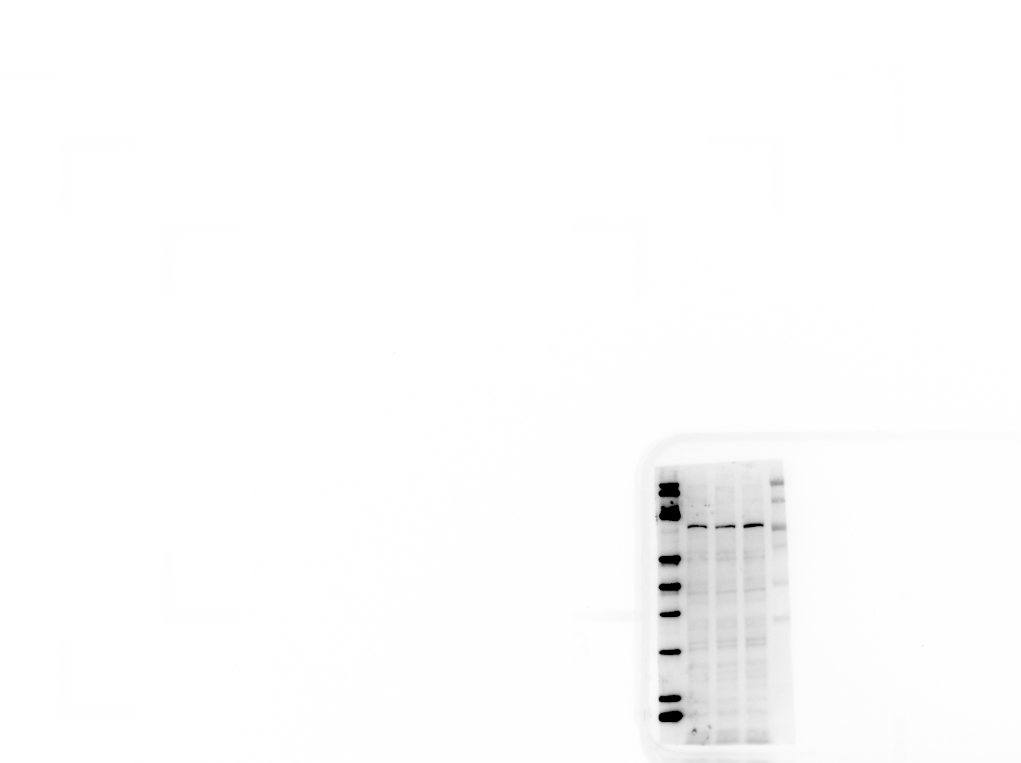


62kDa

50kDa

38kDa

28kDa

22kDa

ERα

Full unedited blot for Figure 7D Input-ERα


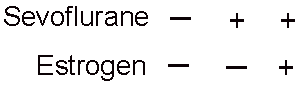

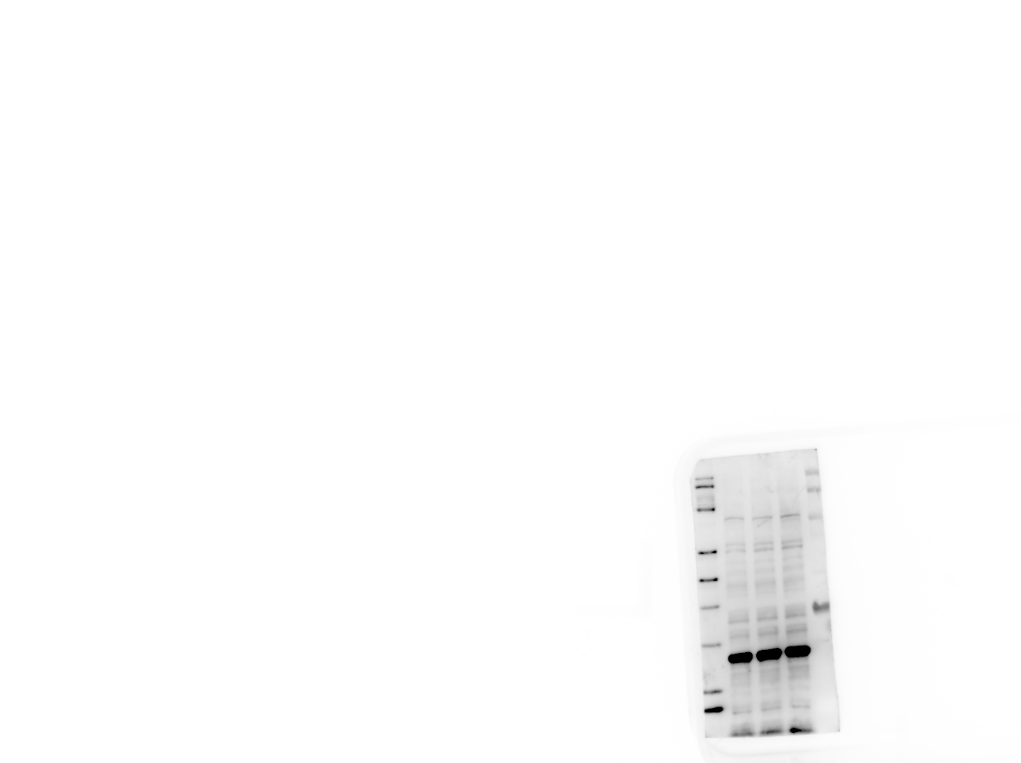


62kDa

50kDa

38kDa

28kDa

22kDa

62kDa

50kDa

38kDa

28kDa

22kDa

GAPDH

Full unedited blot for Figure 7D Input-GAPDH


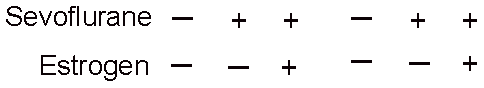

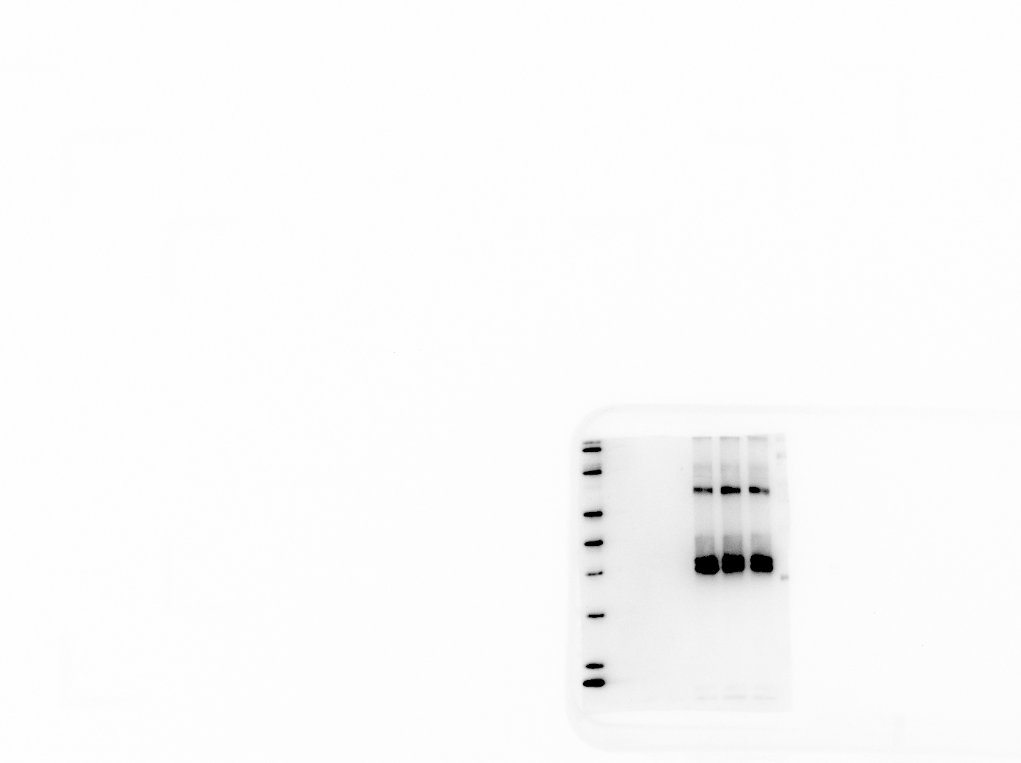


IgG

ERα

Tau

62kDa

50kDa

38kDa

28kDa

22kDa

Full unedited blot for Figure 7D IP

Supplementary Figure 3G


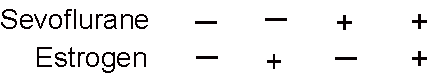

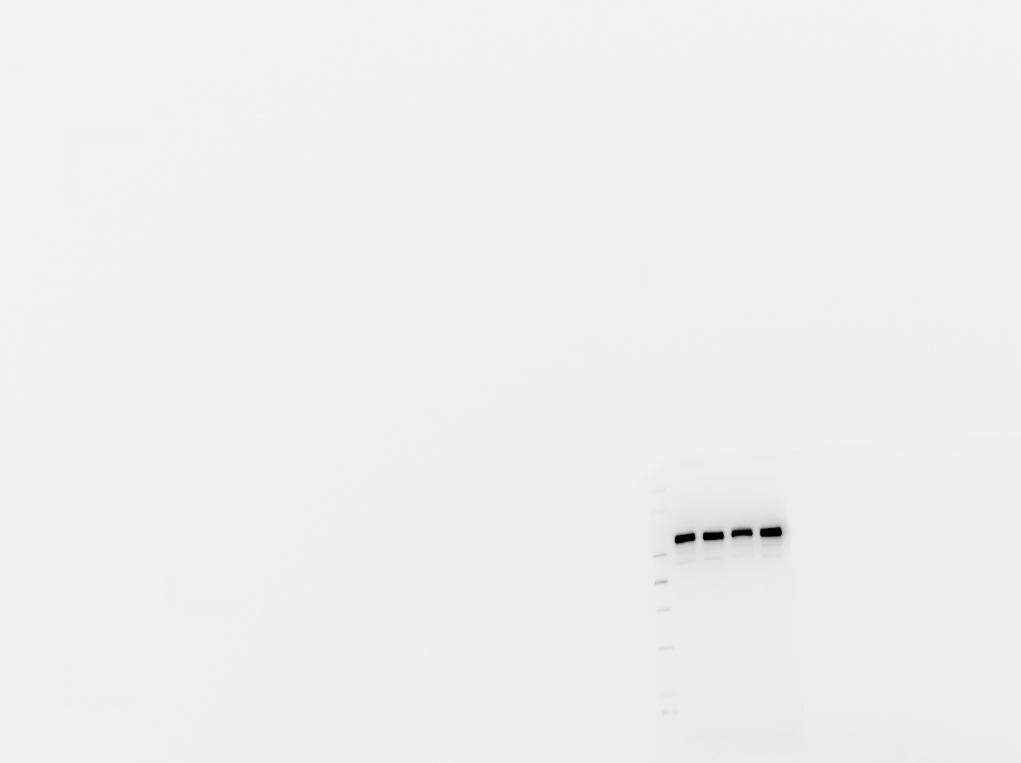


PSD95

113kDa78kDa

62kDa

50kDa

38kDa

28kDa

22kDa


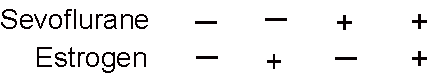

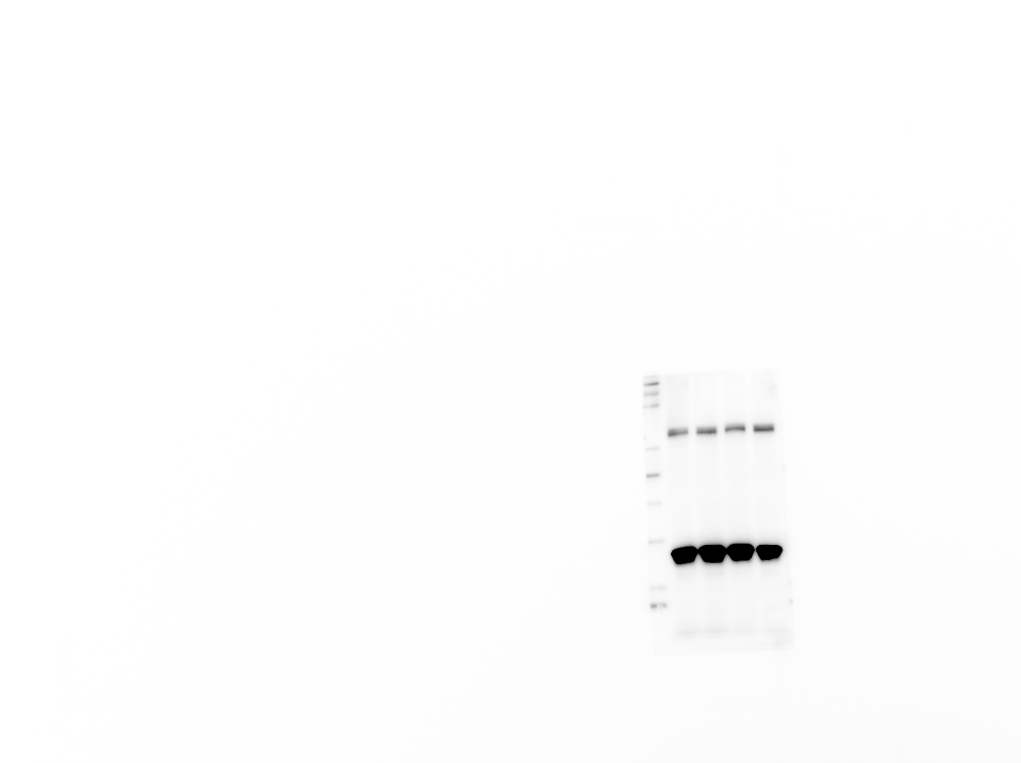


GAPDH

113kDa78kDa

62kDa

50kDa

38kDa

28kDa

22kDa

Full unedited blot for Supplementary Figure 3G PSD95 and GAPDH

Supplementary Figure 3I


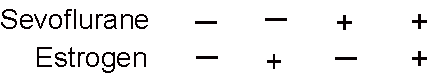

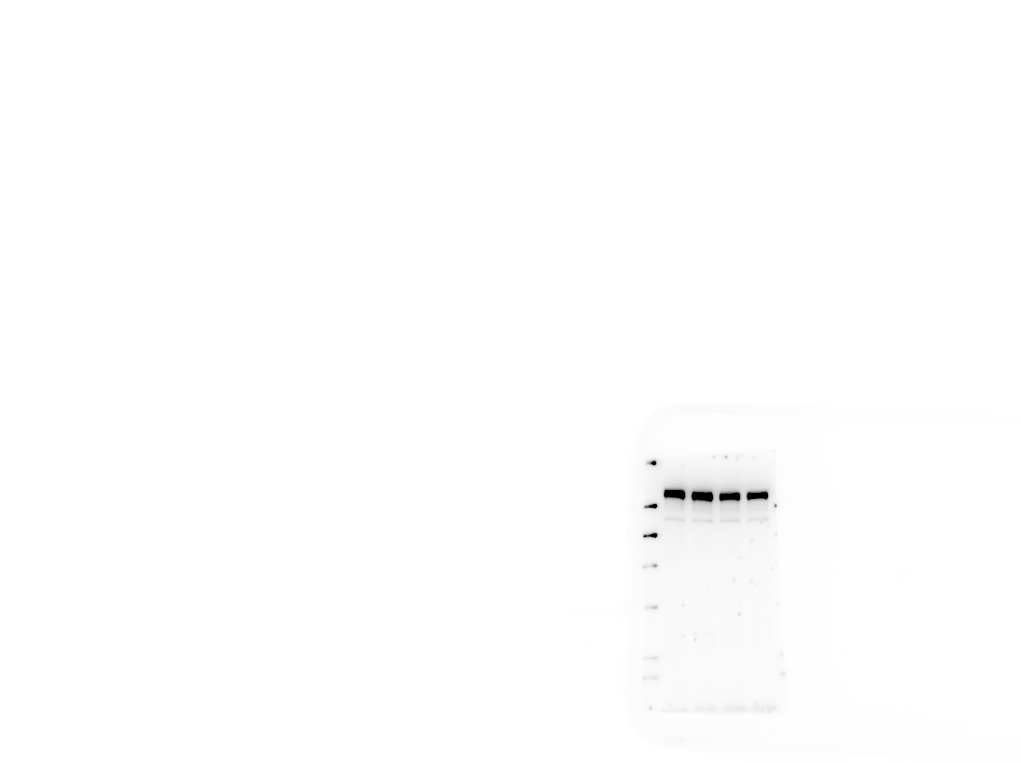


PSD95

113kDa78kDa

62kDa

50kDa

38kDa

28kDa

22kDa


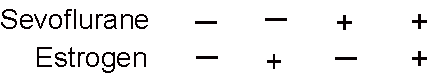

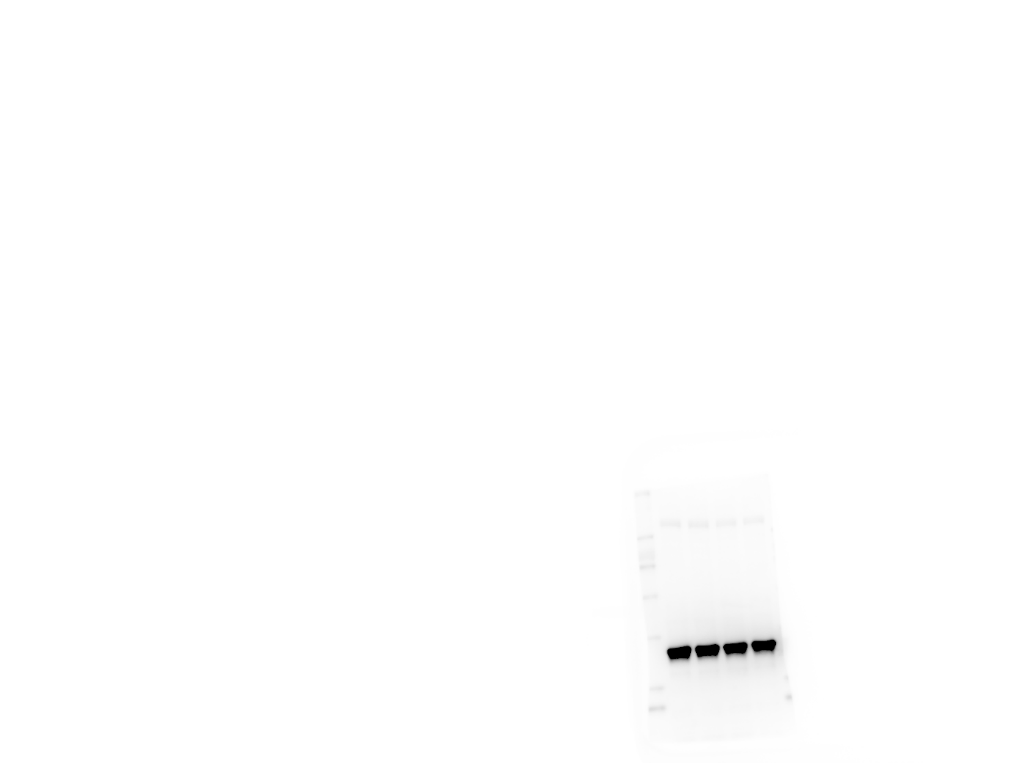


GAPDH

113kDa78kDa

62kDa

50kDa

38kDa

28kDa

22kDa

Full unedited blot for Supplementary Figure 3I PSD95 and GAPDH

Supplementary Figure 5C


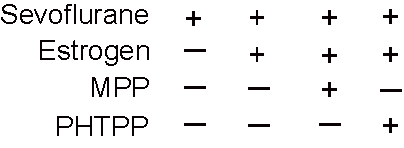

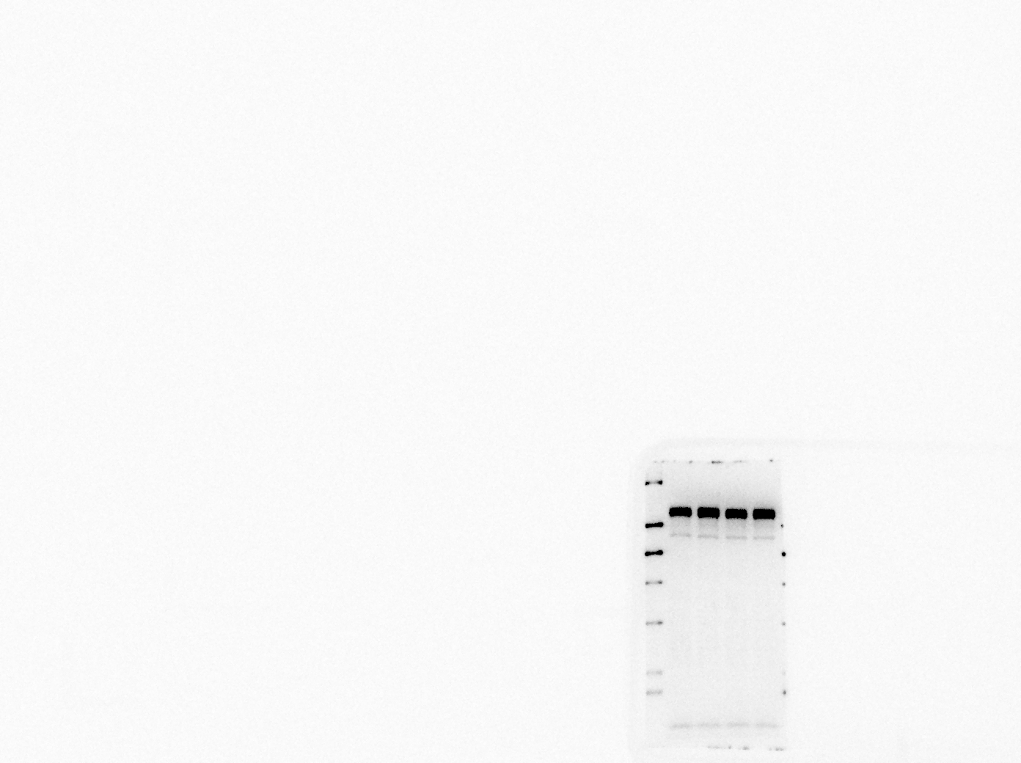


PSD95

113kDa78kDa

62kDa

50kDa

38kDa

28kDa

22kDa


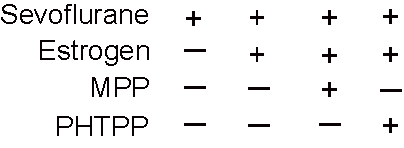

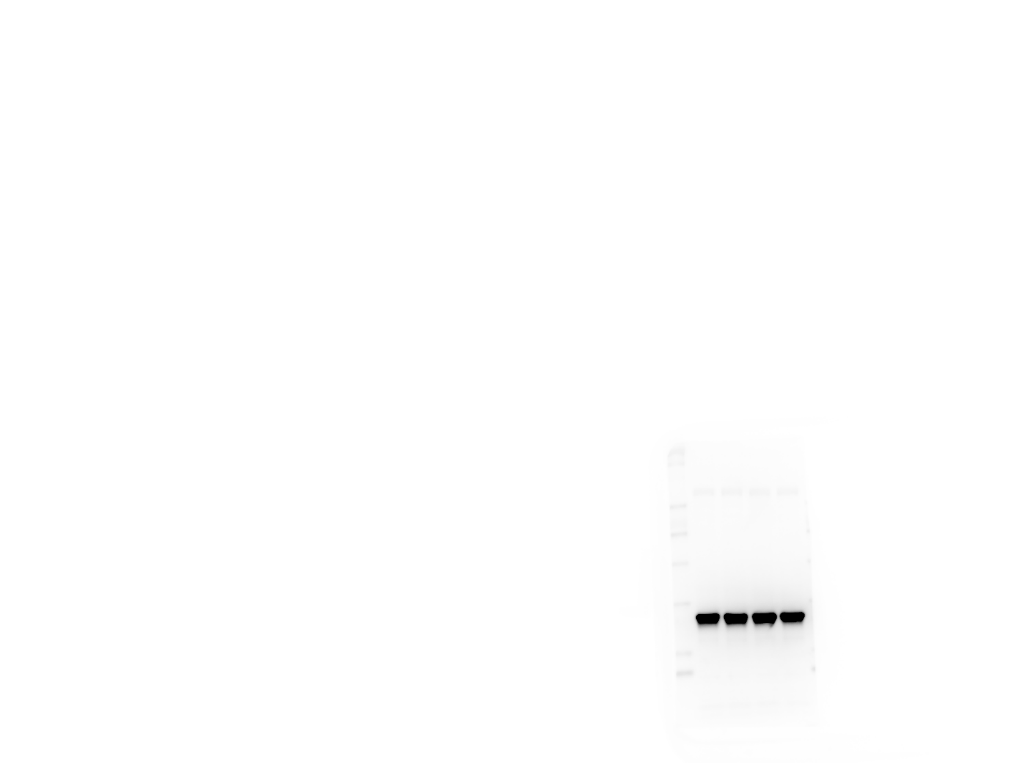


GAPDH

113kDa78kDa

62kDa

50kDa

38kDa

28kDa

22kDa

Full unedited blot for Supplementary Figure 5C PSD95 and GAPDH

Supplementary Figure 6A


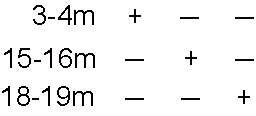

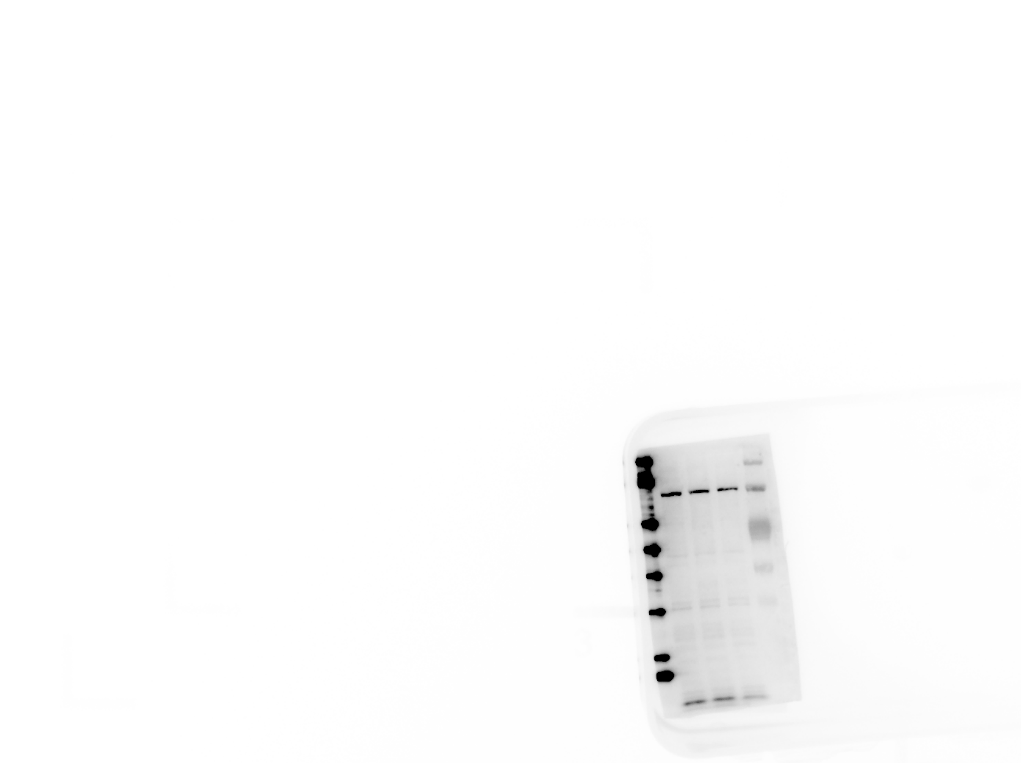


ERα

78kDa

62kDa

50kDa

38kDa

28kDa

22kDa


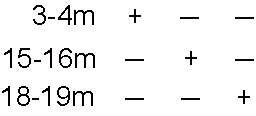

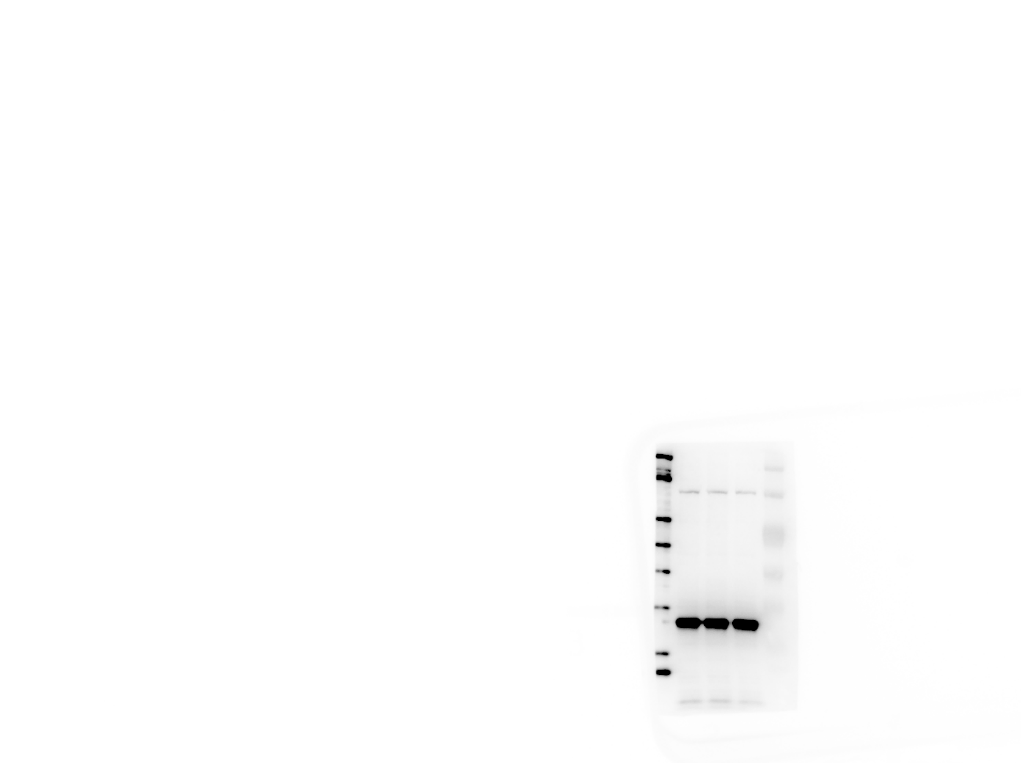


GAPDH

78kDa

62kDa

50kDa

38kDa

28kDa

22kDa

Full unedited blot for Supplementary Figure 6A ERα and GAPDH

Supplementary Figure 6C


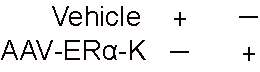

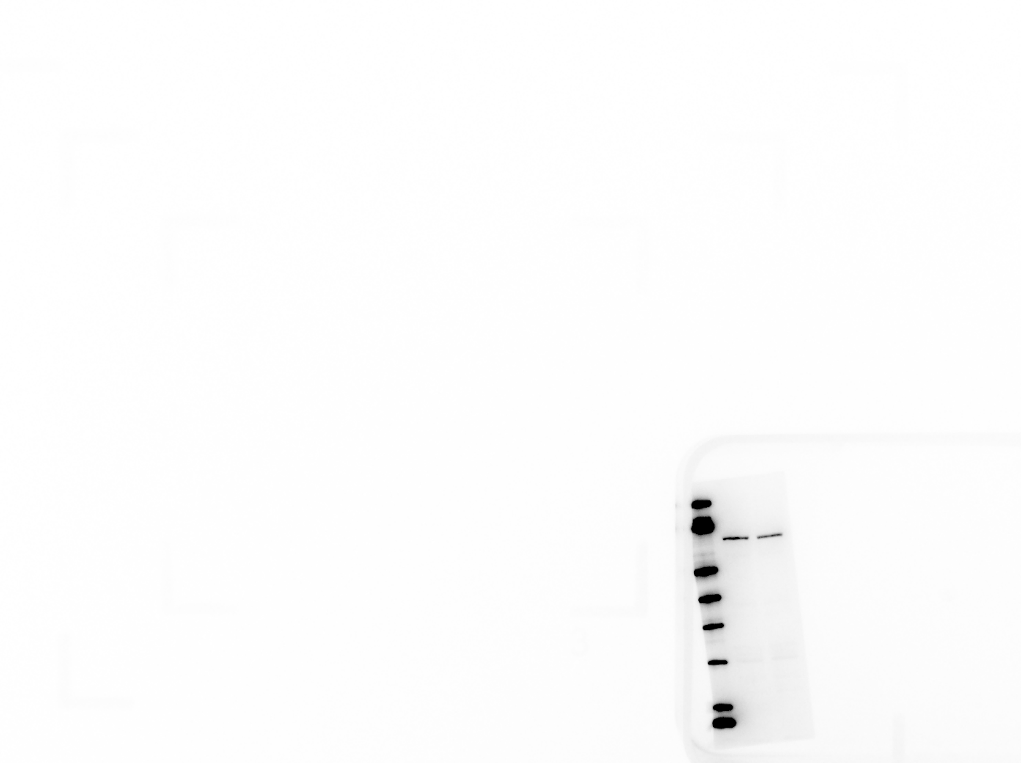


ERα

78kDa

62kDa

50kDa

38kDa

28kDa

22kDa


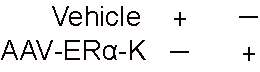

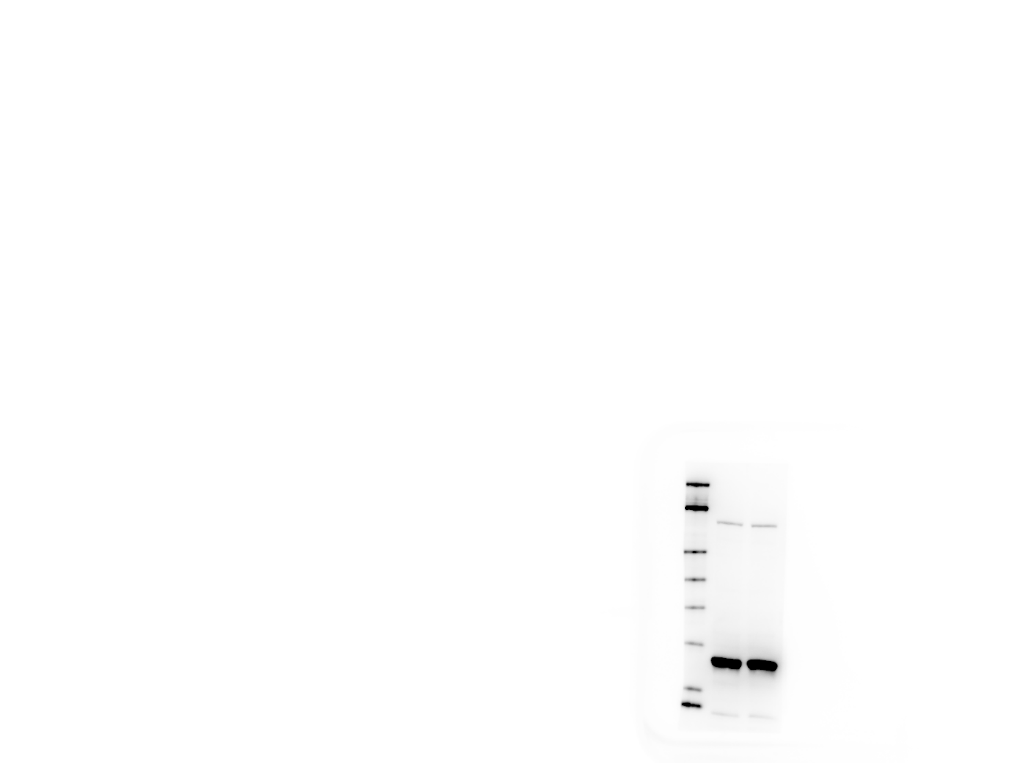


GAPDH

78kDa

62kDa

50kDa

38kDa

28kDa

22kDa

Full unedited blot for Supplementary Figure 6C ERα and GAPDH

Supplementary Figure 6E

ERα

78kDa

62kDa

50kDa

38kDa

28kDa

22kDa

GAPDH

78kDa

62kDa

50kDa

38kDa

28kDa

22kDa

Full unedited blot for Supplementary Figure 6E ERα and GAPDH

Supplementary Figure 6G

78kDa

62kDa

50kDa

38kDa

28kDa

22kDa

ERα

ERα

GAPDH

78kDa

62kDa

50kDa

38kDa

28kDa

22kDa

Full unedited blot for Supplementary Figure 6G ERα and GAPDH

Supplementary Figure 6I

78kDa

62kDa

50kDa

38kDa

28kDa

22kDa

ERα

78kDa

62kDa

50kDa

38kDa

28kDa

22kDa

GAPDH

Full unedited blot for Supplementary Figure 6I ERα and GAPDH

Supplementary Figure 7I

GAPDH

GAPDH

113kDa78kDa

62kDa

50kDa

38kDa

28kDa

22kDa

113kDa78kDa

62kDa

50kDa

38kDa

28kDa

22kDa

Full unedited blot for Supplementary Figure 7I PSD95 and GAPDH

Supplementary Figure 7K

PSD95

113kDa78kDa

62kDa

50kDa

38kDa

28kDa

22kDa

GAPDH

113kDa78kDa

62kDa

50kDa

38kDa

28kDa

22kDa

Full unedited blot for Supplementary Figure 7K PSD95 and GAPDH
